# Neuroprotective Potential of *Allium sativum* against Monosodium Glutamate-Induced Excitotoxicity: Impact on Short-Term Memory, Gliosis, and Oxidative Stress

**DOI:** 10.3390/nu12041028

**Published:** 2020-04-09

**Authors:** Suzan M Hazzaa, Seham Ahmed Mohamed Abdelaziz, Mabrouk A Abd Eldaim, Mohamed M. Abdel-Daim, Ghada E Elgarawany

**Affiliations:** 1 Department of Medical Physiology, Faculty of Medicine, Menoufia University, Shebeen Elkom 32511, Egypt; 2 Department of Histology, Faculty of Medicine, Menoufia University, Shebeen Elkom 32511, Egypt; 3 Department of Biochemistry and Chemistry of Nutrition, Faculty of Veterinary, Menoufia University, Shebeen Elkom 32511, Egypt; 4 Department of Zoology, College of Science, King Saud University, P.O. Box 2455, Riyadh 11451, Saudi Arabia; 5 Pharmacology Department, Faculty of Veterinary Medicine, Suez Canal University, Ismailia 41522, Egypt

**Keywords:** MSG, excitotoxicity, *Allium sativum*, gliosis, cognitive, oxidative stress

## Abstract

This study evaluated the neuroprotective potential of *Allium sativum* against monosodium glutamate (MSG)-induced neurotoxicity with respect to its impact on short-term memory in rats. Forty male Wistar albino rats were assigned into four groups. The control group received distilled water. The second group was administered *Allium sativum* powder (200 mg/kg of body weight) orally for 7 successive days, then was left without treatment until the 30th day. The third group was injected intraperitoneally with MSG (4 g/kg of body weight) for 7 successive days, then left without treatment until the 30th day. The fourth group was injected with MSG in the same manner as the third group and was treated with *Allium sativum* powder in the same manner as the second group, simultaneously. Phytochemical analysis of *Allium sativum* powder identified the presence of diallyl disulphide, carvone, diallyl trisulfide, and allyl tetrasulfide. MSG-induced excitotoxicity and cognitive deficit were represented by decreased distance moved and taking a long time to start moving from the center in the open field, as well as lack of curiosity in investigating the novel object and novel arm. Moreover, MSG altered hippocampus structure and increased MDA concentration and protein expression of glial fibrillary acidic protein (GFAP), calretinin, and caspase-3, whereas it decreased superoxide dismutase (SOD) activity and protein expression of Ki-67 in brain tissue. However, *Allium sativum* powder prevented MSG-induced neurotoxicity and improved short-term memory through enhancing antioxidant activity and reducing lipid peroxidation. In addition, it decreased protein expression of GFAP, calretinin, and caspase-3 and increased protein expression of Ki-67 in brain tissues and retained brain tissue architecture. This study indicated that *Allium sativum* powder ameliorated MSG-induced neurotoxicity through preventing oxidative stress-induced gliosis and apoptosis of brain tissue in rats.

## 1. Introduction

Food additives are considered a big problem in the food industry and should be subjected to periodical evaluation. Monosodium glutamate (MSG) is a glutamic acid salt that act as a neurotransmitter in the brain [1]. It is one of the food additives that included in many food products in order to enhance their flavor. Higher intake of MSG builds up high level of glutamic acid in blood [2]. Glutamic acid plays important roles in many physiological processes in the body as it is an excitatory neurotransmitter in the central nervous system (CNS), an energy source for some tissues, and a precursor for synthesis of glutathione [3]. Glutamate (Glu) has many receptors in the brain tissue, including the hippocampus; thus, daily intake of excess amounts of glutamic acid in food in the form of MSG impairs cell growth and induces hyperphagia, obesity, and many serious alterations in different organs. Moreover, it is neurotoxic to brain neurons, including those in the hypothalamus and hippocampus [1,4,5,6,7,8,9]. Monosodium glutamate toxicity was first discovered by Olney [10], who observed neural necrosis after MSG administration to neonatal rats. Neuronal necrosis and damage were induced by acute increase in Glu in many brain areas including the hippocampus after MSG administration at high concentrations. Hence, Glu can be considered as a “double-edged weapon” that can be converted from a neurotransmitter to a neurotoxin [11]. In addition, accumulation of Glu in the synaptic cleft has been described as excitotoxicity, which is a pathological condition where nerve cells are damaged and destroyed by excessive stimulation by excitatory neurotransmitters [12] because binding of Glu with its receptors allows an influx of large amounts of calcium ions (Ca^2+^) with consequent induction of apoptotic processes and neural degenerations [13]. In addition to this, neuronal necrosis may result from persistent depolarization of the neuron by excessive Glu receptor activation [14]. Monosodium glutamate administered systemically to neonatal rats has been broadly used as an excitotoxicity model [15]. 

Astrocytes are essential for survival of neuronal cells of CNS because they have a neuroprotective activity through developing the mitochondria-derived inclusions in aged neurons and that which is exposed to degeneration [16] (Schipper, 2004), and the astrocyte contents of reduced glutathione protects neurons from harmful effects of oxidative stress (Dringen, 2000) [17]. In addition, glutamate transporters in astrocytes are down-regulated in the mutant superoxide dismutase 1° (mSOD1) model and in human exposed to oxidative stress [18] (Rao et al., 2003). In cases of neuronal damage, astrocytes react by overexpression of the glial fibrillary acidic protein (GFAP), an intermediate cytoskeletal filament protein specific for astrocytes [19]. GFAP provides structural support and protects neurons from toxically and traumatically induced astrocyte damage [20,21] (Liedtke et al., 1996; Pekny et al., 1995). Glial fibrillary acidic proteins serve as markers for neural tissue damage as their expression is up-regulated in response to CNS injury [22] (Sofroniew and Vinters, 2010), and absence of GFAP in GFAP-/- mice increases their susceptibility to autoimmune inflammatory disease [23] (Liedtke et al., 1998). Thus, the neuroprotective role of astrocytes makes them a target for drug therapy and transplantation [24] (Darlington, 2005). Calretinin (CR), a calcium binding protein, is produced from neurons and exerts a neuro-protective effect through buffering excess free Ca2+ ions in the neurons to reduce excitotoxicity [25].

Neuroprotection is a therapeutic strategy that minimizes toxic effects on the brain [26]. Recent trends in treating diseases prefer using natural compounds in order to avoid side effects of synthetic drugs. Garlic (*Allium sativum*), family Alliaceae, is one of the natural products that have been used for long period of time as a food additive [27]. Garlic has been used to treat different diseases such as atherosclerosis, hypertension, and bronchial asthma, and has antiseptic and gastro-protective activities [28]. Garlic has many biological active sulfur compounds, such as diallyl disulphide (DAD) and diallyl trisulfide (DAT), which are responsible for its nutraceutical values. They are released by decomposition of allicin and are responsible for garlic’s characteristic odor [29]. Although *Allium sativum* has been shown to improve the cognitive function of the brain and has neuroprotective effects against amyloid β protein-induced memory impairment in mice due to its antioxidant activities and scavenging reactive oxygen species (ROS) [30], to the best of our knowledge, studies concerning its neuroprotective effects against MSG-induced excitotoxicity are still scarce. Therefore, this study was carried out in order to evaluate the neuroprotective potential of garlic extract against MSG-induced neurotoxicity with respect to its impact on short-term memory, oxidative stress, gliosis, and brain tissue structure.

## 2. Materials and Methods

The experimental protocol was approved by the local ethics committee of Faculty of Medicine, Menoufia University with approval code 412/019. Animals were treated in accordance with Guide for the Care and Use of Laboratory Animals (eighth edition, National Academies Press) [31].

### 2.1. Preparation of Allium sativum Powder

*Allium sativum* powder was prepared at the Department of Medical Physiology, Faculty of Medicine, Menoufia University, Egypt. Briefly, garlic skin was removed and the bulbs and cloves were separated. The peeled cloves were cut into thin slices and placed in a food dehydrator. Then, the slices were dried, crushed, cooled, and grinded into fine powder. The powder was stored in an airtight container and kept in cool dry place until usage [32].

### 2.2. Gas Chromatography–Mass Spectrometry (GC-MS) Analysis of Allium sativum Powder

The phytochemical analysis of *Allium sativum* powder was performed by using a Trace GC1300-TSQ mass spectrometer (Thermo Scientific, Austin, TX, USA) with a direct capillary column TG–5MS (30 m × 0.25 mm × 0.25 µm film thickness). The components were identified by comparing their retention times and mass spectra with those of the WILEY 09 and NIST 11 mass spectral databases.

### 2.3. Experimental Design

Forty male Wistar albino rats, 1 month of age (40 ± 5 g), were used in this experiment. Rats were fed with a standard laboratory diet and water ad libitum. The animals were housed in the animal house at Faculty of Medicine, Menoufia University, under a normal light/dark cycle and room temperature. The animals were assigned into 4 groups of 10 rats each.

First group: Rat received distilled water (DW) orally and injected intraperitoneally with 1ml of physiological saline/day for 7 successive days and kept as a normal control group.

Second group (*Allium sativum* group): *Allium sativum* powder was dissolved in DW at a dose of 200 mg/kg and administered orally for 7 successive days, then left without treatment until the 30th day of the experiment [28].

Third group (MSG group): Rats were injected intraperitoneally with MSG (Sigma-Aldrich Co, St. Louis, MO, USA.) Sigma-Aldrich,) dissolved in physiological saline 4 g/kg of body weight for 7 successive days, then left without treatment until the 30th day of the experiment [33].

Fourth group (*Allium sativum*-treated MSG group): Rats were injected with MSG, as was done for the third group, and were treated with *Allium sativum* powder, as was done for the second group, simultaneously. At the end of the experiment, memory and behavioral tests on modified T-maze and open field were performed. Then, rats were decapitated and brains were excised and dissected into two halves. One half was used for estimation of malondialdehyde (MDA) and superoxide dismutase (SOD). The other half was used for histopathological and immunohistochemical investigations. 

### 2.4. Behavioral Tests

All tests were performed between 9:00 a.m. to 2:00 p.m. in a soundless observation room with normal daylight. Rats were habituated in the observation room for 1 h before the beginning of the tests. All tests were observed through a video camera (Samsung ST93 Digital Camera, Suwon, South Korea). The equipment were cleaned by 70% ethanol to avoid odor cues for animals [34].

#### 2.4.1. Assessment of Motor Function

A wooden arena (100 × 100 × 60 height, brown wall, and floor) was divided into 25 squares (20 cm per square). Rat was put in the center of the arena for 15 min and freely allowed to explore it [34]. Latency to move from the center was observed, and the total distance moved by meter (m) was assessed by calculating the numbers of crossed squares. 

#### 2.4.2. Assessment of Short-Term Spatial Memory

##### Novel Object Discrimination

**Figure 1:**
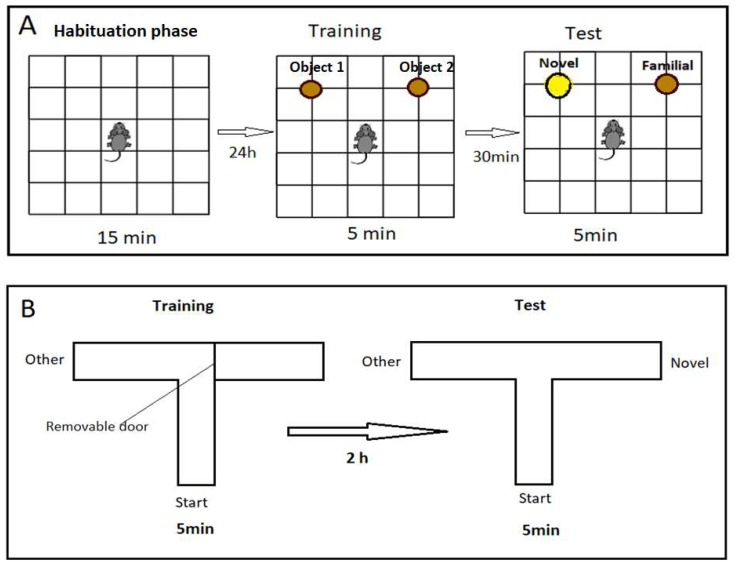
Schematic illustration of the different session. (**A**) Open field (habituation) for 15 min, training and test sessions for novel object discrimination. (**B**) Modified T-maze, training trial for 5 min in start and other arms. Test trial for 5 min in start, other and novel arms, 2 h interval.

This consisted of three phases: habituation, training, and test phases. Habituation was carried out in the open field without any object for 15 min before testing short-term memory by 24 h to reduce anxiety and stress. Training and test phases were carried out for 5 min and 30 min intervals between the two phases in order to assess short-term memory. In the training phase, rats were allowed to recognize two identical cylindrical objects located 15 cm from the wall and 60 cm apart from each other. In the test phase, a novel cylindrical object replaced one of the familiar objects, as shown in Figure 1. The time spent per second by each rat to explore the objects was calculated [35].

##### Modified T-Maze

Modified T-maze was used to evaluate short-term spatial memory on the basis of rats’ innate preferences to discover new areas that had not been previously discovered. The apparatus consisted of three wooden arms (50 cm long × 10 cm wide × 20 cm high) located 50 cm above the floor. The task consisted of two training trials and test trials of 5 min duration and 2h inter-trial interval. In the training trial, the novel arm was closed by removable door. Rats were located in the start arm facing the center, and they were allowed to move freely between the start and other arms. In the test trial, the novel arm was opened, and rats were free to move in all arms. Percentage of entry, as detected by the presence of four paws of rats in training and test trials in all arms as well as percentage of time (in seconds) spent in each arm, was calculated [36].

### 2.5. Measurement of Brain Tissues Malondialdehyde Concentration and Superoxide Dismutase Activity

Brain tissues were taken and homogenized in normal saline solution (1:9 w/v). Then, the homogenate was centrifuged at 1800 × *g*/min for 10 min. The supernatant was used for detection of lipid peroxidation and antioxidant enzyme activity via biomarkers MDA and SOD, respectively. Malondialdehyde concentration and SOD activity were measured calorimetrically by using commercial kits (Biodiagnostic Company, Cairo, Egypt). Malondialdehyde was measured by using the thiobarbituric acid reaction [37]. Superoxide dismutase was measured as described by [38]. 

### 2.6. Histological Examination

Brain tissue samples of all groups were rapidly excised, cut into small pieces, and fixed in 10% neutral formalin. Then, tissue sections were dehydrated in ascending concentrations of alcohol, cleared in xylene, and finally embedded in paraffin. Serial sections of about 5μm in thickness were prepared and stained by hematoxylin and eosin (H&E) staining for demonstration of the histopathological changes in brain tissues [39].

### 2.7. Immunohistochemical Investigations

Glial fibrillary acidic protein, caspase-3, and Ki-67 proteins were localized immunohistochemically by using avidin–biotin complex (ABC) immunoperoxidase technique. After blocking the endogenous peroxidase, brain sections were incubated with primary antibodies for 20 min at room temperature (the primary anti-GFAP antibody at 1:100 dilution; caspase-3 antibodies at 3 μg/mL with 1:200 dilution, and Ki67 antibody at 1:100 dilution). The primary GFAP and Ki-67 antibodies were mouse monoclonal antibodies, (GFAP) Ab-1 (Clone GA-5), specific to the astrocytes obtained from Lab Vision Corporation, Medico Co., Egypt (catalog. #MS- 280-B0 and for Ki-67). Caspase-3 antibody reacts broadly with all known caspase-3 variants of human, rat, and mouse origin by immunohistochemistry (Lab Vision Corporation, Fremont, CA, USA). Then, the slides were washed with diluted phosphate-buffered saline (PBS) and incubated with the secondary anti-mouse antibody universal kits for 30 min in a humid chamber at room temperature. 

To demonstrate the immunoreactivity of calretinin (CR) in neurons, indirect immunohistochemical peroxidase–antiperoxidase reaction (PAP) was performed. Brain sections were treated with 0.4% H_2_O_2_ at room temperature for 30 min to inhibit the endogenous peroxidase. After rinsing in 0.5 M trisaminomethane (TRIS) buffer (TBS, pH = 7.6), the sections were incubated in normal goat serum at room temperature for 20 min in order to eliminate background staining. A set of antibodies and reagents (Sigma-Aldrich, St. Louis, MO, USA) were diluted with 0.5 M TBS according to the producer’s recommendations and were used to conduct immunohistochemical PAP reaction. The primary antibody was specific monoclonal rabbit anti-CR antibody (incubation for 48 h at 4 °C), whereas the secondary antibody was monoclonal goat anti-immunoglobulin G (IgG) antibody (Sigma-Aldrich). Finally, the monoclonal peroxidase–antiperoxidase complex was applied.

All sections were stained by incubation with 3,3’-diaminobenzidine (DAB), a substrate chromogen, for 5–10 min. One DAB tablet was dissolved in 10 mL PBS. Chromogen resulted in brown-colored precipitate at the antigen sites, and Mayer’s haematoxylin was used as a counter stain. Positive control was Cellosaurus cell line (IMR5) cells in the brain. For negative controls, incubation was carried out with the removal of the primary antiserum. The positive reactivity of GFAP, caspase-3, Ki-67, and calretinin were exhibited as different grades of reactivity (i.e., weak, moderate, and strong), according to the intensity of staining. Their positive reactivity was indicated by a brown-colored reaction [40]. 

For image capturing, a colored video camera (Panasonic Color CCTV camera, Matsushita Communication (Industrial Co. Ltd., Tokyo, Japan) fixed on a light microscope (Olympus BX-40, Olympus Optical Co. Ltd., Tokyo, Japan) was used. Images were taken at X400 magnification and 2.6 zoom. Photomicrographs were analyzed by using Software Image J program, a public domain image processing and analysis program (http://rsb.info.nih.gov/ij/) [41].

### 2.8. Statistical Analysis

The data are expressed as mean ± standard error of the mean (SEM). The statistical analysis was conducted by using SPSS version 22. The behavioral tests on the open field and modified T-maze were performed by using Kruskal–Wallis test and Mann–Whitney test. Other results were analyzed by using one-way ANOVA (analysis of variance), followed by post hoc (Tukey’s) test. The difference of variance at *p* < 0.05 was considered significant.

## 3. Results

### 3.1. The Phytochemical Components of Allium sativum Powder

**Figure 2:**
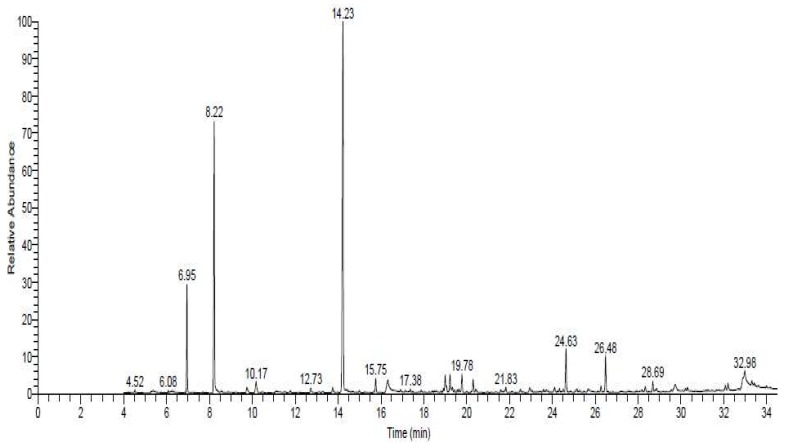
The spectrum of identified compounds from *Allium sativum powder* by using Trace GC1300-TSQ mass.

Phytochemical analysis of *Allium sativum* powder indicated the presence of diallyl disulphide, carvone, diallyl trisulfide, allyl tetrasulfide, and 1-allyl-3-(2-(allylthio) propyl) trisulfane (Figure 2, Table 1 and Supplementary Materials File S1).

### 3.2. Allium sativum Improved Motor Functions of Rats Administrated MSG

**Figure 3:**
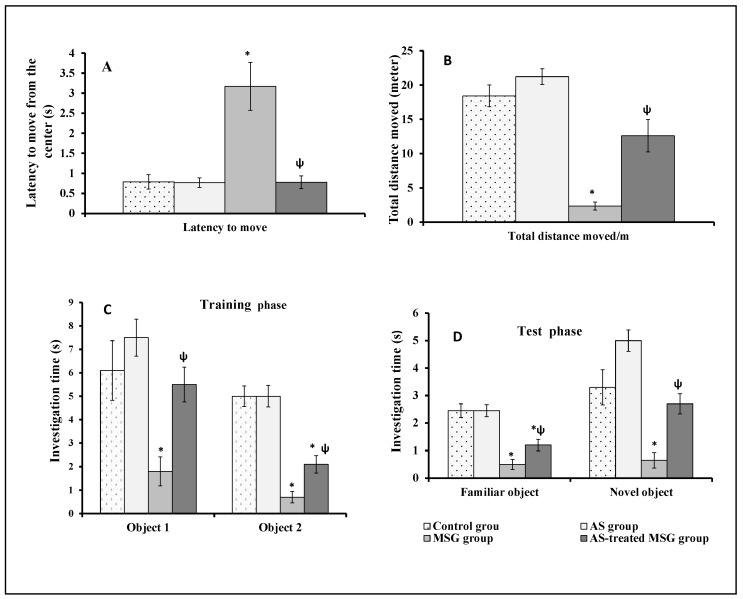
Effect of MSG and AS on Motor functions (**A**,**B**) and short-term spatial memory (**C**,**D**) by open field test in different studied groups: (**A**) the latency to move from the center; (**B**) the total distance moved (in meters); (**C**) the time of investigation of the two objects in the training phase/s; (**D**) the time of investigation of the familiar and novel objects in the test phase (in seconds). Data are expressed as mean ± SEM (*n* = 10). * *p* < 0.05, vs. control; ψ *p* < 0.05, vs. monosodium glutamate (MSG).

Figure 3 showed the effect of MSG and/or *Allium sativum* powder on rats’ motor functions. MSG significantly (*p* < 0.001) reduced the locomotor activity of rats by increasing the latency time to move from the center (Figure 3A) and decreased the total distance moved in open field test compared to control group (Figure 3B). However, simultaneous administration of MSG and *Allium sativum* powder to rats of the fourth group significantly (*p* < 0.001) decreased the latency time to move from the center and increased the total distance moved in open field test compared with the MSG-administered group (third group) (Figure 3A,B).

### 3.3. Allium sativum Modulated the Effect of MSG on Short-Term Spatial Memory of Rats

Figure 3 shows the effect of MSG and/or *Allium sativum* on short-term spatial memory of rats. In the novel object discrimination task in open field, MSG significantly (*p* < 0.05 and *p* < 0.001, respectively) decreased the time for investigation of objects 1 and 2 during the training phase compared with the control group (Figure 3C). In the test phase, the time for investigation significantly (*p* < 0.001 and *p* < 0.01, respectively) decreased for both the familiar and novel objects compared with the control group (Figure 3D). In contrast, administration of MSG and *Allium sativum* powder simultaneously to rats (fourth group) significantly (*p* < 0.01) increased the time for investigation of objects 1 and 2 in the training phase and the time for investigation of both the familiar and novel objects in the test phase compared with rats administered with MSG (third group) (Figure 3C,D). 

**Figure 4:**
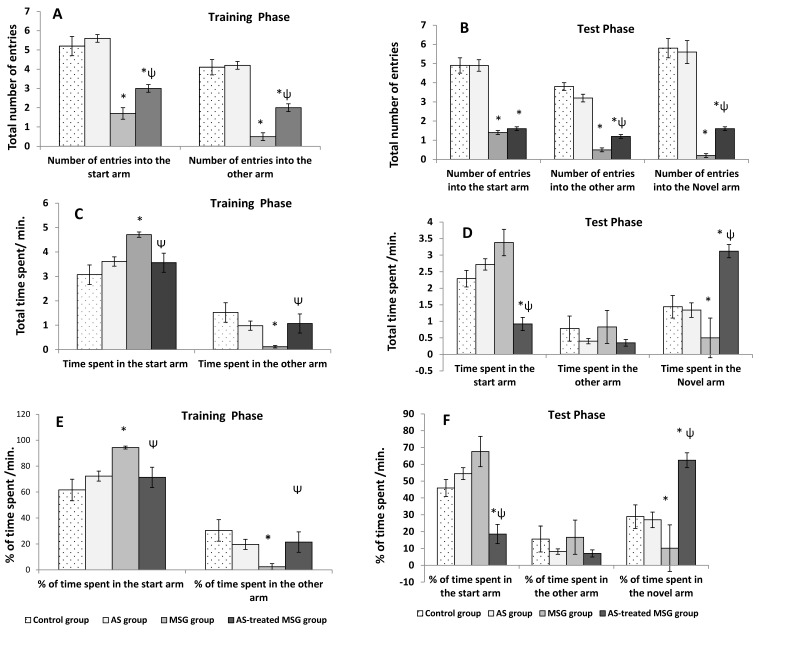
Short-term spatial memory tested by modified T-maze in different studied groups: (**A**) the number of entries in the training phase; (**B**) the number of entries in the test phase; (**C**) the time spent in the training phase; (**D**) the time spent in the test phase; (**E**) the percentage of time spent in the training phase; (**F**) the percentage of time spent in the test phase. Data are expressed as mean ± S.E.M. (*n* = 10). * *p* < 0.05, vs. control; ψ *p* < 0.05, vs. MSG.

In the T-maze test, MSG significantly decreased (*p* < 0.001) the number of entries into the start and other arms in the training and test phases as well as the novel arm in the test phase compared with the control rats (Figure 4A,B). In addition, it significantly increased (*p* < 0.001) the time spent and percentage of time spent in the start arm, whereas it significantly decreased (*p* < 0.001) the time spent in the other arm during the training phase compared with the control group. In the test phase, the MSG-treated group showed insignificant increase in the time spent and percentage of time spent in the start and other arms, but showed a significant (*p* < 0.01) decrease in the time spent and percentage of time spent in the novel arm compared to both control and powder groups (Figure 4C–F). However, treatment of MSG-administered rats with *Allium sativum* powder (fourth group) significantly (*p* < 0.01) increased the total number of entries into different arms compared with the MSG-treated group (third group) during training and test phases (Figure 4A,B). Additionally, *Allium sativum* powder (fourth group) significantly (*p* < 0.01) increased the time spent and percentage of time spent exploring the novel arm compared to the MSG-treated group (third group) during test phases (Figure 4D,F).

Monosodium glutamate significantly increased (*p* < 0.05) the brain tissue lipid peroxidation biomarker MDA content, whereas it significantly decreased the activity of SOD in brain tissue compared with the control group. However, administration of MSG and *Allium sativum* powder simultaneously to rats significantly (*p* < 0.05) decreased concentration of MDA while increasing the activity of SOD in brain tissue compared with the MSG-administered group (third group) (Table 2).

### 3.4. Allium sativum Modified MSG-Induced Alterations in Brain Tissue Architecture of Rats

**Figure 5:**
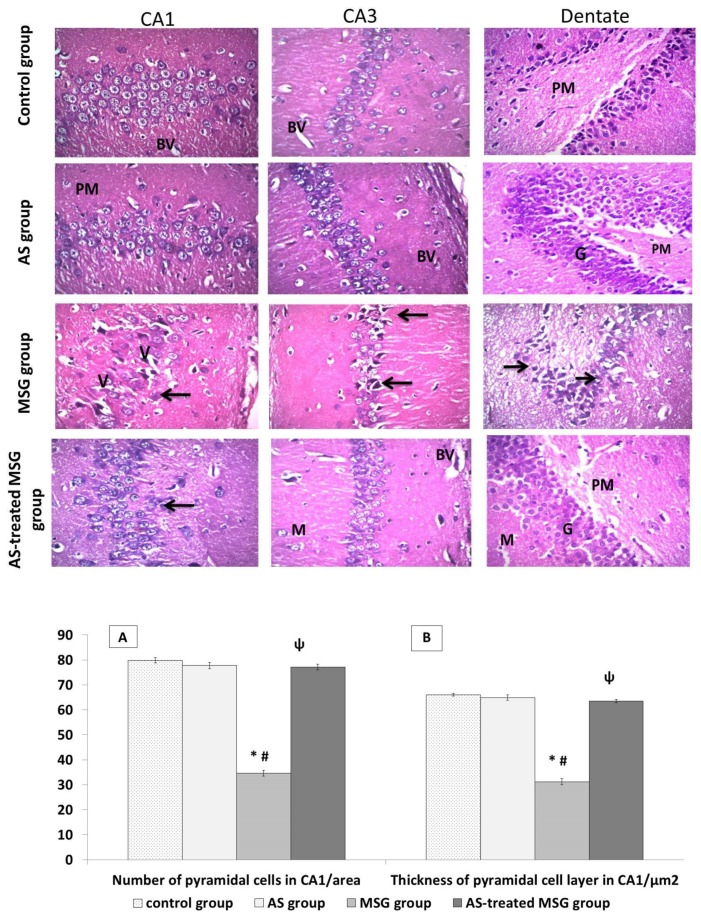
Light microscopic examination of hematoxylin and eosin (H&E)-stained sections in all groups. Control and *Allium sativum* (AS) groups showed normal histological structure of hippocampus. Monosodium glutamate (MSG) group showed decrease in the number of the cells in Cornu Ammonis° (CA1 and CA3) areas with shrinkage and disarrangement of pyramidal cells. Apoptotic and pyknotic nuclei of pyramidal cells surrounded with pericellular haloes. Dilated blood vessels and massive vacuolation of the cytoplasm (V) can be also seen. Dentate gyrus of the same group showed many apoptotic cells (arrows), darkly stained nuclei, vacuolated cytoplasm (V), and disorganized blood vessels (BV). *Allium sativum*-treated MSG (AS-treated MSG) group was more or less like the control group, with multiple dilated blood vessels. (Hx. &E. X400). Histograms (**A**) and (**B**) indicate significant decrease in pyramidal cell count and layer thickness in the CA1 region in the MSG group compared to the control and AS groups. In addition, the AS-treated MSG group showed a significant increase in these parameters compared to the MSG group. * *p* < 0.05, vs. control; ψ *p* < 0.05, vs. MSG.

Microscopic examination of brain tissues of both control and powder-treated rats stained with H&E revealed normal histological structure of the hippocampus (Figure 5). However, administration of rats with MSG significantly decreased the thickness of the pyramidal cell layer and cell count in Cornu Ammonis (CA1 and CA3) areas compared with those of the control group. In addition, pyramidal cells showed disarrangement, degeneration, and pyknosis of their nuclei with loss of pyramidal cells, leaving empty spaces filled with vacuolations in the surrounding neutrophils. The dentate gyrus of this group showed marked reduction in number of cells of all three layers with disarrangement of granular cells, appearance of many apoptotic cells, and darkly stained nuclei with vacuolations of their cytoplasm (V) (Figure 5).

Treatment of MSG rats with *Allium sativum* powder significantly increased the thickness of pyramidal cell layer and cell count in CA1 area compared with the MSG group. The pyramidal layer of CA1 and CA3 areas revealed improvement of the histological picture, and most of the pyramidal cells appeared normal with euchromatic vesicular nuclei. However, some pyramidal cells were seen in the polymorphic layer, and a few other cells showed pyknosis of their nuclei with dilated blood vessels. Dentate gyrus of the same group revealed improvement of the histological picture of all layers (Figure 5).

### 3.5. Allium sativum Reduced MSG-Induced GFAP and Calretinin Protein Expression While Increasing Ki-67 Protein Expression in Brain Tissue of Rats 

**Figure 6:**
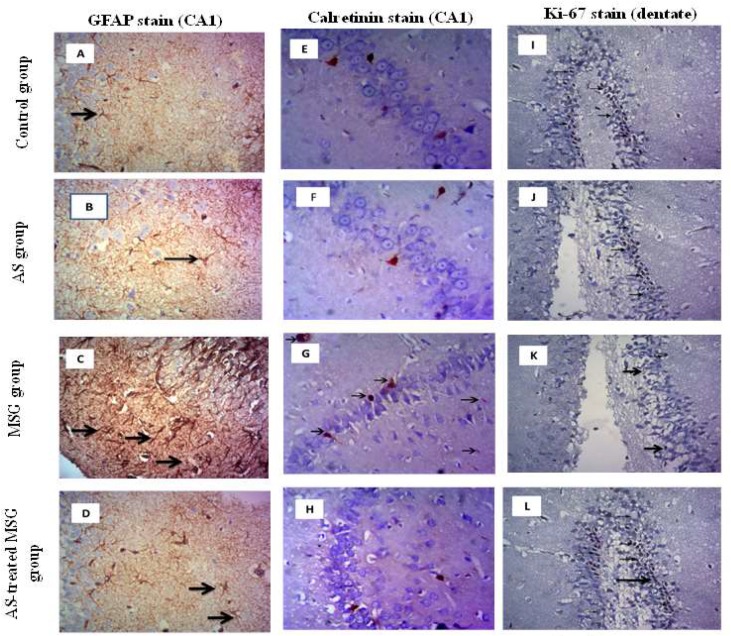

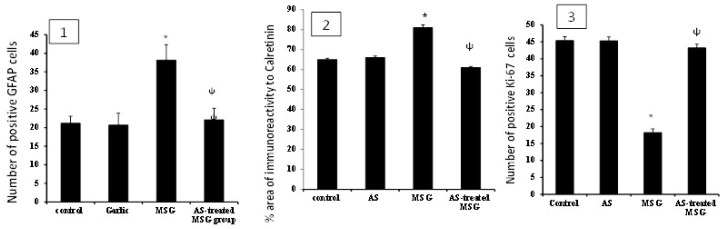
Light microscopic examination of glial fibrillary acidic protein (GFAP)-stained sections of CA1 in the hippocampus (**A**–**D**) showing strong positive immune staining in MSG group compared with the control and AS groups. AS-treated MSG group showed weak reaction (arrows) (GFAP X400). Calretinin-stained sections of CA1 in the hippocampus (**E**–**H**) showed weak immune positive reactive neurons in control, AS, and AS-treated MSG groups, but MSG group showed strong positive reactive neurons (arrows) (Calretinin X200). Additionally, light microscopic examination of Ki-67-stained sections of the dentate of hippocampus (**I**–**L**) showed marked positive immune stain in the sub-granular zone (arrows) in the control and AS groups. The number of Ki-67 immunoreactive nuclei was significantly decreased in the MSG group compared to the control group. AS-treated MSG group was more or less like the control group (Ki-67 X400). * *p* < 0.05, vs. control; ψ *p* < 0.05, vs. MSG.

Administration of MSG to rats significantly increased GFAP immune reaction in the hippocampus, indicating an increase in astrocyte number and cell processes. This was shown by the intensity of brown color that meant active gliosis compared with the control group. However, treatment of MSG-administered rats (fourth group) with *Allium sativum* powder significantly decreased GFAP immunoreactivity in astrocytes and their processes compared to MSG-administered rats (third group) (Figure 6A–D).

In addition, MSG induced strong calretinin immune reactivity in neurons, which was indicated by the presence of cytoplasmic and nuclear reaction. However, brain section of rats of the normal control, second, and fourth groups showed mild reaction (Figure 6E–H).

Investigating the protein expression of Ki-67 in brain sections of different groups showed positive nuclei in the sub-granular zone of the dentate gyrus of the control and *Allium sativum*-treated groups (first and second groups). The number of Ki-67 immunoreactive nuclei was significantly decreased in the MSG-administered group (third group) compared to the control group. However, treatment of the MSG-administered group with *Allium sativum* powder (fourth group) normalized the expression of Ki-67 protein in brain tissue (Figure 6I–L).

**Figure 7:**
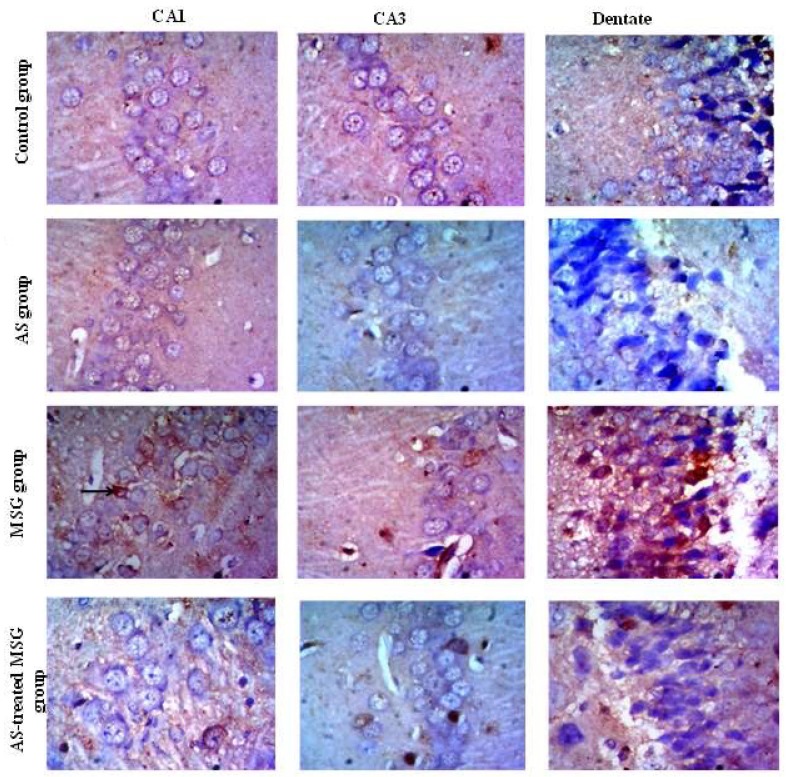
Light microscopic examination of caspase-3 stained sections of CA1, CA3, and dentate gyrus of the hippocampus. The figure shows weak cytoplasmic immune staining reaction in all areas in control and AS groups. The MSG group showed strong positive cytoplasmic immune staining reaction (arrows) in all areas, whereas the AS-treated MSG group showed moderate reaction (caspase-3 X400).

The immunohistochemical detection of caspase-3 in brain tissue of different groups showed that the expression of caspase-3 protein in CA1, CA3, and dentate gyrus of brain tissue of the control and *Allium sativum* groups was mild. Administration of rats of the third group with MSG increased caspase-3 protein expression in CA1, CA3, and dentate gyrus. However, treatment of MSG-administered rats (fourth group) with *Allium sativum* powder reduced caspase-3 protein expression in CA1, CA3, and dentate gyrus compared to that of the third group (Figure 7). 

## 4. Discussion

Monosodium glutamate is one of the food additives that has been used over the centuries, especially in Chinese and Asian dishes [42]. The results of the current study revealed that injection of rats with MSG induced excitotoxicity and cognitive deficit, which was indicated by hypoactivity in the form of decreased distance moved in open field, taking a long time to start moving from the center, and lack of curiosity in investigating novel object and novel arm during the test for short-term spatial memory by open field and T-maze. These results were in line with that of [43], who demonstrated that MSG has an excitotoxic effect on the brain with significant long-term damage of many brain areas, especially if administered in early life. In addition, Hassaan et al. [44] reported that MSG intake in early life induced neurotoxicity that impairs the short-term memory and affects the exploratory behavior in mice. These exitotoxic effects of MSG may be due to the dissolving of MSG in water, dissociating it into sodium and glutamate ions, which increases the plasma glutamate levels up to 17-fold higher than the normal basal level [43]. Further, injection of animals with MSG increases the extracellular brain glutamate concentration and induces motor and behavioral alterations [15] due to glutamate reacting with its receptors, as well as initiating apoptosis and necrosis of neuronal cells through over-activation of glutamate receptors. Activation of glutamate receptors releases Ca^+2^ from its stores, which causes mitochondrial over-activation and stimulates a number of intracellular enzymes such as endonucleases, phospholipases, and proteases. These enzymes damage cell structures including the cytoskeleton, cell membrane, and DNA [6]. Another study demonstrated that treatment of rats with MSG leads to cognitive deficit with hypoactivity and irritability, as it induced hippocampal degeneration, impaired synaptic plasticity, and caused a deficit in short-term and long-term potentiation [45]. Another possible cause of the excitotoxic effect of MSG is the oxidative stress that induced in the hippocampus, evidenced by elevation of MDA concentration and concomitant decrease in SOD activity in brain tissues (Table 2) because oxidative stress is a characteristic feature of neurodegenerative disease. It easily affects the brain because the brain has a high metabolic activity with low antioxidant capacity. Released free radicals cause peroxidation of cell membranes and DNA, causing cell damage and apoptosis [46]. The presence of these free radicals activates signaling pathways of inflammation and cell damage, and may cause disruption of the blood–brain barrier by affecting the endothelial basement membrane [43]. Moreover, the released free radicals contribute to excitotoxicity. Free radicals are generated from mitochondria following calcium influx due to glutamate receptor stimulation [47]. On contrast, the results of the current study indicated that *Allium sativum* modulated MSG-induced behavioral abnormality, as treatment of MSG-administered rats with *Allium sativum* powder improved motor activity and behavior and spatial memory, indicating a protective effect of *Allium sativum* against MSG-induced excitotoxicity. Behavioral and motor improvement induced by *Allium sativum* may be attributed to its contents of diallyl disulphide (DAD), diallyl trisulfide (DAT), and allyl tetrasulfide (AT) (Figure 2 and Table 1), as Tang et al. [48] found that DAT, an *Allium sativum* active constituent, inhibits colonic smooth muscle contraction in male rats that act as a channel blocker that inhibits the L-type calcium channel. The other possible cause of the improvement effects of *Allium sativumonon* on the behavior of MSG administrated rats is the antioxidant activities of its components, as [49] reported that garlic has strong antioxidant, neuroprotective, and antiapoptotic effects against many neurotoxins, and also has a neurotrophic effect on hippocampal cells due to it containing allicin [46]. Thus, we postulated that DAD and DAT decreased Ca^+2^ influxes to hippocampal neurons by blocking the L-type calcium channel, which may reduce excitotoxicity and neuronal death. 

Moreover, our data showed that MSG administration decreased the number of pyramidal cell layers in CA1 and CA3 areas of the hippocampus due to neuronal cell death. Additionally, most of the nerve cells were distorted in shape (Figure 5). These histopathological changes in brain tissues may be related to MSG-induced over-expression of caspase-3 protein and decreased Ki-67 protein expression in brain tissue of the MSG group (Figure 6 and Figure 7). The increased expression of caspase-3 protein in the hippocampus of MSG-administered rats can explain the pyramidal cell loss and neuronal degeneration. Activation of caspases had been linked to neuronal apoptosis and considered a key stimulator of cell death. Neuronal injury under the effect of neurotoxins leads to activation of cell death signals [50]. MSG-induced apoptosis in the hippocampus may be attributed to oxidative stress that leads to mitochondrial damage with release of cytochrome C and activation of caspases. However, Ki-67 is a protein marker of active cell proliferation in the subgranular zone of dentate gyrus, the active site of neurogenesis in the brain [51], indicating downregulation of neurogenesis in the MSG-administrated group. These degenerative effects greatly affected memory because they disrupted the normal flow of information from the dentate gyrus to CA3 through mossy fibers, and also may have affected the flow of information from CA3 via the Schaffer’s fibers to CA1 [26]. Our findings were in agreement with Rycerz et al. [25], who found that administration of MSG to rats in postnatal week revealed loss of 11.5% of pyramidal neurons in the hippocampus. On the other hand, MSG significantly increased astrogliosis, which was evidenced by increased GFAP expression (Figure 6), a hallmark of increased astrocyte activity as a result of injury to the central nervous system [52]. Previous data indicated that MSG administration induced hypertrophy of astrocytes and microglial cells in the hippocampus, also increasing in neuronal apoptosis in CA1 and CA3 areas, leading to cognitive impairment [43]. Therefore, the use of antioxidants is a vital process in inhibiting neuronal damage. Diallyl disulfide is an important natural antioxidant capable of recovering normal redox activity and enhances certain signaling pathways that favor protection of homeostatic response of the cell [50]. Diallyl disulfide also has been proven to be neuroprotective because it protects the brain against hypoxically and ischemically induced brain injury [53,54] in rats. This neuroprotective activity of *Allium sativum* is attributed to the ability of garlic-derived organosulfur compounds—DAD and DAT—to modulate redox activity and to reduce oxidative stress and their incorporation in many signaling pathways [47]. Diallyl disulfide was proven as a strong lipid peroxidation terminator [55], thus providing neuronal protection and preventing neuronal damage by excitotoxins. The protective effect of *Allium sativum* against MSG-induced hippocambal neurons agreed with the finding of Hwang et al. [55], who reported that garlic protected dopaminergic neurons from degeneration in a Parkinson rat model. The authors also found that it decreased astrocyte activation and neural inflammation. Another study by Rojas et al. [6] indicated that *Allium sativum* powder protected mice from neurotoxicity and oxidative damage by free radical scavenging effects and preservation of SOD activity. Diallyl trisulfide also protects motor neurons and mediates cell survival by enhancing autophagy and stimulating antioxidant enzymes [56]. Additionally, Mostafa [57] reported that DAT protected neurons and improved cognitive functions in streptozotocin-induced diabetic rats through H_2_S release. In addition, *Allium sativum* was found to significantly decrease the expression of caspase-3 pro-apoptotic protein marker and up-regulate the anti-apoptotic genes because it contains many sulfur-containing compounds and volatile oil [58]. Beside its antioxidant effect, *Allium sativum* reduces cell damage through an anti-inflammatory property and decreases mitochondrial disorders occurring in different diseases [59]. Furthermore, our results indicated that the expression of calretinin protein was increased in the brain tissue of the MSG-treated group, which is a neuroprotective protein that regulates calcium homeostasis by buffering excess calcium in cases of glutamate-induced excititoxicity [25]. Treatment with *Allium sativum* powder significantly reduced the expression of calretinin, as it reduced MSG-induced excitotoxicity. 

## 5. Conclusions

Monosodium glutamate severely affected the short-term spatial memory of rats through oxidative stress-induced degenerative changes and apoptosis of brain tissue. However, *Allium sativum* ameliorated MSG-induced degenerative and apoptotic changes in brain tissue through reducing the oxidative stress and gliosis-induced structural damage of the hippocampus and by increasing the protein expression of Ki-67 in brain tissue. This study suggested that *Allium sativum* is a potent neuroprotective agent against pollutant-induced nervous tissue damage. 

## Figures and Tables

**Table 1:** Phytochemical contents of *Allium sativum* powder.

Compounds	RT/min	Area %	MW	Formula
Diallyl disulphide (DAD)	8.22	22.29	146	C6H10S2
Carvone	12.73	0.60	150	C10H14O
Diallyl trisulfide (DAT)	14.23	38.21	178	C6H10S3
Allyl tetrasulfide (AT)	20.31	1.15	210	C6H10S4
1-Allyl-3-(2-(allylthio) propyl) trisulfane	26.48	3.46	252	C9H16S4

**Table 2:** Effects of monosodium glutamate and/or *Allium sativum* powder on malondialdehyde (MDA) concentration and superoxide dismutase (SOD) activity in brain tissue.

	Control	*Allium sativum*	MSG	*Allium sativum*-Treated MSG
MDA (nmole/mg protein)	1.6 ± 0.57 ^c^	1.25 ± 0.39 ^c^	29.5 ± 2.38* ^a^	8.5 ± 0.78 * ^ψ b^
SOD (U/mg protein)	1.066 ± 0.032^a^	1.07 ± 0.026 ^a^	0.923 ± 0.011* ^b^	1.007 ± 0.01 ^ψ b^

Means ± standard error (SE) * *p* < 0.05, vs. control; ψ *p* < 0.05, vs. MSG Values carrying different superscript letters (a, b, c) in the same row were significant different. Monosodium glutamate, MSG; malondialdhyde, MDA; superoxide dismutase, SOD.

## Supplementary Materials

The following are available online at https://www.mdpi.com/2072-6643/12/4/1028/s1, File S1: Screening of phytochemical constituents of Allium sativum by GC-MS.
