# Supplementary material for: Neuroprotective Potential of Allium sativum against Monosodium Glutamate-Induced Excitotoxicity: Impact on Short-Term Memory, Gliosis, and Oxidative Stress
Source: Nutrients. 2020 Apr 9;12(4):1028. doi: 10.3390/nu12041028 (PMC7230314; doi:10.3390/nu12041028)

My GC-MS Report

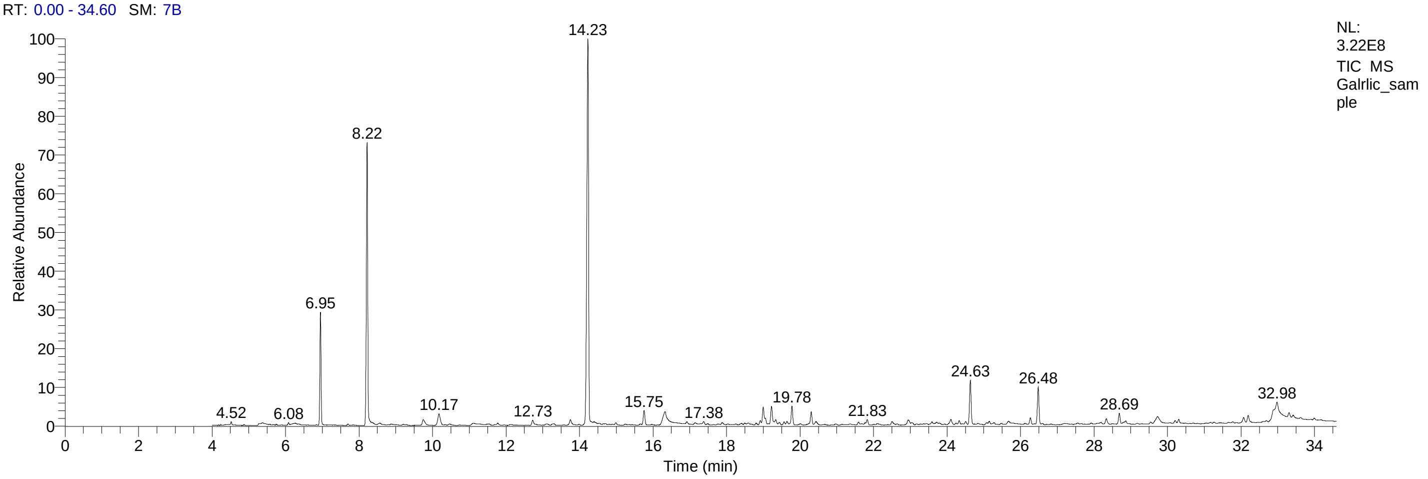

RT	Area %	Peak Area
6.95	7.76	208739729.86
8.22	22.29	599910323.45
9.75	0.74	20046735.35
10.17	1.58	42475462.22
12.73	0.60	16226020.92
13.75	0.57	15252120.19
14.23	38.21	1028260927.02
15.76	1.41	38051719.39
16.32	1.89	50895383.50
18.92	0.32	8657566.27
19.00	2.06	55307283.96
19.22	1.67	44942703.39
19.34	0.40	10739549.46
19.78	1.75	47202611.70
20.31	1.15	30895853.84
21.83	0.58	15571565.79
22.94	0.67	18030679.64
24.11	0.66	17659012.74
24.63	3.98	107034028.22
26.27	0.57	15448142.77
26.48	3.46	93044524.88
28.33	0.59	15762477.28
28.69	0.94	25244921.67
29.73	1.20	32192344.03
32.07	0.58	15609935.12
32.19	0.77	20811315.28
32.88	0.92	24723286.11
32.98	2.26	60754987.20
33.31	0.43	11585568.43

My GC-MS Report

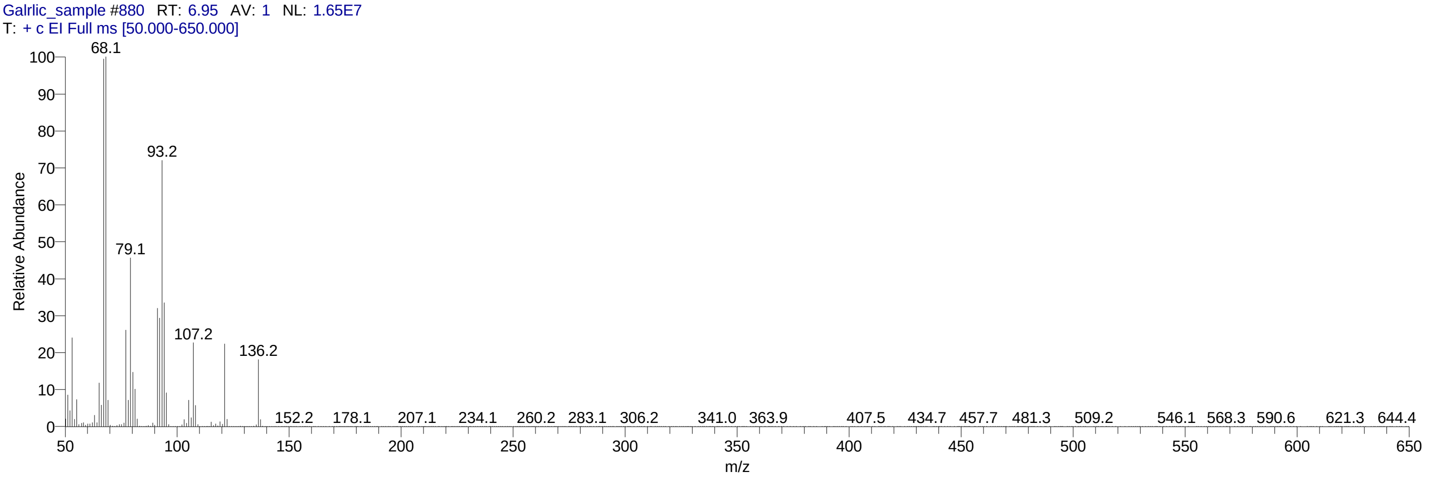

RT	Compound Name	Area %	Molecular Formula	Molecular Weight	Cas #	MF	Library
6.95	Limonene	7.76	C10H16	136	138-86-3	928	replib
6.95	Cyclohexanol, 1-methyl-4-(1-methylethenyl)-, acetate	7.76	C12H20O2	196	10198-23-9	931	replib
6.95	CYCLOHEXANOL, 1-METHYL-4-(1-METHYLETHENYL)-, ACETATE	7.76	C12H20O2	196	10198-23-9	930	WileyRegistry8e

Compound Structure	Hit Spectrum

Limonene
Formula C10H16, MW 136, CAS# 138-86-3, Entry# 8332
Cyclohexene, 1-methyl-4-(1-methylethenyl)-

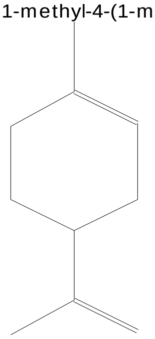

SI 927, RSI 928, replib, Entry# 8332, CAS# 138-86-3, Limonene

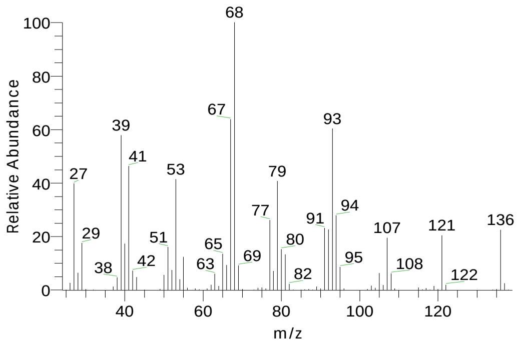

Cyclohexanol, 1-methyl-4-(1-methylethenyl)-, acetate
Formula C12H20O2, MW 196, CAS# 10198-23-9, Entry# 8355
p-Menth-8-en-1-ol, acetate

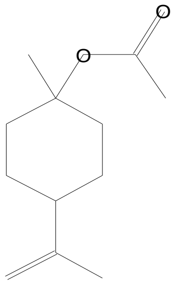

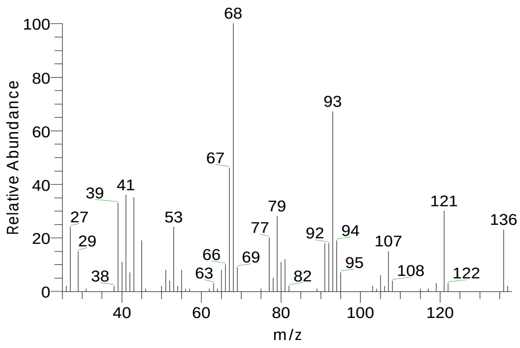

My GC-MS Report

Compound Structure | Hit Spectrum

CYCLOHEXANOL, 1-METHYL-4-(1-METHYLETHENYL)-, ACETATE
Formula C12H20O2, MW 196, CAS# 10198-23-9, Entry# 80012
4-ISOPROPENYL-1-METHYLCYCLOHEXYL ACETATE #

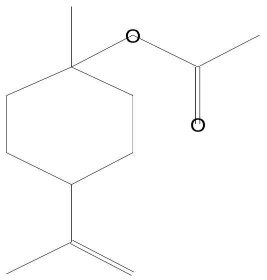

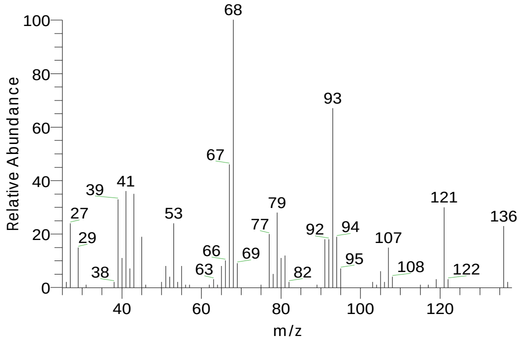

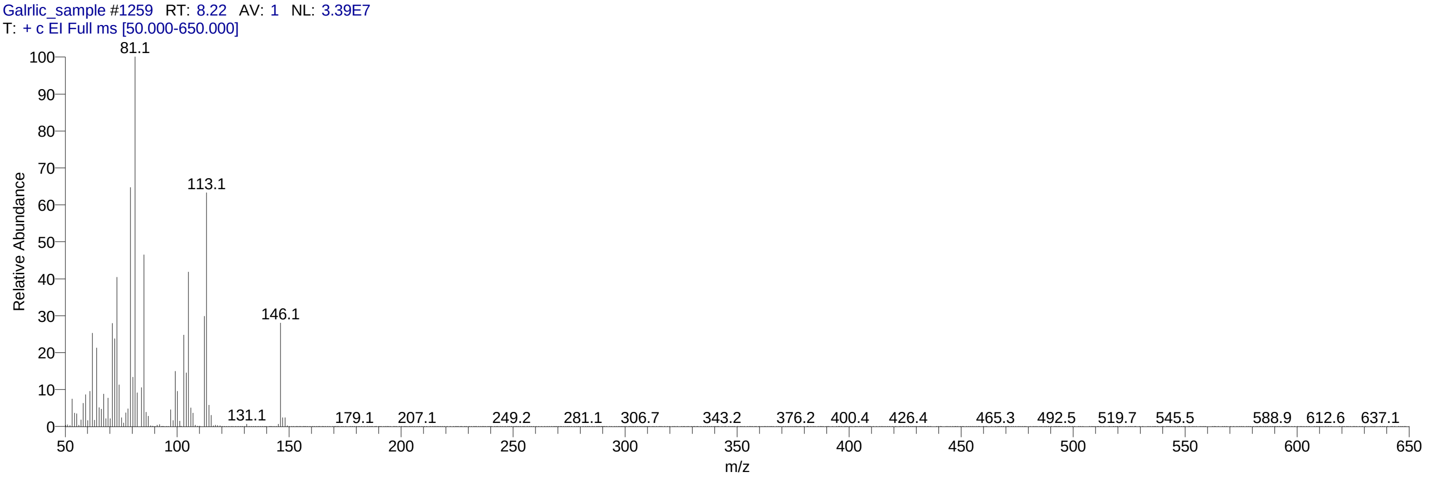

RT	Compound Name	Area %	Molecular Formula	Molecular Weight	Cas #	MF	Library
8.22	Diallyl disulphide	22.29	C6H10S2	146	2179-57-9	941	replib
8.22	DISULFIDE, DI-2-PROPENYL	22.29	C6H10S2	146	2179-57-9	842	WileyRegistry8e
8.22	Diallyl disulphide	22.29	C6H10S2	146	2179-57-9	841	replib

Compound Structure | Hit Spectrum

Diallyl disulphide
Formula C6H10S2, MW 146, CAS# 2179-57-9, Entry# 835
Allyl disulfide

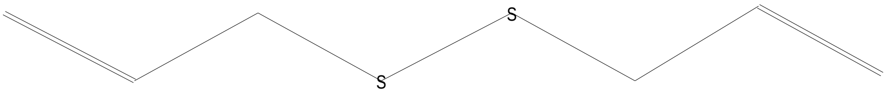

SI 941, RSI 941, replib, Entry# 835, CAS# 2179-57-9, Diallyl disulphide

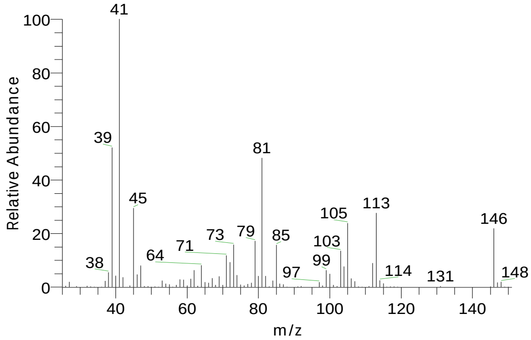

My GC-MS Report

Compound Structure | Hit Spectrum

DISULFIDE, DI-2-PROPENYL
Formula C6H10S2, MW 146, CAS# 2179-57-9, Entry# 30422
3-(ALLYLDISULFANYL)-1-PROPENE #

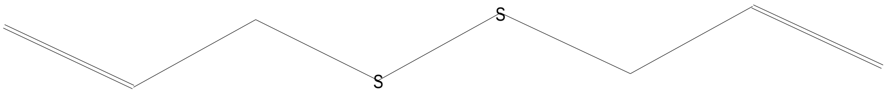

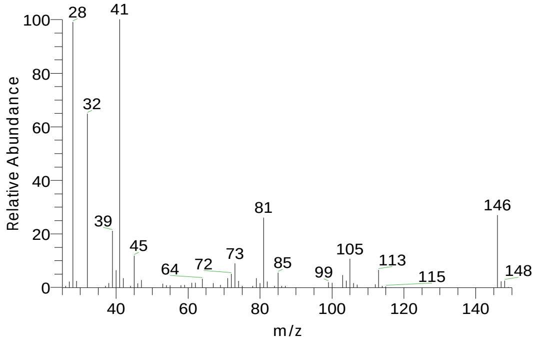

Diallyl disulphide
Formula C6H10S2, MW 146, CAS# 2179-57-9, Entry# 1395
Allyl disulfide

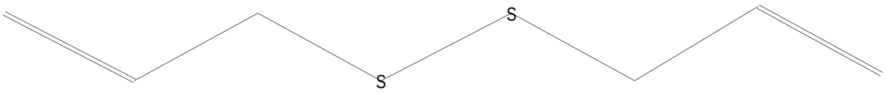

SI 820, RSI 841, replib, Entry# 1395, CAS# 2179-57-9, Diallyl disulphide

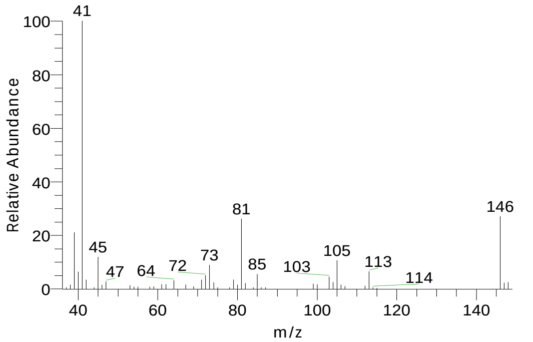

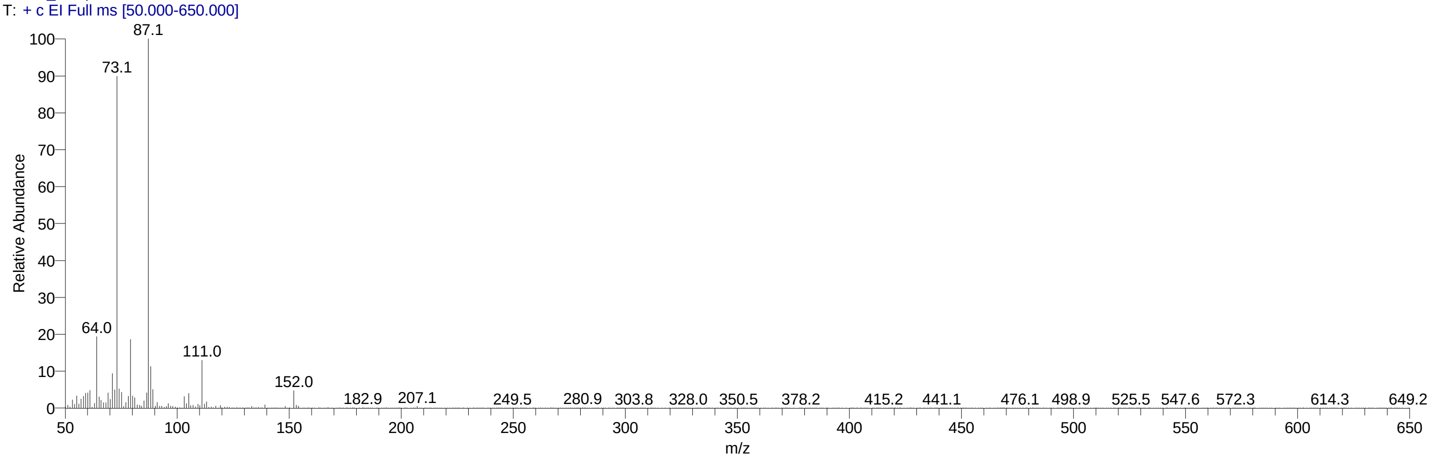

RT	Compound Name	Area %	Molecular Formula	Molecular Weight	Cas #	MF	Library
9.75	Trisulfide, methyl 2-propenyl	0.74	C4H8S3	152	34135-85-8	846	mainlib
9.75	Hexane, 3-methoxy-	0.74	C7H16O	116	54658-01-4	846	mainlib
9.75	3-HEXANOL, 2,4-DIMETHYL-	0.74	C8H18O	130	13432-25-2	654	WileyRegistry8e

My GC-MS Report

Compound Structure | Hit Spectrum

Trisulfide, methyl 2-propenyl
Formula C4H8S3, MW 152, CAS# 34135-85-8, Entry# 57366
1-Allyl-3-methyltrisulfane

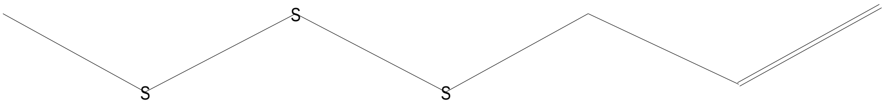

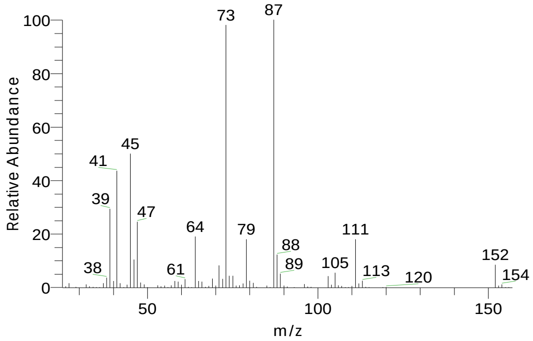

Hexane, 3-methoxy-
Formula C7H16O, MW 116, CAS# 54658-01-4, Entry# 17246
1-Ethylbutyl methyl ether #

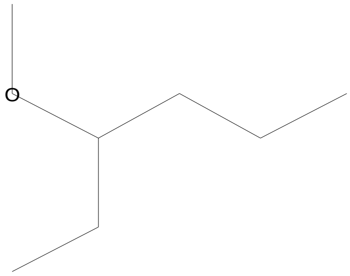

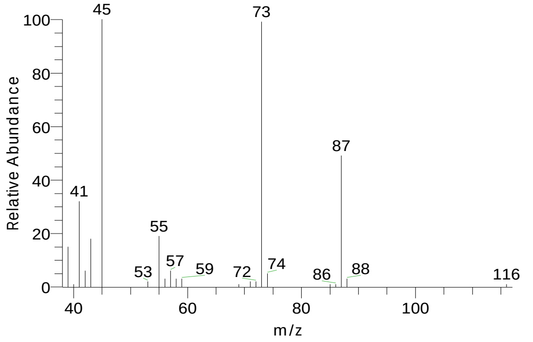

3-HEXANOL, 2,4-DIMETHYL-
Formula C8H18O, MW 130, CAS# 13432-25-2, Entry# 19686
2,4-DIMETHYLHEXAN-3-OL

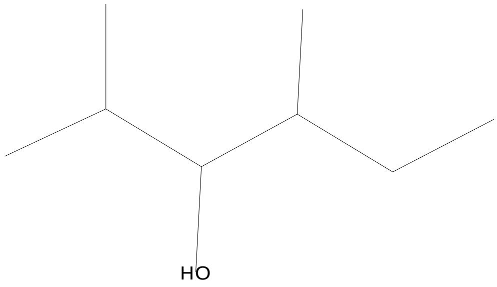

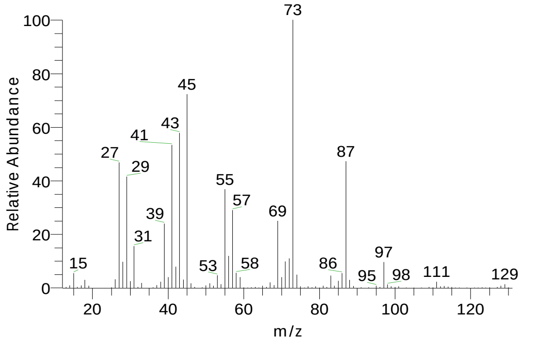

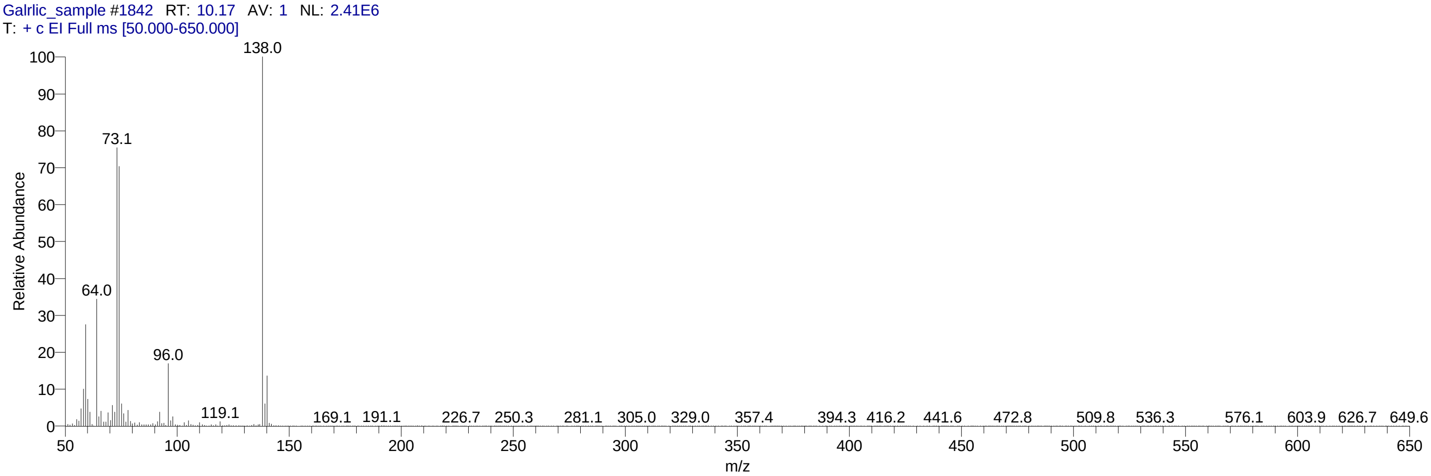

RT	Compound Name	Area %	Molecular Formula	Molecular Weight	Cas #	MF	Library
10.17	4-Methyl-1,2,3-trithiolane	1.58	C3H6S3	138	116664-29-0	920	mainlib
10.17	INDOLIZINE, OCTAHYDRO-8-METHYL-5-PENTYL-, [5R-(5à,8á,8Aá)]-	1.58	C14H27N	209	117959-79-2	988	WileyRegistry8e

My GC-MS Report

RT	Compound Name	Area %	Molecular Formula	Molecular Weight	Cas #	MF	Library
10.17	1,2,4-TRIAZINE, 3-(1-AZIRIDINYL)-, 1-OXIDE	1.58	C5H6N4O	138	65934-32-9	797	WileyRegistry8e

Compound Structure | Hit Spectrum

4-Methyl-1,2,3-trithiolane
Formula C3H6S3, MW 138, CAS# 116664-29-0, Entry# 124492
1,2,3-Trithiolane, 4-methyl-

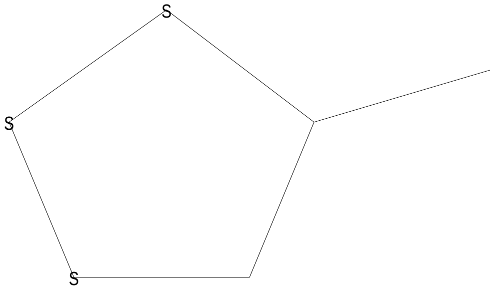

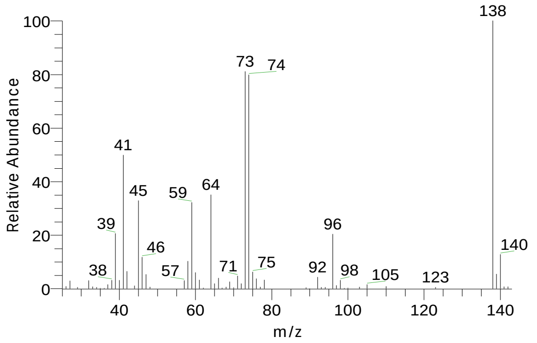

INDOLIZINE, OCTAHYDRO-8-METHYL-5-PENTYL-, [5R-(5à,8á,8Aá)]-
Formula C14H27N, MW 209, CAS# 117959-79-2, Entry# 94935
(-)-ALKALOID 209B FROM DENDROBATES

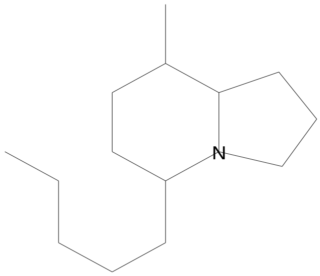

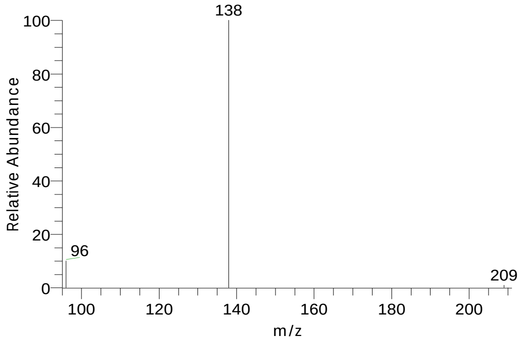

1,2,4-TRIAZINE, 3-(1-AZIRIDINYL)-, 1-OXIDE
Formula C5H6N4O, MW 138, CAS# 65934-32-9, Entry# 24054
3-AZIRIDINYL-1,2,4-TRIAZINE 1-OXIDE

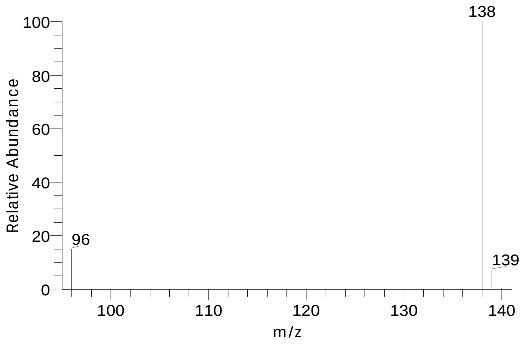

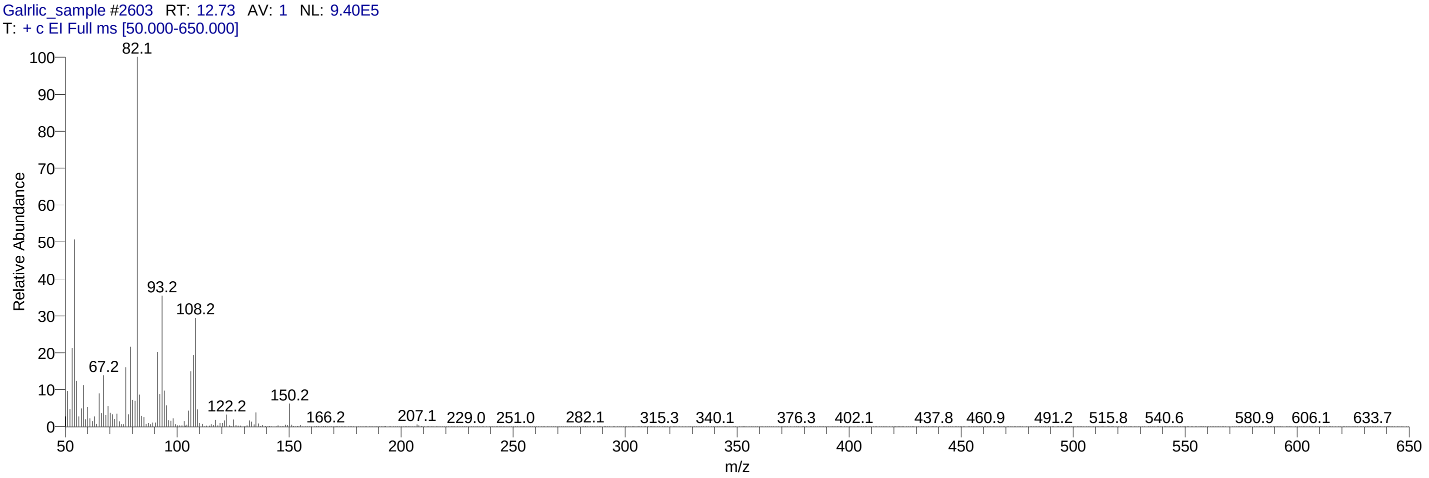

My GC-MS Report

RT	Compound Name	Area %	Molecular Formula	Molecular Weight	Cas #	MF	Library
12.73	(-)-Carvone	0.60	C10H14O	150	6485-40-1	921	mainlib
12.73	2-CYCLOHEXEN-1-ONE, 2-METHYL-5-(1-METHYLETHENYL)-	0.60	C10H14O	150	99-49-0	889	WileyRegistry8e
12.73	2-CYCLOHEXEN-1-ONE, 2-METHYL-5-(1-METHYLETHENYL)-, (S)-	0.60	C10H14O	150	2244-16-8	929	WileyRegistry8e

Compound Structure | Hit Spectrum

(-)-Carvone
Formula C10H14O, MW 150, CAS# 6485-40-1, Entry# 51136
2-Cyclohexen-1-one, 2-methyl-5-(1-methylethenyl)-, (R)-

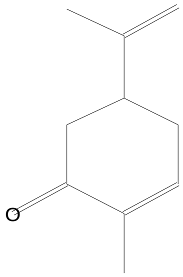

SI 859, RSI 921, mainlib, Entry# 51136, CAS# 6485-40-1, (-)-Carvone

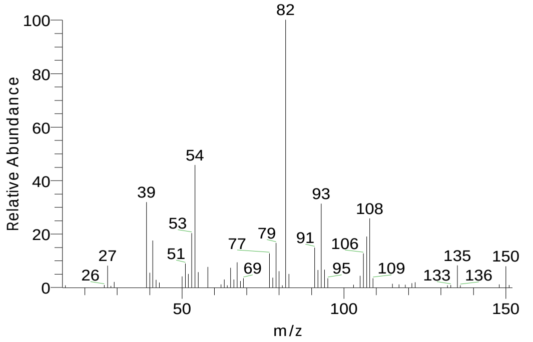

2-CYCLOHEXEN-1-ONE, 2-METHYL-5-(1-METHYLETHENYL)-
Formula C10H14O, MW 150, CAS# 99-49-0, Entry# 375319
5-ISOPROPENYL-2-METHYL-2-CYCLOHEXEN-1-ONE #

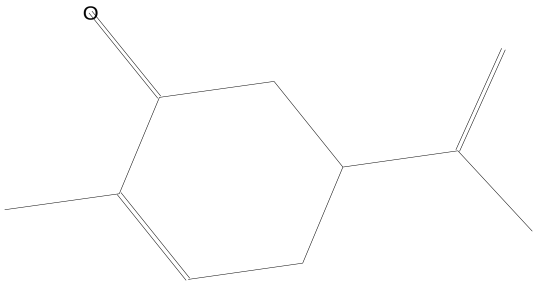

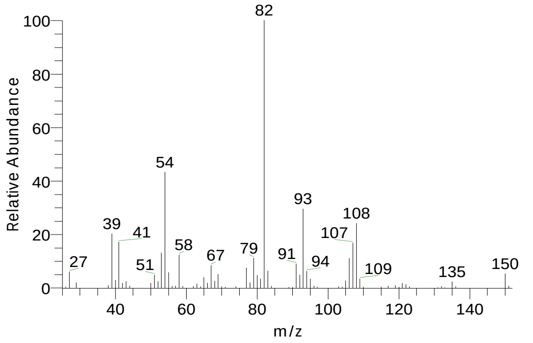

2-CYCLOHEXEN-1-ONE, 2-METHYL-5-(1-METHYLETHENYL)-, (S)-
Formula C10H14O, MW 150, CAS# 2244-16-8, Entry# 34019
5-ISOPROPENYL-2-METHYL-2-CYCLOHEXEN-1-ONE #

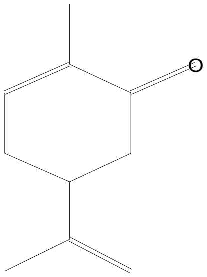

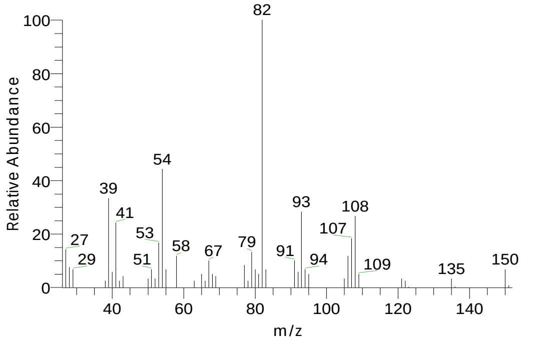

My GC-MS Report

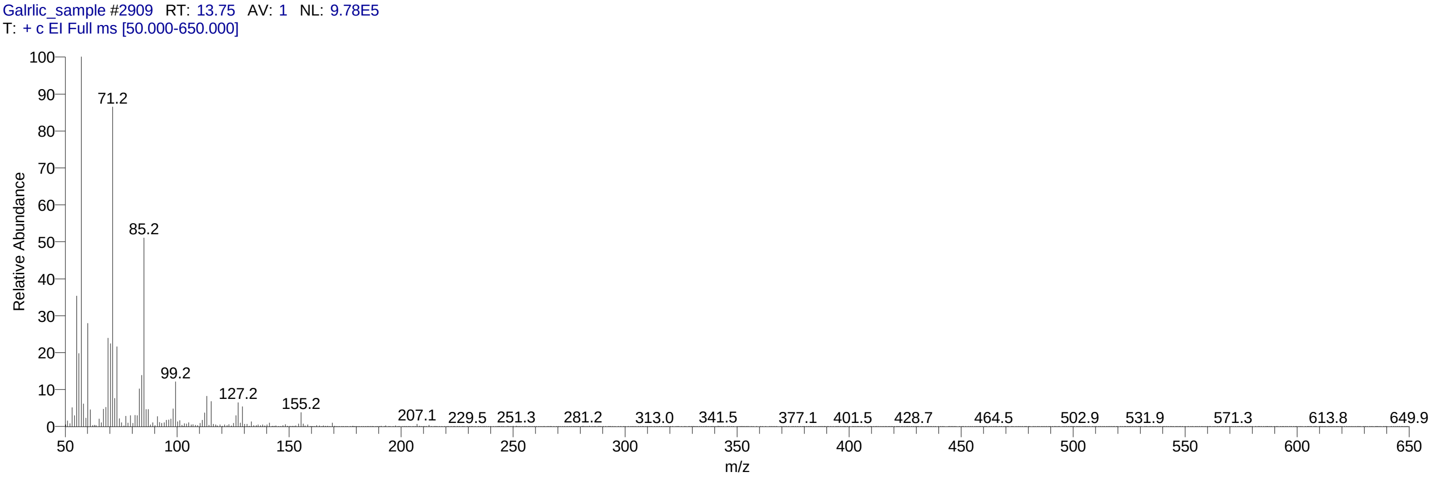

RT	Compound Name	Area %	Molecular Formula	Molecular Weight	Cas #	MF	Library
13.75	Dodecane, 2,6,11-trimethyl-	0.57	C15H32	212	31295-56-4	881	mainlib
13.75	PENTADECANE	0.57	C15H32	212	629-62-9	909	WileyRegistry8e
13.75	HEXADECANE	0.57	C16H34	226	544-76-3	857	WileyRegistry8e

Compound Structure	Hit Spectrum

Dodecane, 2,6,11-trimethyl-
Formula C15H32, MW 212, CAS# 31295-56-4, Entry# 25343
2,6,11-Trimethyldodecane

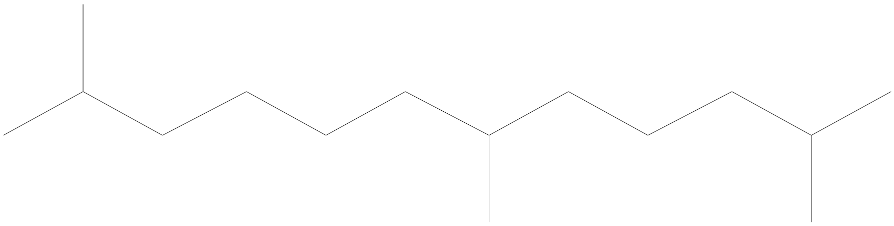

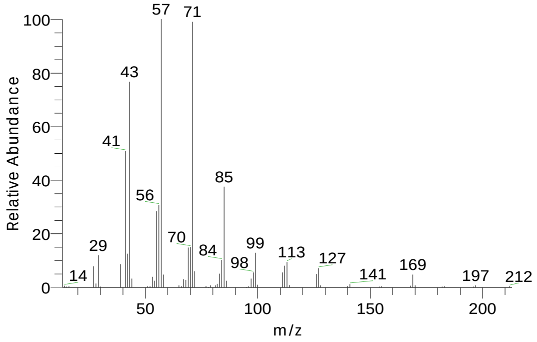

PENTADECANE
Formula C15H32, MW 212, CAS# 629-62-9, Entry# 98827
BRN 1698194

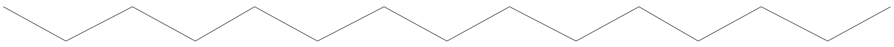

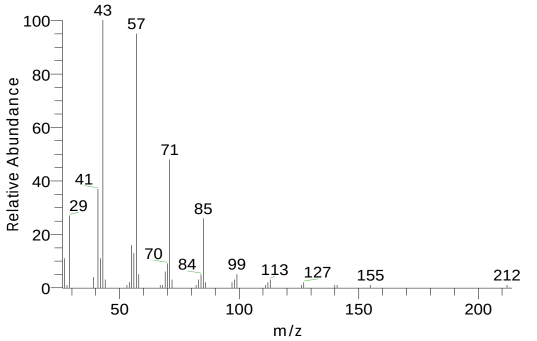

HEXADECANE
Formula C16H34, MW 226, CAS# 544-76-3, Entry# 114829
AI3-06522

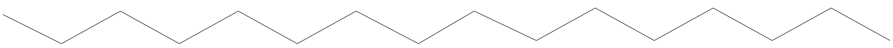

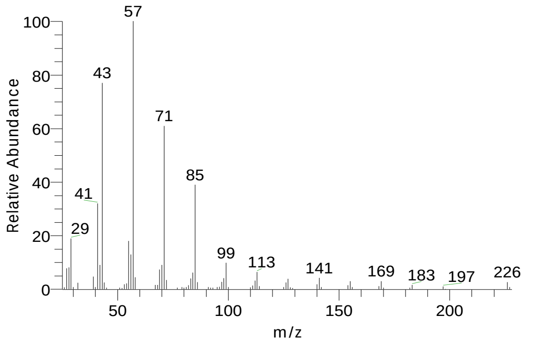

My GC-MS Report

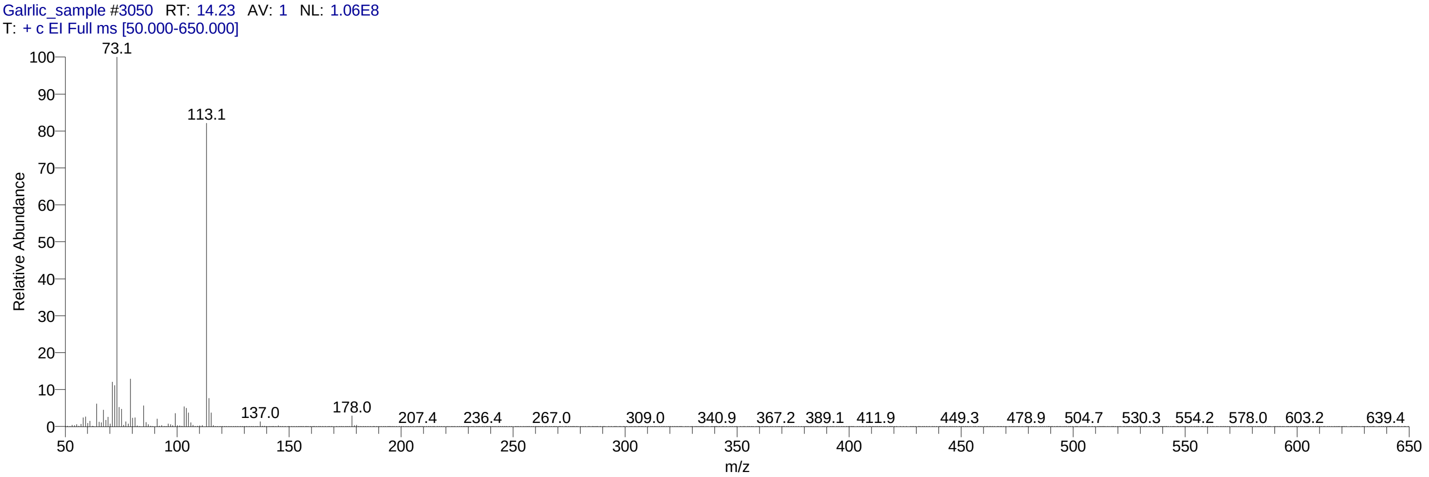

RT	Compound Name	Area %	Molecular Formula	Molecular Weight	Cas #	MF	Library
14.23	Trisulfide, di-2-propenyl	38.21	C6H10S3	178	2050-87-5	930	replib
14.23	Trisulfide, di-2-propenyl	38.21	C6H10S3	178	2050-87-5	923	mainlib
14.23	Trisulfide, di-2-propenyl	38.21	C6H10S3	178	2050-87-5	838	replib

Compound Structure | Hit Spectrum

Trisulfide, di-2-propenyl
Formula C6H10S3, MW 178, CAS# 2050-87-5, Entry# 9825
Allyl trisulfide

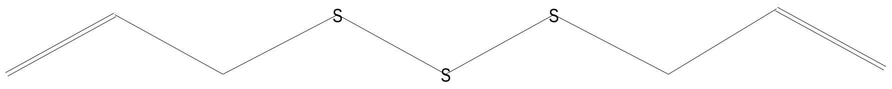

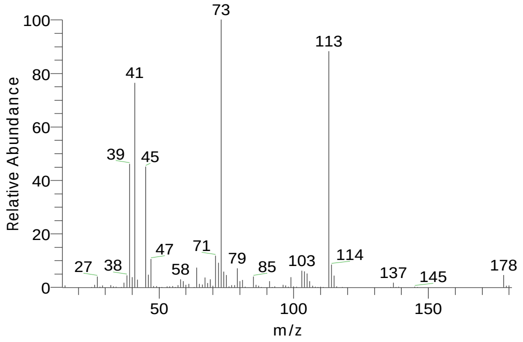

Trisulfide, di-2-propenyl
Formula C6H10S3, MW 178, CAS# 2050-87-5, Entry# 40554
Allyl trisulfide

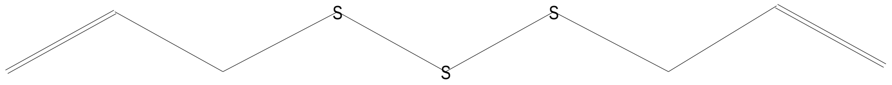

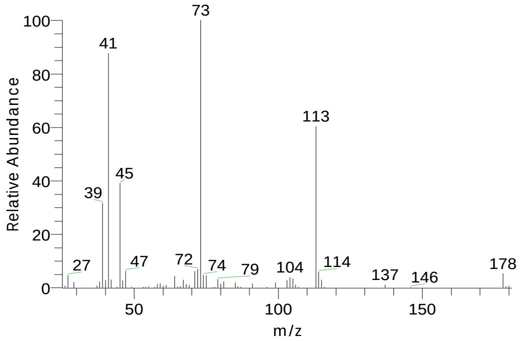

Trisulfide, di-2-propenyl
Formula C6H10S3, MW 178, CAS# 2050-87-5, Entry# 9824
Allyl trisulfide

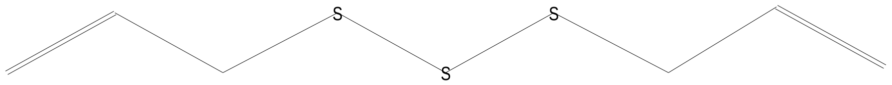

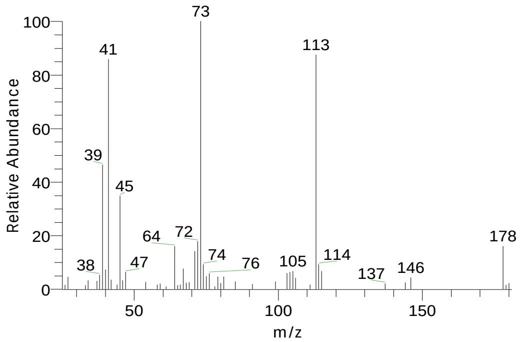

My GC-MS Report

Galrlic_sample #3507 RT: 15.76 AV: 1 NL: 3.04E6
T: + c EI Full ms [50.000-650.000]

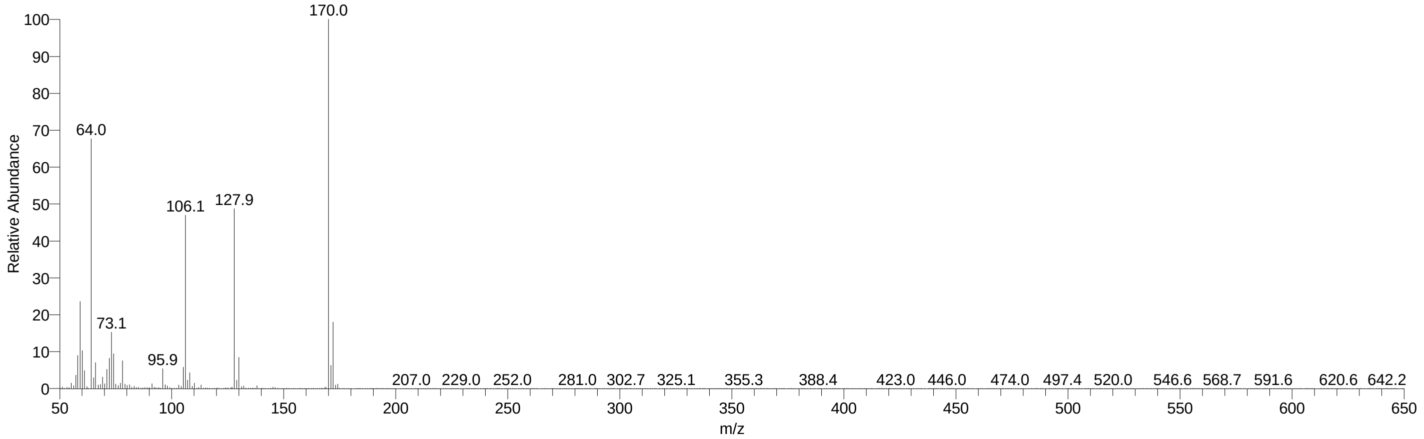

RT	Compound Name	Area %	Molecular Formula	Molecular Weight	Cas #	MF	Library
15.76	THIOPHENE-2,3-DICARBOXYLIC ACID	1.41	C6H4O4S	172	1451-95-2	923	WileyRegistry8e
15.76	5-Methyl-1,2,3,4-tetrathiane	1.41	C3H6S4	170	116664-30-3	916	mainlib
15.76	Propachlor	1.41	C11H14ClNO	211	1918-16-7	899	nist_msms

Compound Structure	Hit Spectrum

THIOPHENE-2,3-DICARBOXYLIC ACID
Formula C6H4O4S, MW 172, CAS# 1451-95-2, Entry# 54791
2,3-DICARBOXYTHIOPHENE

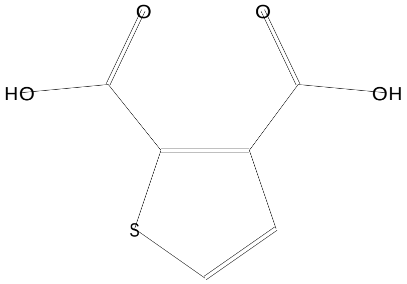

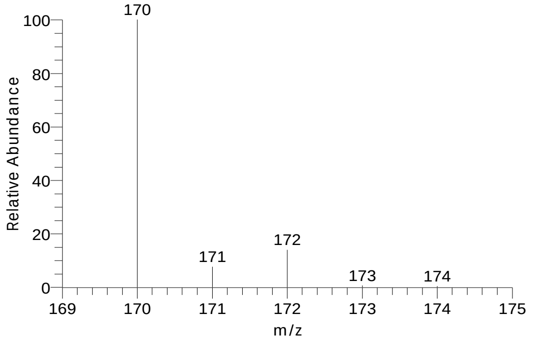

5-Methyl-1,2,3,4-tetrathiane
Formula C3H6S4, MW 170, CAS# 116664-30-3, Entry# 158633
1,2,3,4-Tetrathiane, 5-methyl-

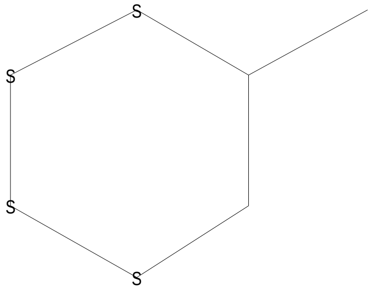

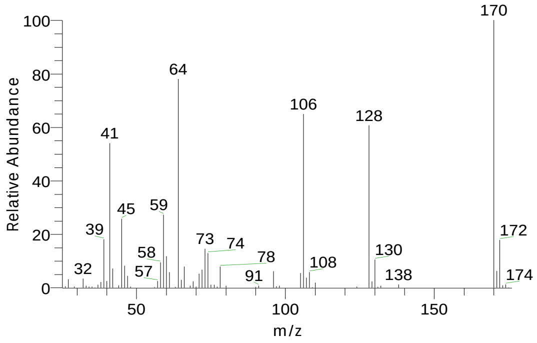

SI 888, RSI 899, nist_msms, Entry# 112342, CAS# 1918-16-7, Propachlor

Propachlor
Formula C11H14ClNO, MW 211, CAS# 1918-16-7, Entry# 112342
$:03[M+H]+

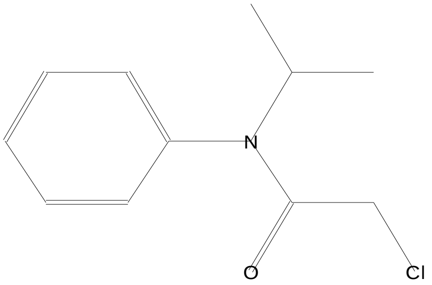

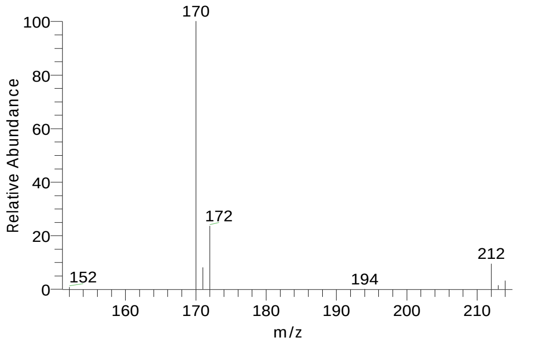

My GC-MS Report

Galrlic_sample #3676 RT: 16.32 AV: 1 NL: 2.01E6
T: + c EI Full ms [50.000-650.000]

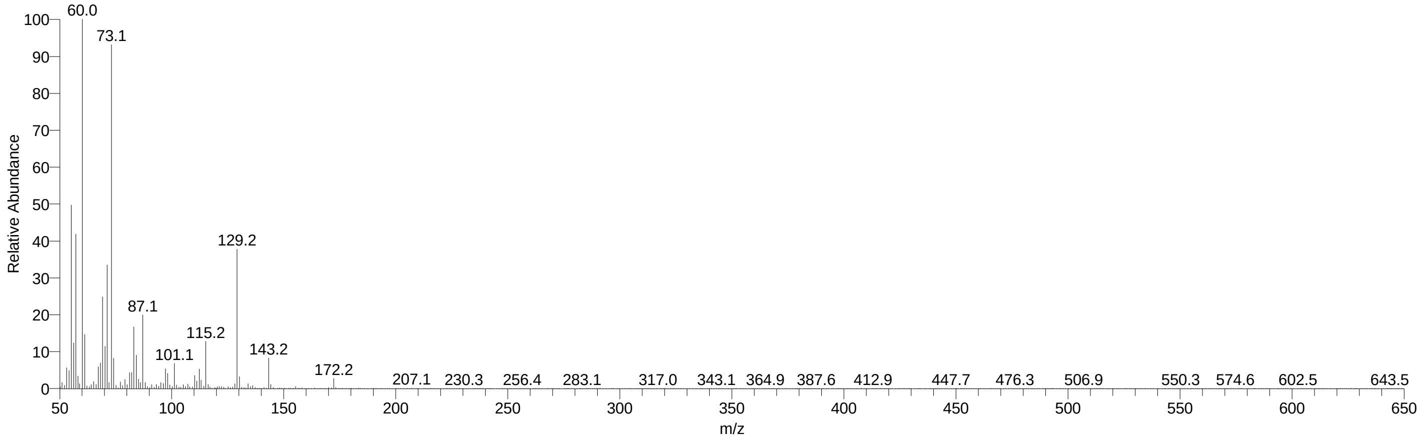

RT	Compound Name	Area %	Molecular Formula	Molecular Weight	Cas #	MF	Library
16.32	n-Decanoic acid	1.89	C10H20O2	172	334-48-5	931	replib
16.32	DECANOIC ACID	1.89	C10H20O2	172	334-48-5	915	WileyRegistry8e
16.32	n-Decanoic acid	1.89	C10H20O2	172	334-48-5	914	replib

Compound Structure	Hit Spectrum

n-Decanoic acid
Formula C10H20O2, MW 172, CAS# 334-48-5, Entry# 9640
Decanoic acid

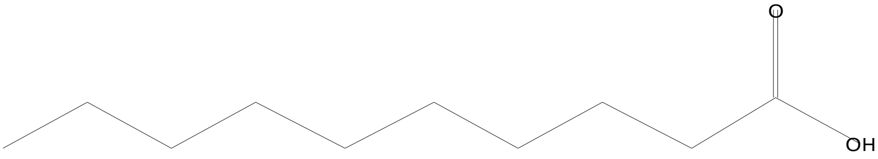

SI 906, RSI 931, replib, Entry# 9640, CAS# 334-48-5, n-Decanoic acid

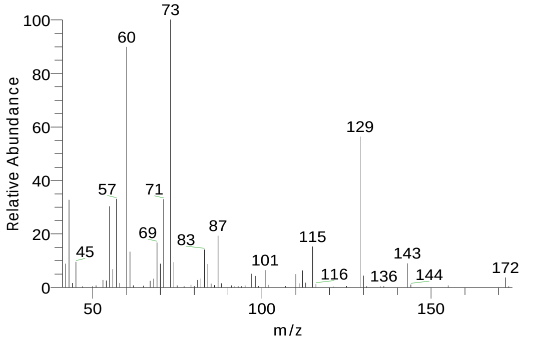

DECANOIC ACID
Formula C10H20O2, MW 172, CAS# 334-48-5, Entry# 396755
CAPRIC ACID

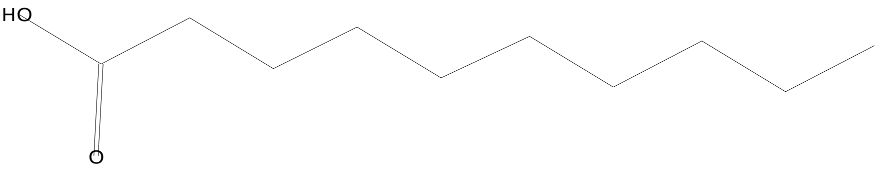

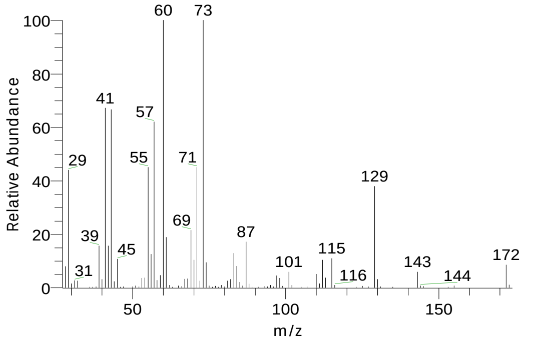

n-Decanoic acid
Formula C10H20O2, MW 172, CAS# 334-48-5, Entry# 7548
Decanoic acid

SI 898, RSI 914, replib, Entry# 7548, CAS# 334-48-5, n-Decanoic acid

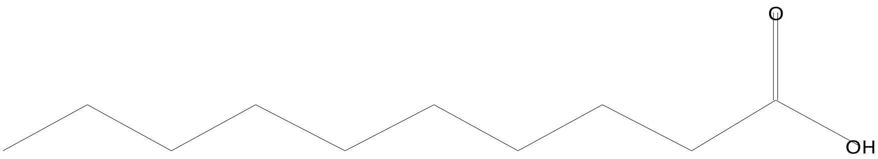

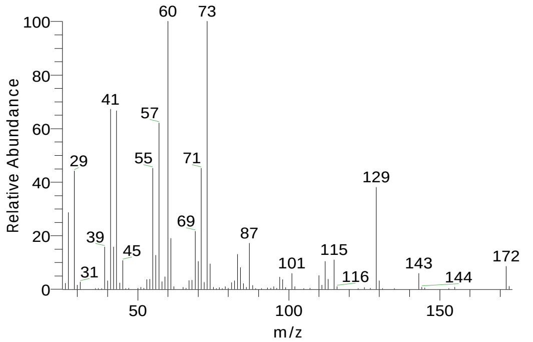

My GC-MS Report

Galrlic_sample #4449 RT: 18.92 AV: 1 NL: 5.03E5
T: + c EI Full ms [50.000-650.000]

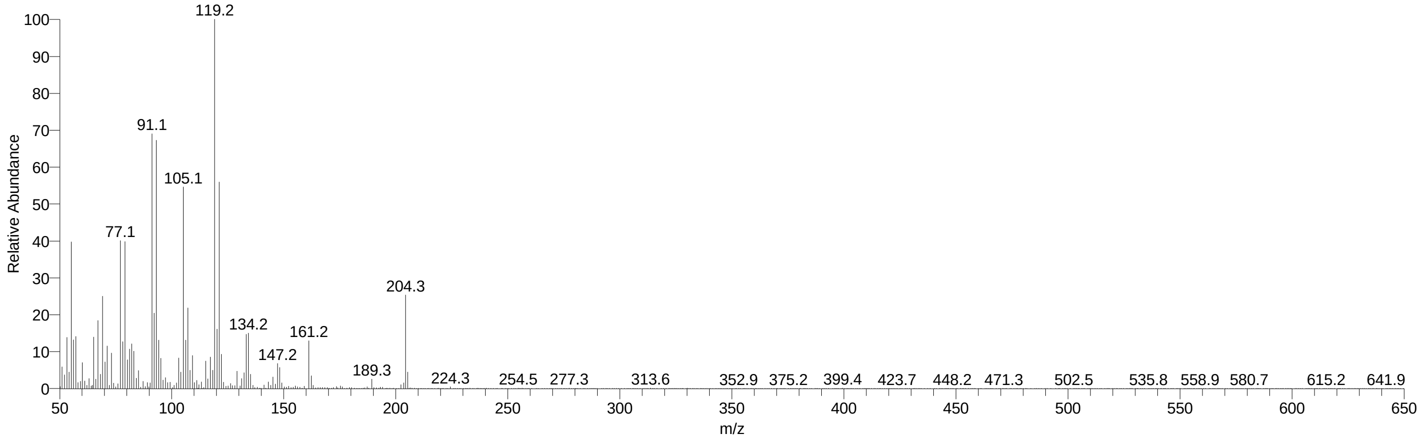

RT	Compound Name	Area %	Molecular Formula	Molecular Weight	Cas #	MF	Library
18.92	(R)-1-Methyl-4-(6-methylhept-5-en-2-yl)cyclohexa-1,4-diene	0.32	C15H24	204	28976-67-2	892	mainlib
18.92	ç-CURCUMENE	0.32	C15H24	204	NA	929	WileyRegistry8e
18.92	Tricyclo[5.4.0.0(2,8)]undec-9-ene, 2,6,6,9-tetramethyl-, (1R,2S,7R,8R)-	0.32	C15H24	204	5989-08-2	878	replib

Compound Structure	Hit Spectrum

(R)-1-Methyl-4-(6-methylhept-5-en-2-yl)cyclohexa-1,4-diene
Formula C15H24, MW 204, CAS# 28976-67-2, Entry# 99924
á-Curcumene

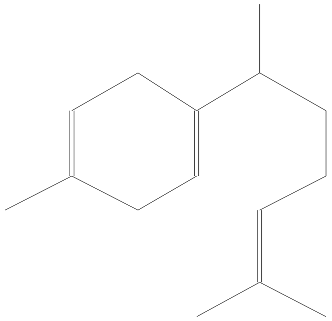

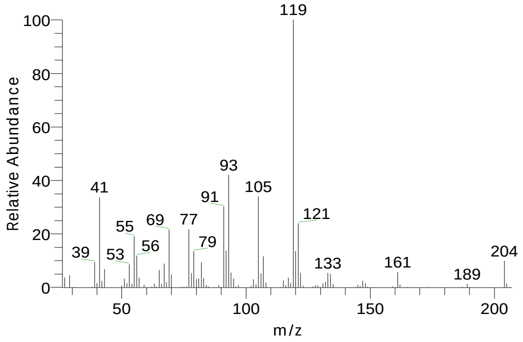

ç-CURCUMENE
Formula C15H24, MW 204, CAS# NA, Entry# 89532

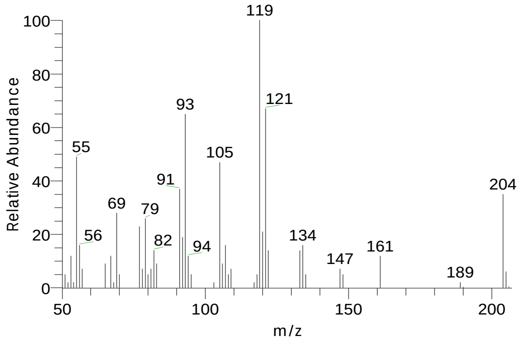

Tricyclo[5.4.0.0(2,8)]undec-9-ene, 2,6,6,9-tetramethyl-, (1R,2S,7R,8R)-
Formula C15H24, MW 204, CAS# 5989-08-2, Entry# 18510
à-Longipinene

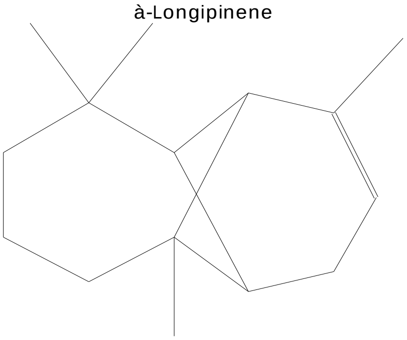

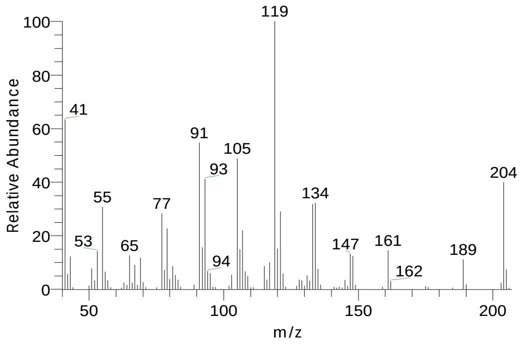

My GC-MS Report

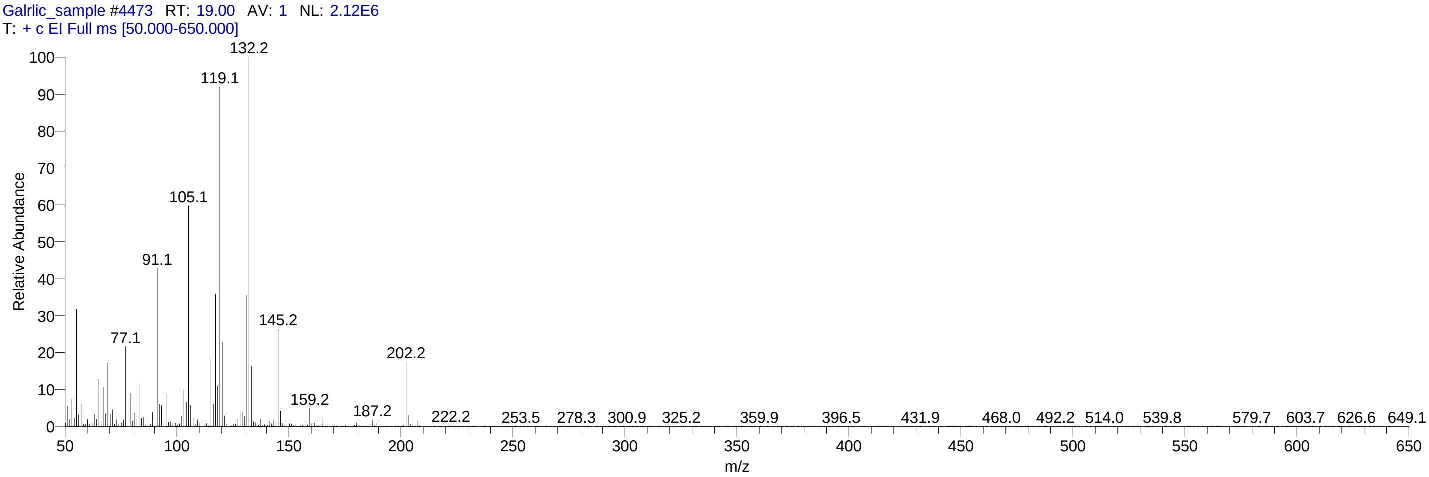

RT	Compound Name	Area %	Molecular Formula	Molecular Weight	Cas #	MF	Library
19.00	Benzene, 1-(1,5-dimethyl-4-hexenyl)-4-methyl-	2.06	C15H22	202	644-30-4	958	replib
19.00	BENZENE, 1-(1,5-DIMETHYL-4-HEXENYL)-4-METHYL-	2.06	C15H22	202	644-30-4	967	WileyRegistry8e
19.00	BENZENE, 1-(1,5-DIMETHYL-4-HEXENYL)-4-METHYL-	2.06	C15H22	202	644-30-4	902	WileyRegistry8e

Compound Structure	Hit Spectrum

Benzene, 1-(1,5-dimethyl-4-hexenyl)-4-methyl-
Formula C15H22, MW 202, CAS# 644-30-4, Entry# 21026
à-Curcumene

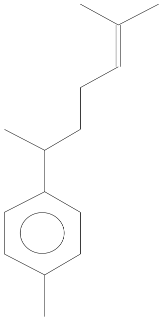

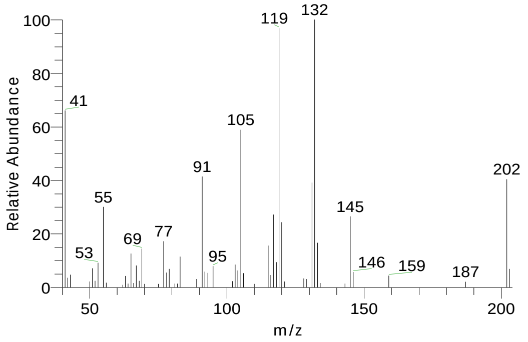

BENZENE, 1-(1,5-DIMETHYL-4-HEXENYL)-4-METHYL-
Formula C15H22, MW 202, CAS# 644-30-4, Entry# 86966
1-(1,5-DIMETHYL-4-HEXENYL)-4-METHYLBENZENE #

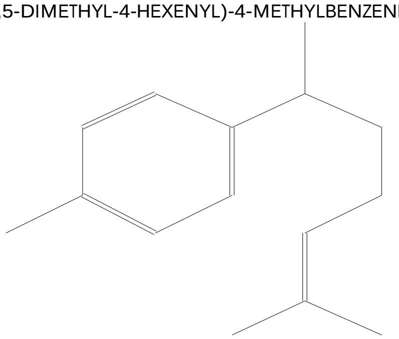

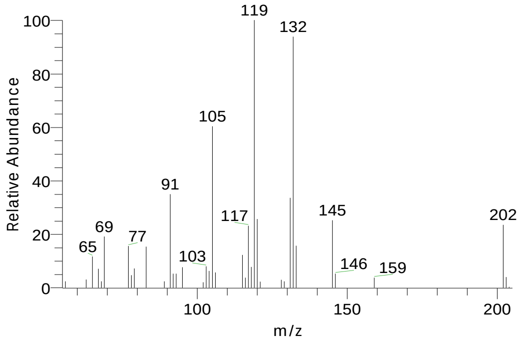

My GC-MS Report

Compound Structure | Hit Spectrum

BENZENE, 1-(1,5-DIMETHYL-4-HEXENYL)-4-METHYL-
Formula C15H22, MW 202, CAS# 644-30-4, Entry# 86963
1-(1,5-DIMETHYL-4-HEXENYL)-4-METHYLBENZENE #

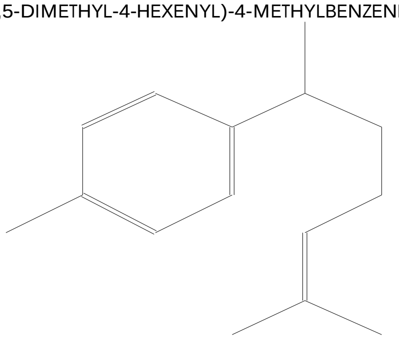

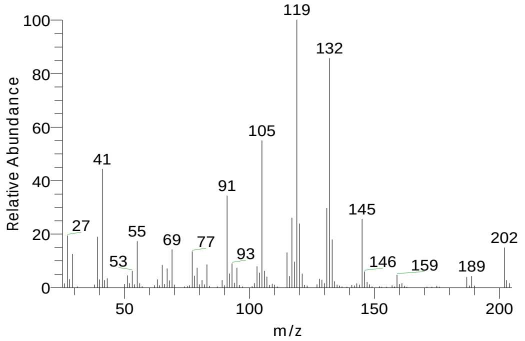

Galrlic_sample #4540 RT: 19.22 AV: 1 NL: 2.73E6
T: + c EI Full ms [50.000-650.000]

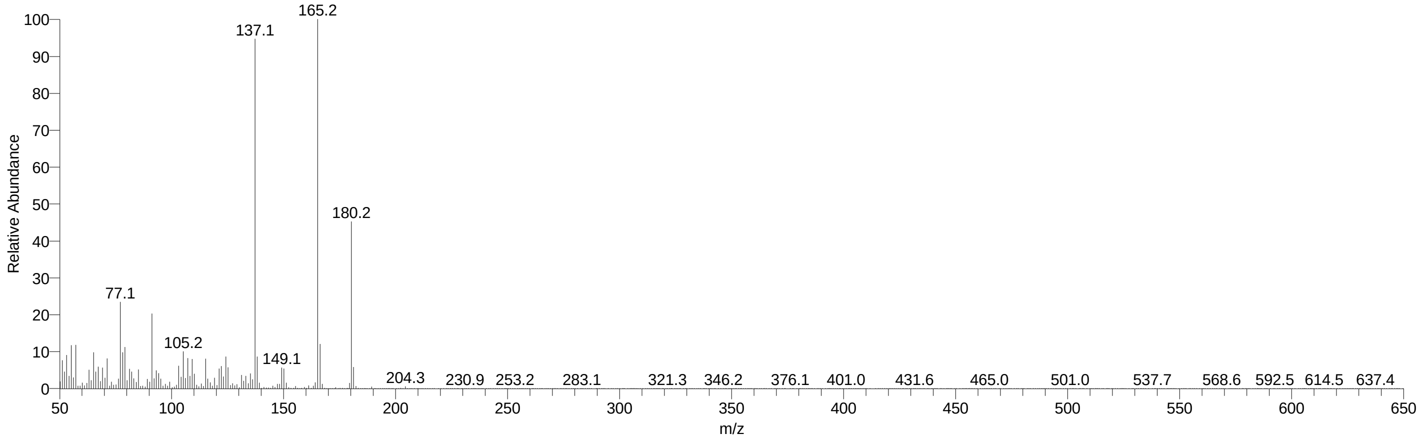

RT	Compound Name	Area %	Molecular Formula	Molecular Weight	Cas #	MF	Library
19.22	3-tert-Butyl-4-hydroxyanisole	1.67	C11H16O2	180	121-00-6	907	mainlib
19.22	BUTYL HYDROXY ANISOLE	1.67	C11H16O2	180	NA	907	WileyRegistry8e
19.22	3-tert-Butyl-4-hydroxyanisole	1.67	C11H16O2	180	121-00-6	893	replib

Compound Structure | Hit Spectrum

3-tert-Butyl-4-hydroxyanisole
Formula C11H16O2, MW 180, CAS# 121-00-6, Entry# 154243
2-tert-Butyl-4-methoxyphenol

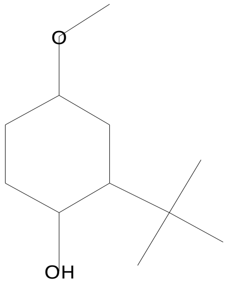

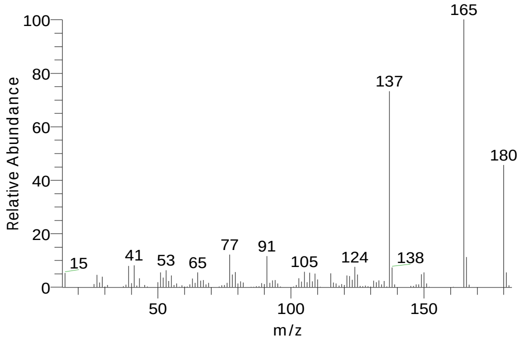

My GC-MS Report

Compound Structure | Hit Spectrum

BUTYL HYDROXY ANISOLE
Formula C11H16O2, MW 180, CAS# NA, Entry# 63103

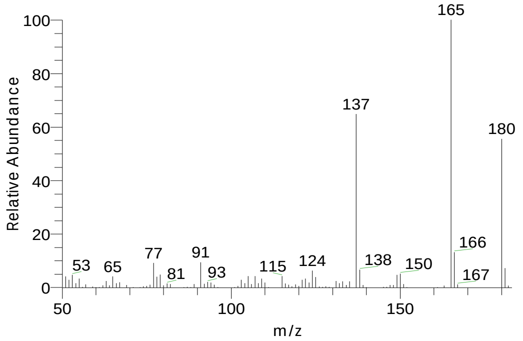

3-tert-Butyl-4-hydroxyanisole
Formula C11H16O2, MW 180, CAS# 121-00-6, Entry# 25424
2-tert-Butyl-4-methoxyphenol

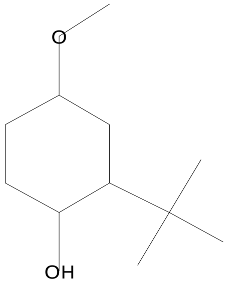

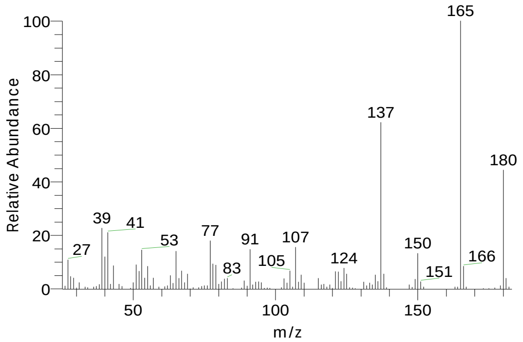

Galrlic_sample #4575 RT: 19.34 AV: 1 NL: 9.53E5
T: + c EI Full ms [50.000-650.000]

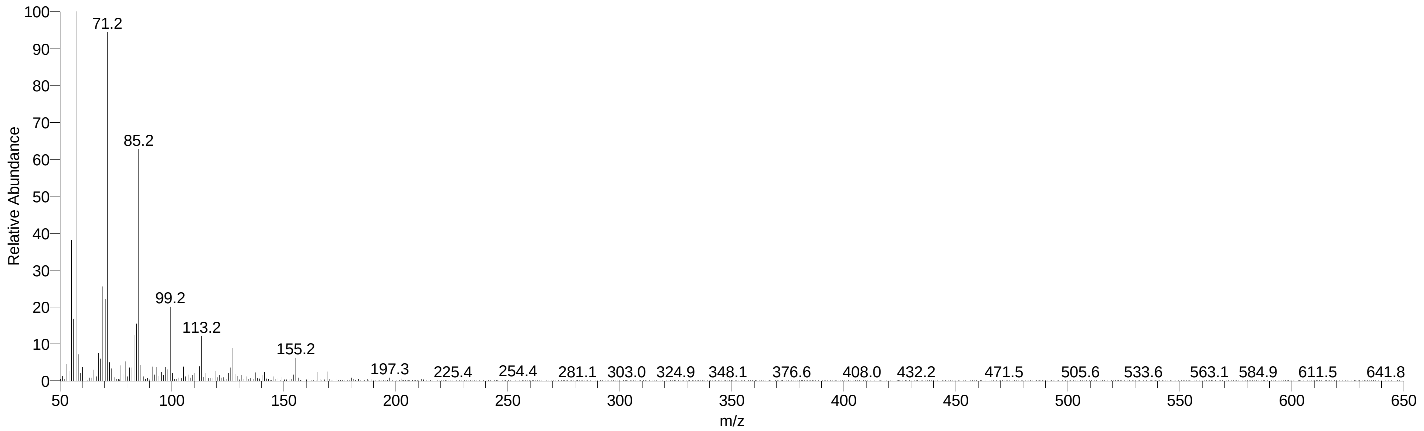

RT	Compound Name	Area %	Molecular Formula	Molecular Weight	Cas #	MF	Library
19.34	Tetradecane, 2,6,10-trimethyl-	0.40	C17H36	240	14905-56-7	847	mainlib
19.34	Heptadecane, 2,6,10,15-tetramethyl-	0.40	C21H44	296	54833-48-6	849	mainlib
19.34	HEPTADECANE, 2,6,10,15-TETRAMETHYL-	0.40	C21H44	296	54833-48-6	849	WileyRegistry8e

My GC-MS Report

Compound Structure | Hit Spectrum

Tetradecane, 2,6,10-trimethyl-
Formula C17H36, MW 240, CAS# 14905-56-7, Entry# 24375
2,6,10-Trimethyltetradecane

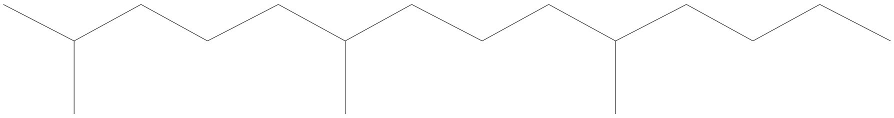

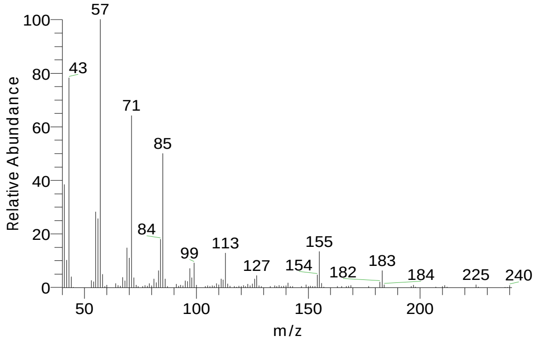

Heptadecane, 2,6,10,15-tetramethyl-
Formula C21H44, MW 296, CAS# 54833-48-6, Entry# 25344
2,6,10,15-Tetramethylheptadecane #

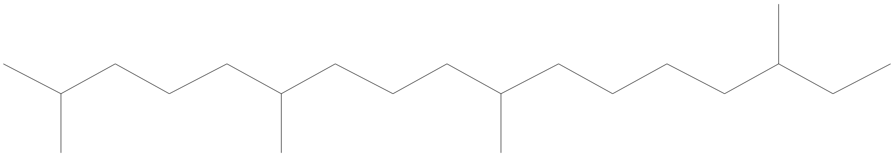

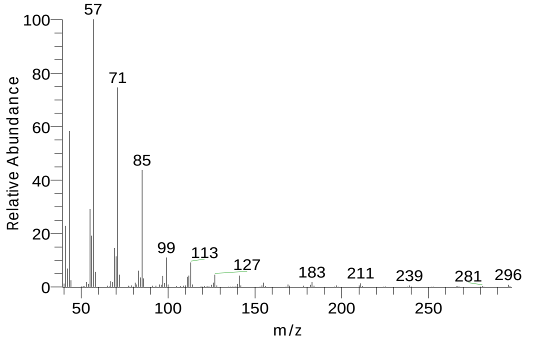

HEPTADECANE, 2,6,10,15-TETRAMETHYL-
Formula C21H44, MW 296, CAS# 54833-48-6, Entry# 186364
2,6,10,15-TETRAMETHYLHEPTADECANE

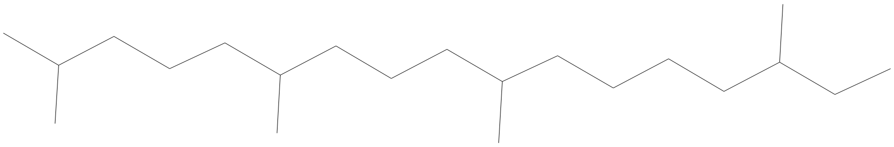

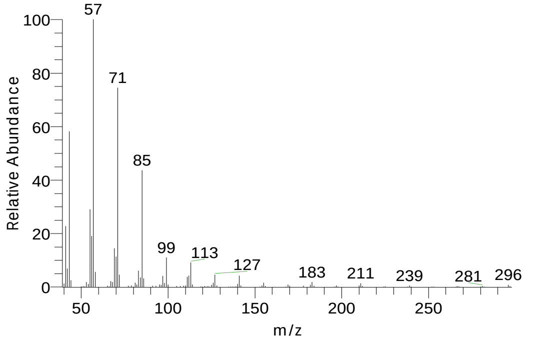

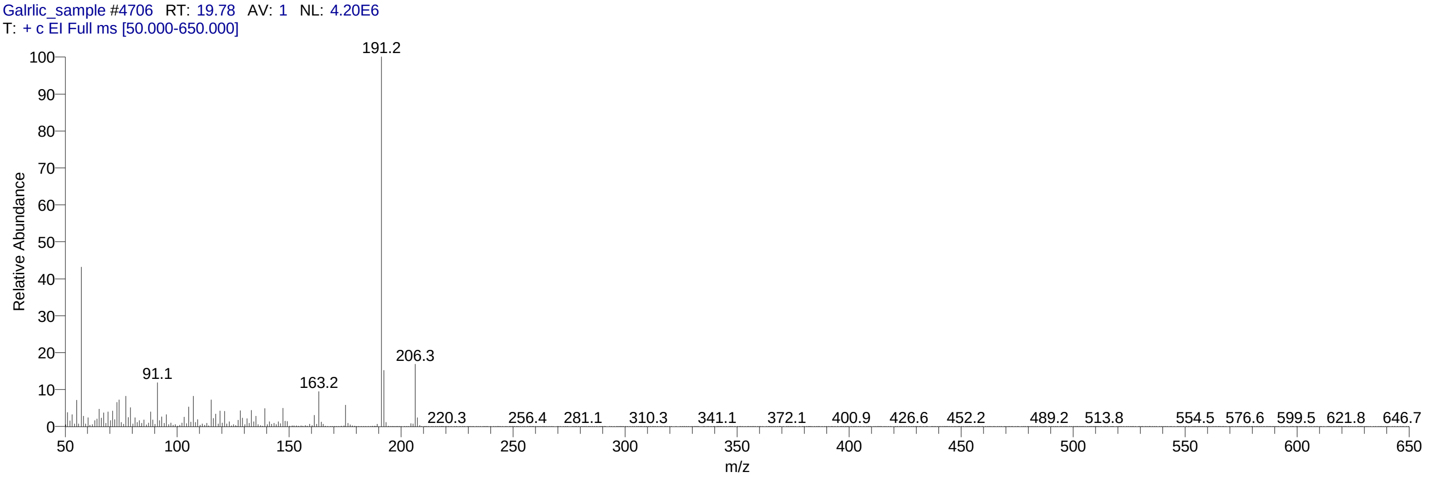

RT	Compound Name	Area %	Molecular Formula	Molecular Weight	Cas #	MF	Library
19.78	1,4-BENZENEDIOL, 2-(1,1-DIMETHYLETHYL)-5-(2-PROPENYL)-	1.75	C13H18O2	206	73685-60-6	964	WileyRegistry8e
19.78	15-METHYLTRICYCLO[6.5.2(13,14).0(7,15)]PENTADECA-1,3,5,7,9,11,13-HEPTENE	1.75	C16H14	206	102521-04-0	953	WileyRegistry8e

My GC-MS Report

RT	Compound Name	Area %	Molecular Formula	Molecular Weight	Cas #	MF	Library
19.78	3,4-DIHYDRO-2H-1,5-(3"-T-BUTYL)BENZO DIOXEPINE	1.75	C13H18O2	206	NA	952	WileyRegistry8e

Compound Structure | Hit Spectrum

1,4-BENZENEDIOL, 2-(1,1-DIMETHYLETHYL)-5-(2-PROPENYL)-
Formula C13H18O2, MW 206, CAS# 73685-60-6, Entry# 91211
2-ALLYL-5-T-BUTYLHYDROQUINONE

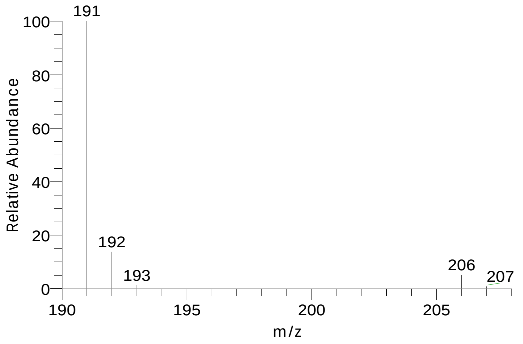

15-METHYLTRICYCLO[6.5.2(13,14).0(7,15)]PENTADECA-1,3,5,7,9,11,13-HEPTENE
Formula C16H14, MW 206, CAS# 102521-04-0, Entry# 92034

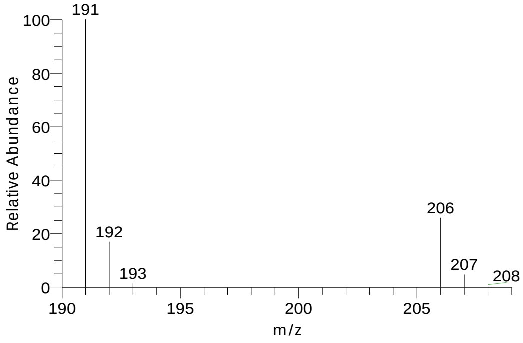

3,4-DIHYDRO-2H-1,5-(3"-T-BUTYL)BENZODIOXEPINE
Formula C13H18O2, MW 206, CAS# NA, Entry# 91434

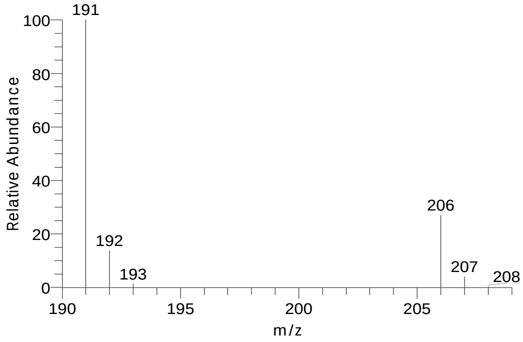

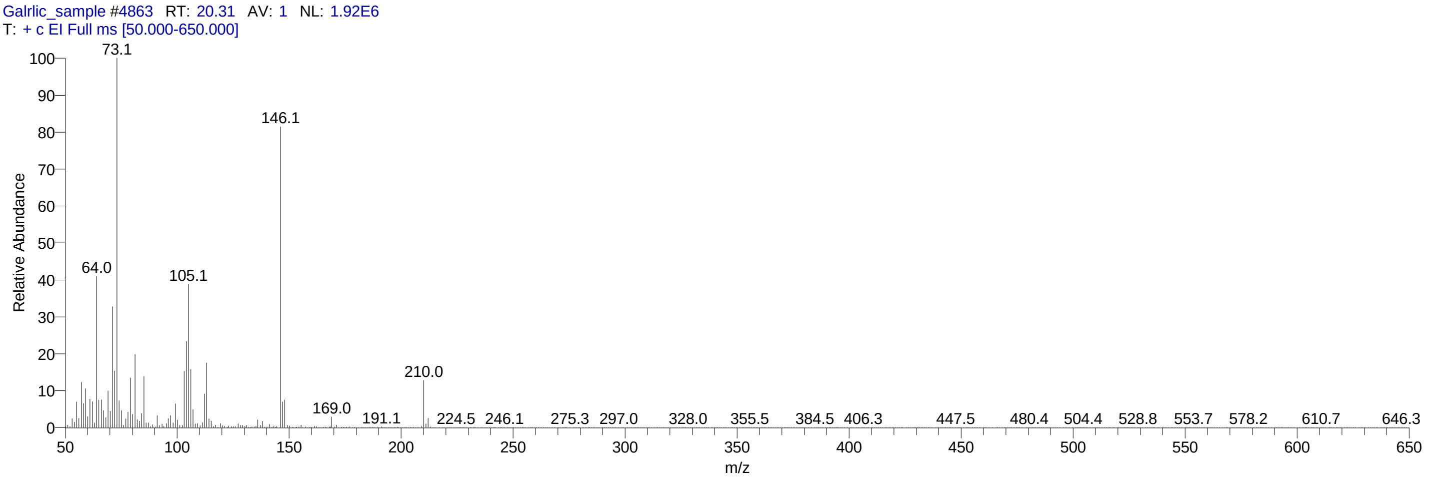

My GC-MS Report

RT	Compound Name	Area %	Molecular Formula	Molecular Weight	Cas #	MF	Library
20.31	ALLYL TETRASULFIDE	1.15	C6H10S4	210	2444-49-7	913	WileyRegistry8e
20.31	Tetrasulfide, di-2-propenyl	1.15	C6H10S4	210	2444-49-7	912	mainlib
20.31	(E)-1-Allyl-2-(prop-1-en-1-yl)disulfane	1.15	C6H10S2	146	122156-02-9	788	mainlib

Compound Structure

Hit Spectrum

ALLYL TETRASULFIDE
Formula C6H10S4, MW 210, CAS# 2444-49-7, Entry# 95039
TETRASULFIDE, DIALLYL

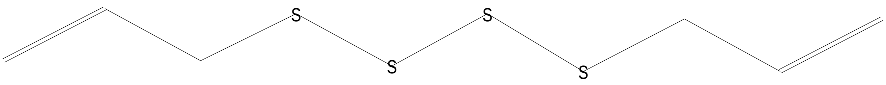

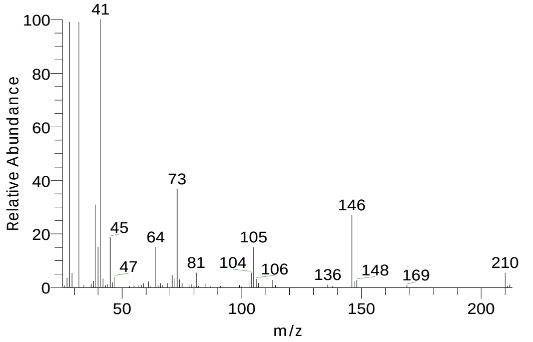

Tetrasulfide, di-2-propenyl
Formula C6H10S4, MW 210, CAS# 2444-49-7, Entry# 3563
Diallyl tetrasulphide

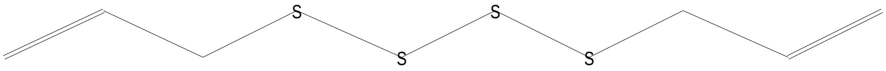

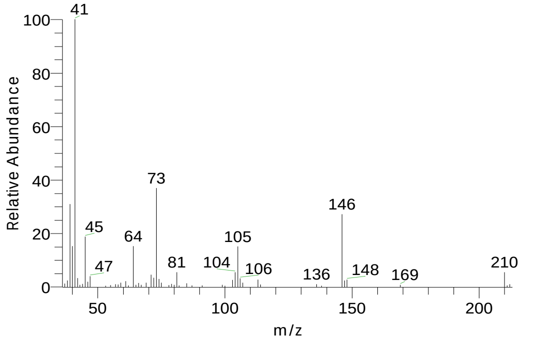

(E)-1-Allyl-2-(prop-1-en-1-yl)disulfane
Formula C6H10S2, MW 146, CAS# 122156-02-9, Entry# 2594
Disulfide, (1E)-1-propen-1-yl 2-propen-1-yl

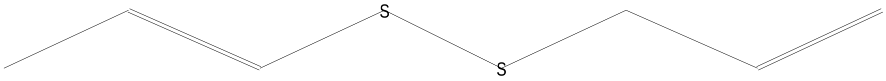

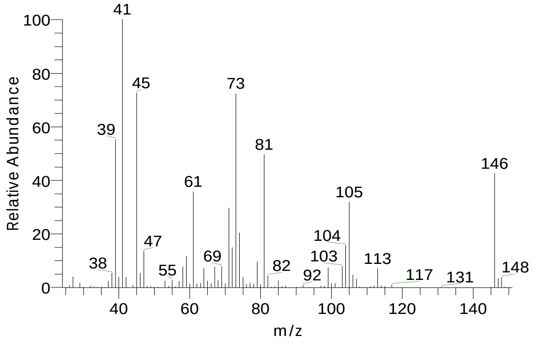

My GC-MS Report

Galrlic_sample #5318 RT: 21.83 AV: 1 NL: 1.30E6
T: + c EI Full ms [50.000-650.000]

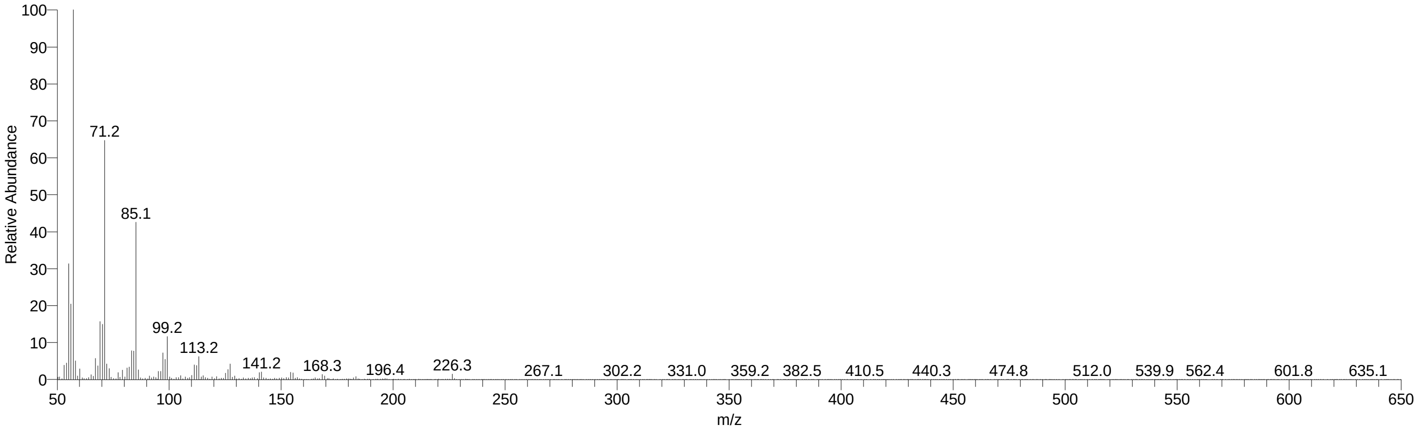

RT	Compound Name	Area %	Molecular Formula	Molecular Weight	Cas #	MF	Library
21.83	Nonadecane	0.58	C19H40	268	629-92-5	947	replib
21.83	Hexadecane	0.58	C16H34	226	544-76-3	915	replib
21.83	HEXADECANE	0.58	C16H34	226	544-76-3	847	WileyRegistry8e

Compound Structure	Hit Spectrum

Nonadecane
Formula C19H40, MW 268, CAS# 629-92-5, Entry# 6141
n-Nonadecane

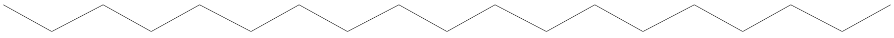

SI 867, RSI 947, replib, Entry# 6141, CAS# 629-92-5, Nonadecane

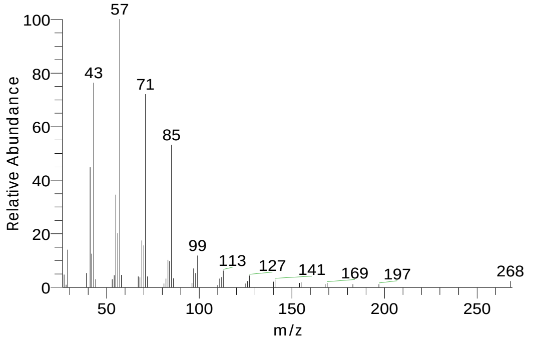

Hexadecane
Formula C16H34, MW 226, CAS# 544-76-3, Entry# 6168
n-Cetane

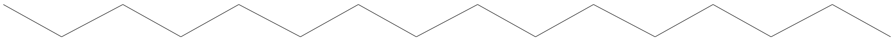

SI 856, RSI 915, replib, Entry# 6168, CAS# 544-76-3, Hexadecane

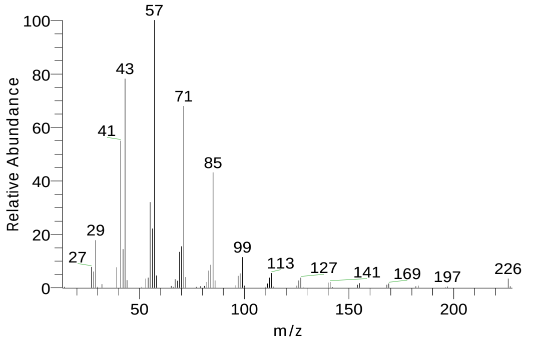

HEXADECANE
Formula C16H34, MW 226, CAS# 544-76-3, Entry# 114828
AI3-06522

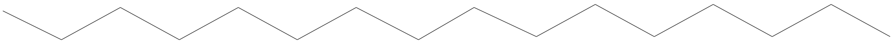

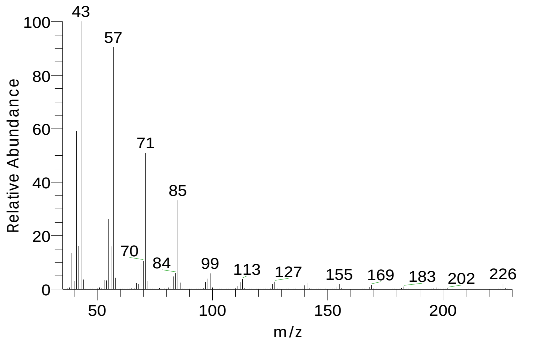

My GC-MS Report

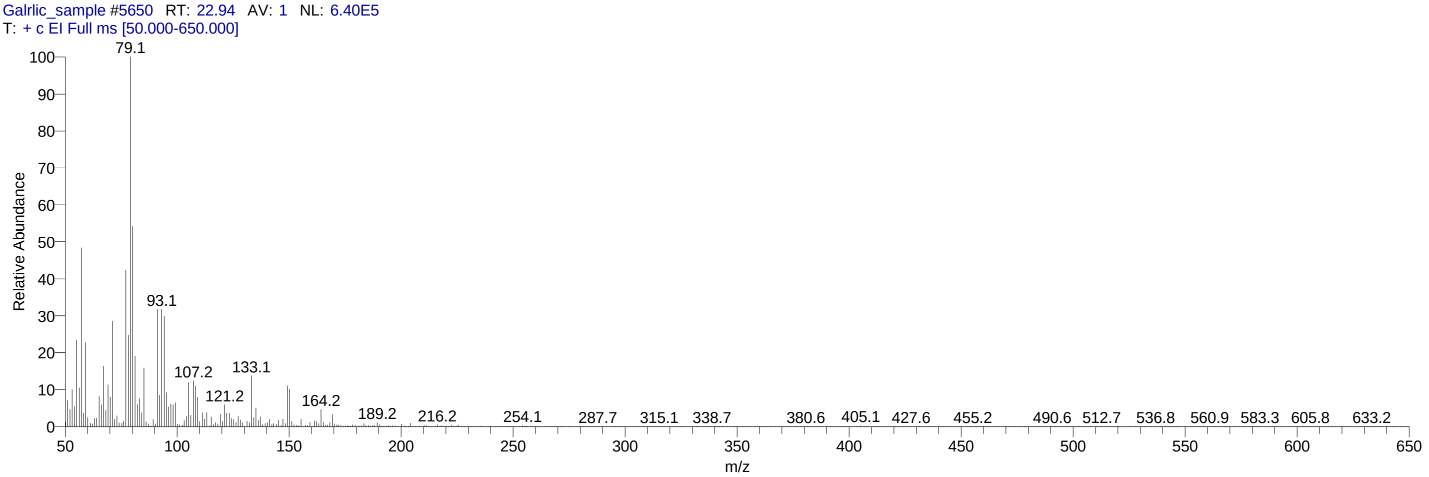

RT	Compound Name	Area %	Molecular Formula	Molecular Weight	Cas #	MF	Library
22.94	10-Heptadecen-8-ynoic acid, methyl ester, (E)-	0.67	C18H30O2	278	16714-85-5	801	mainlib
22.94	10-HEPTADECEN-8-YNOIC ACID, METHYL ESTER, (E)-	0.67	C18H30O2	278	16714-85-5	801	WileyRegistry8e
22.94	Z,Z,Z-1,4,6,9-Nonadecatetraene	0.67	C19H32	260	NA	748	mainlib

Compound Structure	Hit Spectrum

10-Heptadecen-8-ynoic acid, methyl ester, (E)-
Formula C18H30O2, MW 278, CAS# 16714-85-5, Entry# 48853
Methyl (10E)-10-heptadecen-8-ynoate #

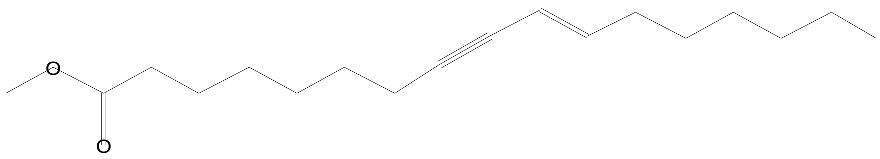

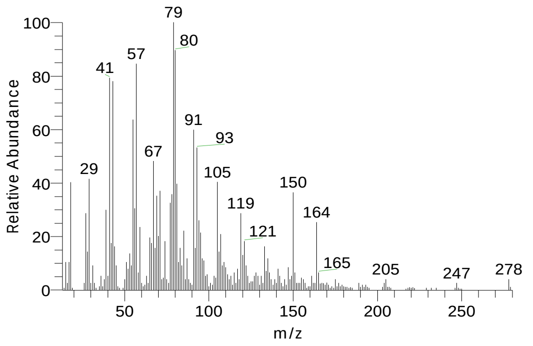

10-HEPTADECEN-8-YNOIC ACID, METHYL ESTER, (E)-
Formula C18H30O2, MW 278, CAS# 16714-85-5, Entry# 169022
METHYL (10E)-10-HEPTADECEN-8-YNOATE #

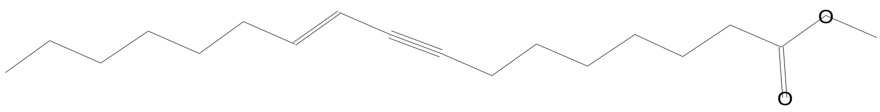

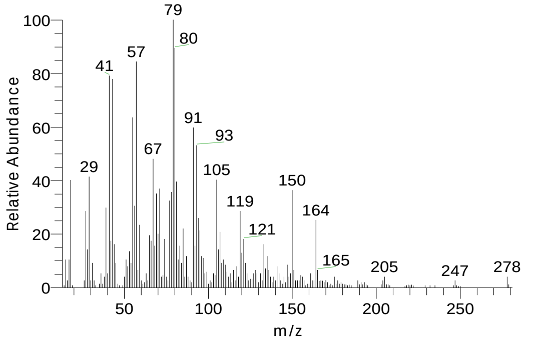

Z,Z,Z-1,4,6,9-Nonadecatetraene
Formula C19H32, MW 260, CAS# NA, Entry# 2508
(4Z,6Z,9Z)-1,4,6,9-Nonadecatetraene #

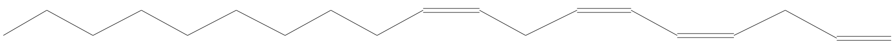

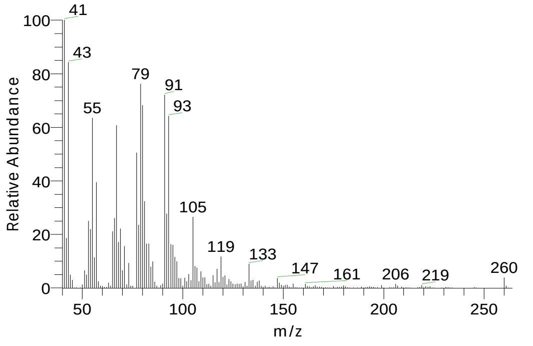

My GC-MS Report

Galrlic_sample #5996 RT: 24.11 AV: 1 NL: 1.26E6
T: + c EI Full ms [50.000-650.000]

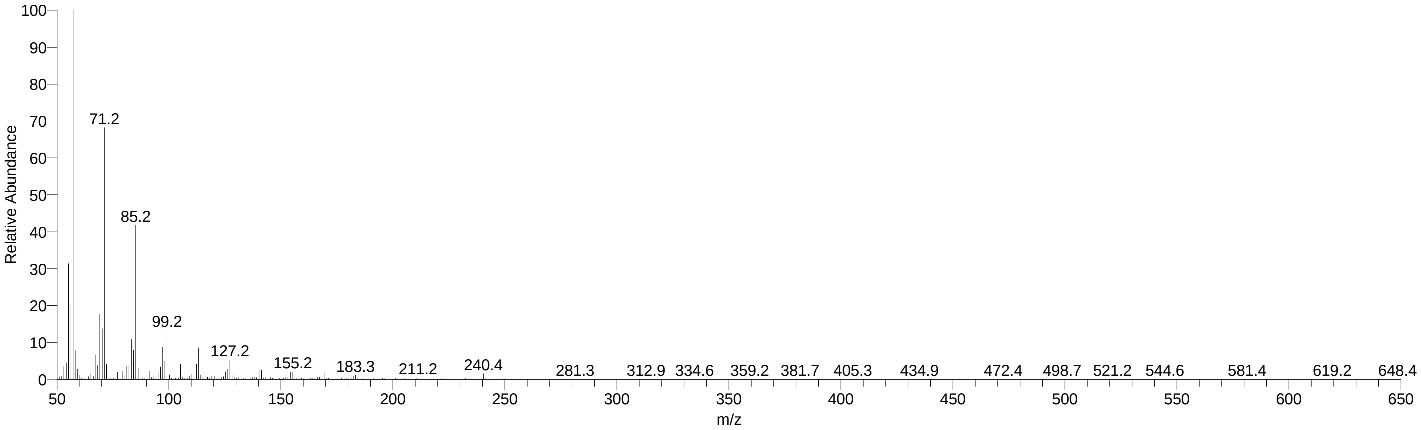

RT	Compound Name	Area %	Molecular Formula	Molecular Weight	Cas #	MF	Library
24.11	Nonadecane	0.66	C19H40	268	629-92-5	943	replib
24.11	HEPTADECANE	0.66	C17H36	240	629-78-7	854	WileyRegistry8e
24.11	Hexadecane	0.66	C16H34	226	544-76-3	907	replib

Compound Structure	Hit Spectrum

Nonadecane
Formula C19H40, MW 268, CAS# 629-92-5, Entry# 6141
n-Nonadecane

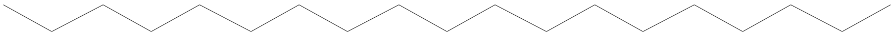

SI 854, RSI 943, replib, Entry# 6141, CAS# 629-92-5, Nonadecane

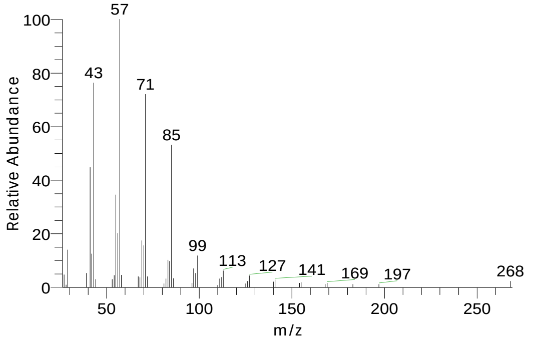

HEPTADECANE
Formula C17H36, MW 240, CAS# 629-78-7, Entry# 130265
AI3-36898

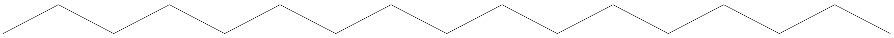

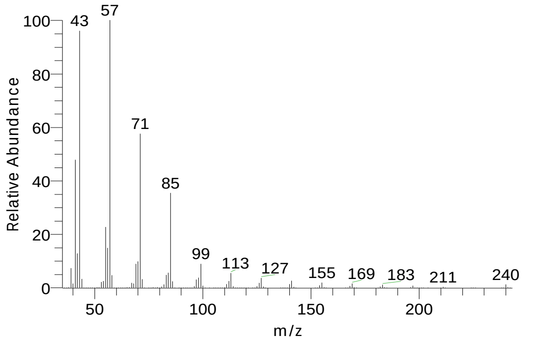

Hexadecane
Formula C16H34, MW 226, CAS# 544-76-3, Entry# 6168
n-Cetane

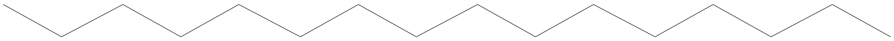

SI 837, RSI 907, replib, Entry# 6168, CAS# 544-76-3, Hexadecane

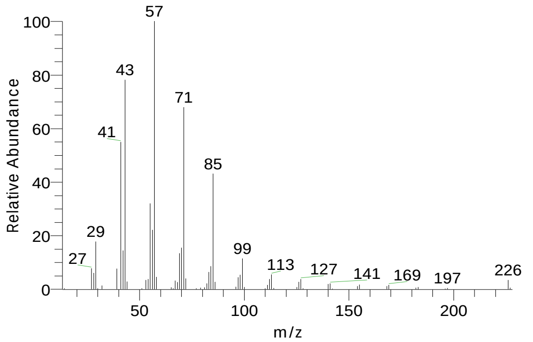

My GC-MS Report

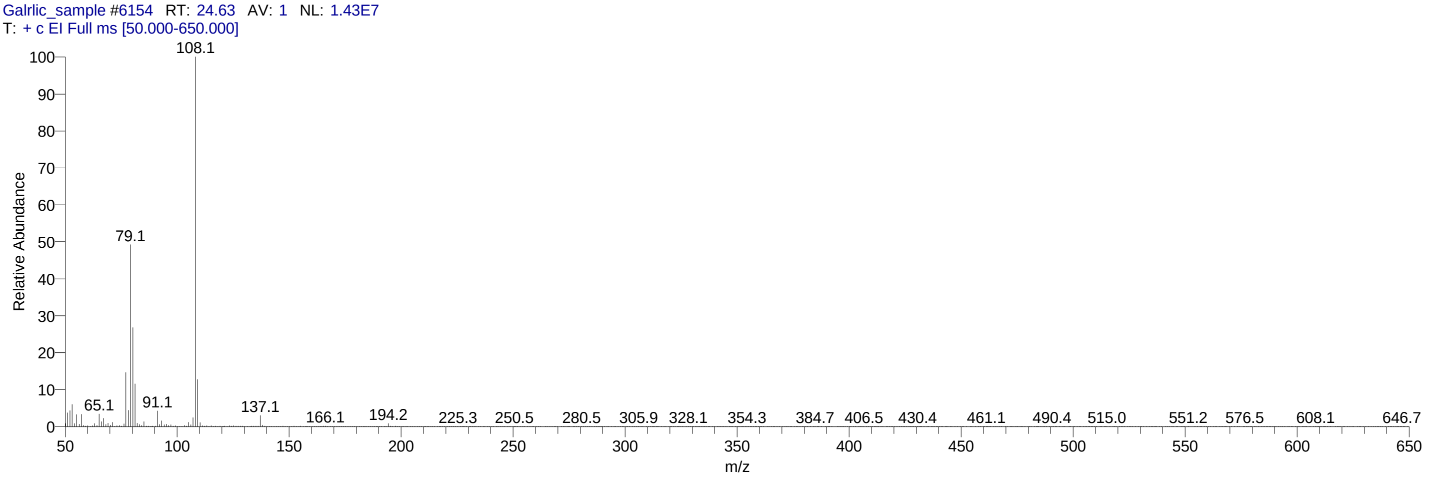

RT	Compound Name	Area %	Molecular Formula	Molecular Weight	Cas #	MF	Library
24.63	(3S,3aS)-3-Butyl-3a,4,5,6-tetrahydroisobenzofuran-1(3H)-one	3.98	C12H18O2	194	124815-25-4	918	mainlib
24.63	(3S,3aR)-3-Butyl-3a,4,5,6-tetrahydroisobenzofuran-1(3H)-one	3.98	C12H18O2	194	4567-33-3	947	mainlib
24.63	3-ISOBUTYLIDENPHTHALIDE	3.98	C12H12O2	188	NA	951	WileyRegistry8e

Compound Structure	Hit Spectrum

(3S,3aS)-3-Butyl-3a,4,5,6-tetrahydroisobenzofuran-1(3H)-one
Formula C12H18O2, MW 194, CAS# 124815-25-4, Entry# 84833
(-)-cis-Neocnidilide

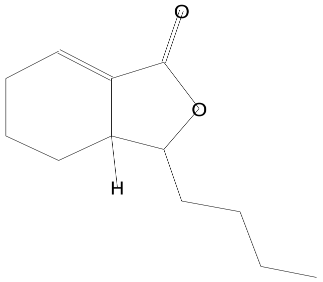

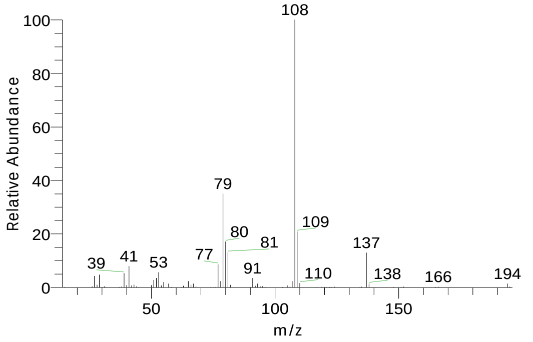

(3S,3aR)-3-Butyl-3a,4,5,6-tetrahydroisobenzofuran-1(3H)-one
Formula C12H18O2, MW 194, CAS# 4567-33-3, Entry# 84820
(3S,3aR)-(-)-Sedanolide

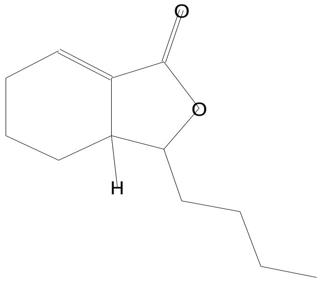

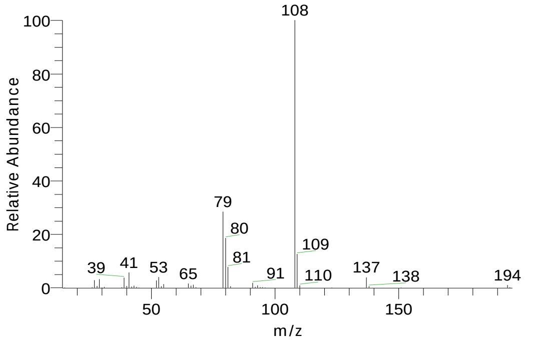

3-ISOBUTYLIDENPHTHALIDE
Formula C12H12O2, MW 188, CAS# NA, Entry# 71488

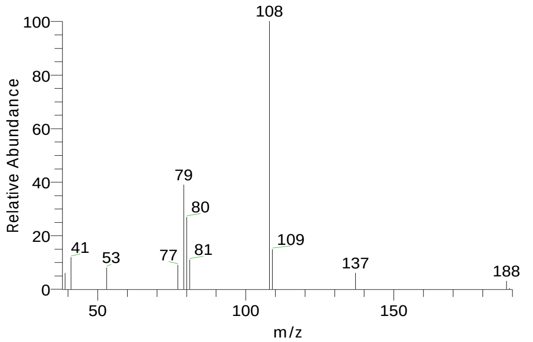

My GC-MS Report

Galrlic_sample #6641 RT: 26.27 AV: 1 NL: 1.60E6
T: + c EI Full ms [50.000-650.000]

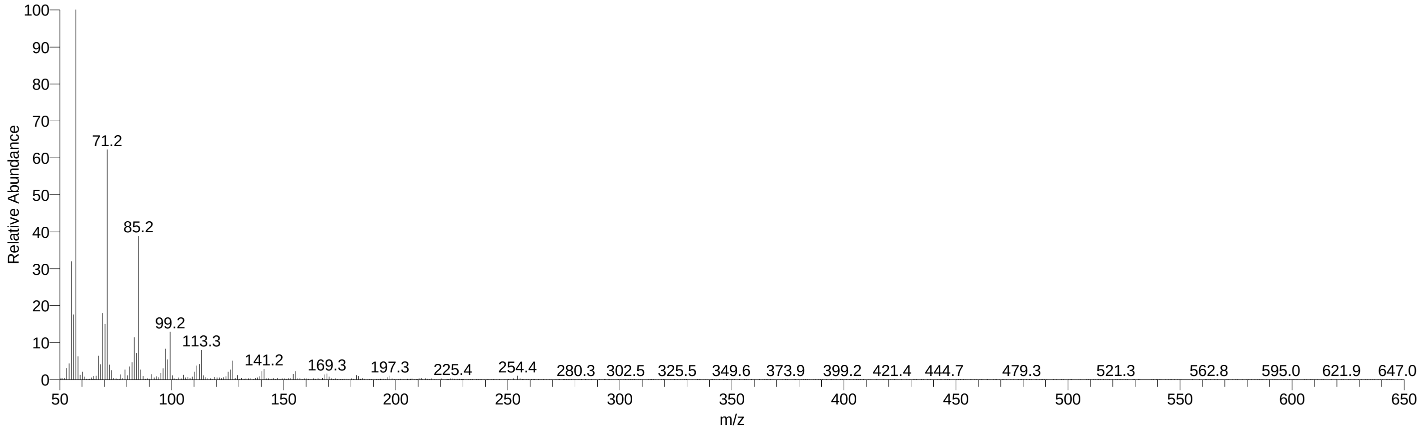

RT	Compound Name	Area %	Molecular Formula	Molecular Weight	Cas #	MF	Library
26.27	Nonadecane	0.57	C19H40	268	629-92-5	945	replib
26.27	Octadecane	0.57	C18H38	254	593-45-3	904	replib
26.27	Heneicosane	0.57	C21H44	296	629-94-7	880	replib

Compound Structure	Hit Spectrum

Nonadecane
Formula C19H40, MW 268, CAS# 629-92-5, Entry# 6141
n-Nonadecane

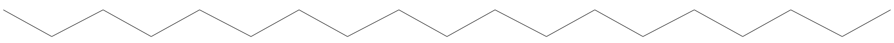

SI 870, RSI 945, replib, Entry# 6141, CAS# 629-92-5, Nonadecane

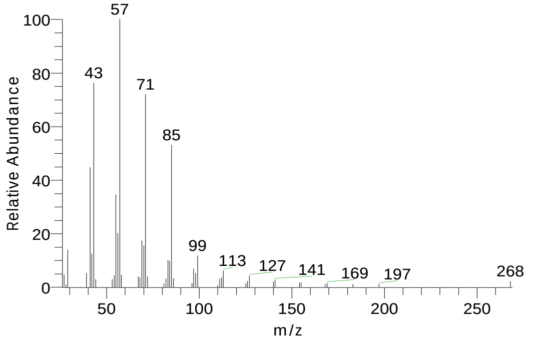

Octadecane
Formula C18H38, MW 254, CAS# 593-45-3, Entry# 6098
n-Octadecane

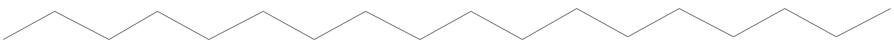

SI 857, RSI 904, replib, Entry# 6098, CAS# 593-45-3, Octadecane

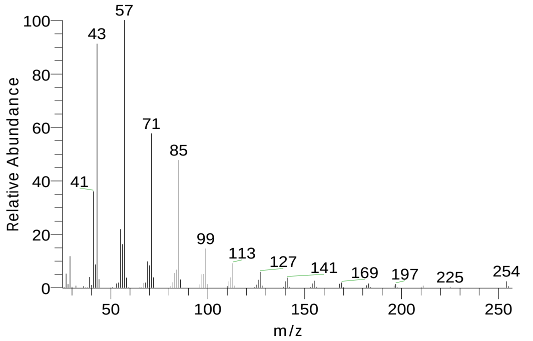

Heneicosane
Formula C21H44, MW 296, CAS# 629-94-7, Entry# 6150
n-Heneicosane

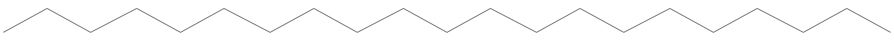

SI 844, RSI 880, replib, Entry# 6150, CAS# 629-94-7, Heneicosane

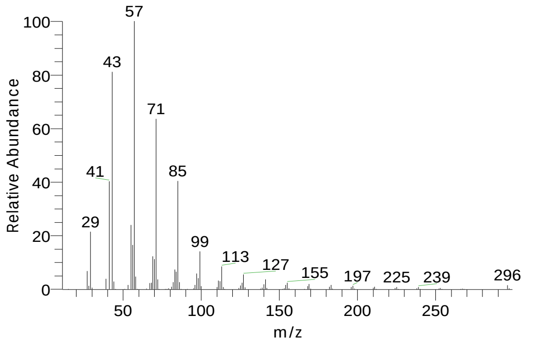

My GC-MS Report

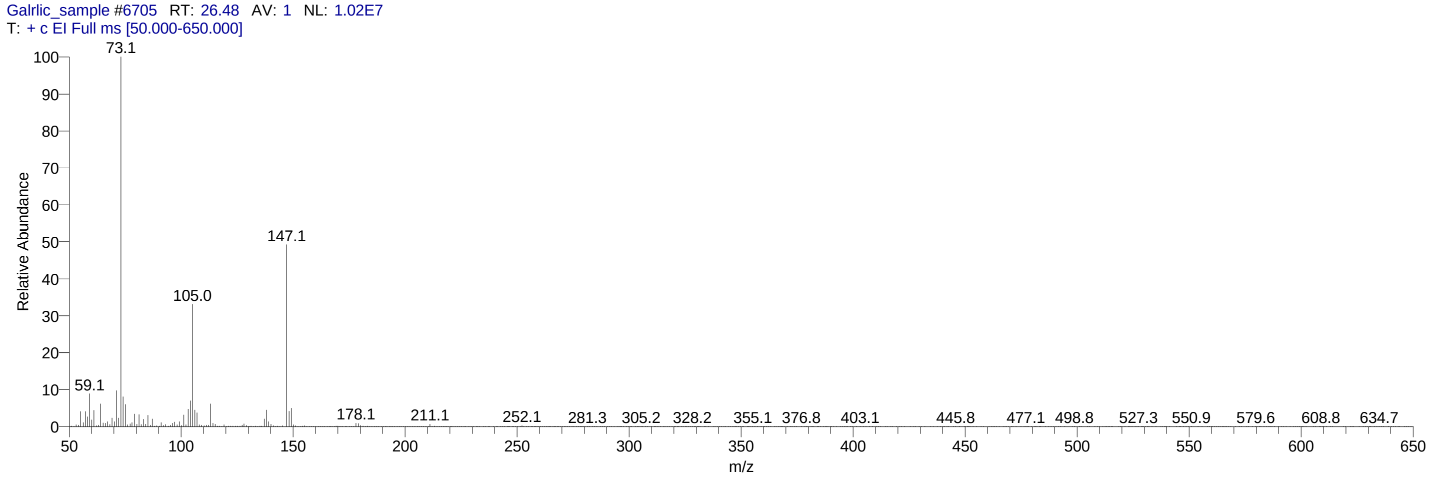

RT	Compound Name	Area %	Molecular Formula	Molecular Weight	Cas #	MF	Library
26.48	1-Allyl-3-(2-(allylthio)propyl)trisulfane	3.46	C9H16S4	252	193625-59-1	918	mainlib
26.48	Disulfide, 1-(1-propenyldithio)propyl propyl, (Z)-	3.46	C9H18S4	254	126876-37-7	752	mainlib
26.48	3-OXA-2,8-DISILANONANE, 2,2,8,8-TETRAMETHYL-	3.46	C10H26OSi2	218	7140-91-2	727	WileyRegistry8e

Compound Structure	Hit Spectrum

1-Allyl-3-(2-(allylthio)propyl)trisulfane
Formula C9H16S4, MW 252, CAS# 193625-59-1, Entry# 40559
Trisulfide, 2-propenyl 2-(2-propenylthio)propyl

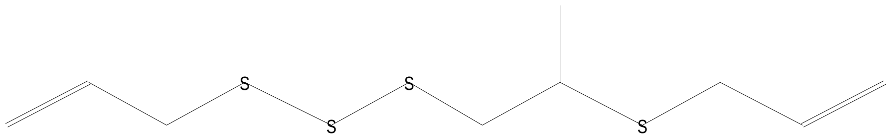

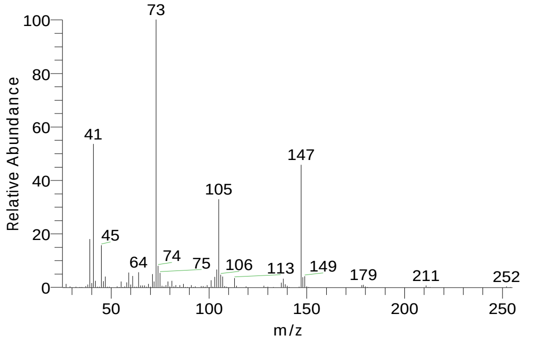

Disulfide, 1-(1-propenyldithio)propyl propyl, (Z)-
Formula C9H18S4, MW 254, CAS# 126876-37-7, Entry# 42039
(Z)-1-(Prop-1-en-1-yl)-2-(1-(propyldisulfanyl)propyl)disulfane

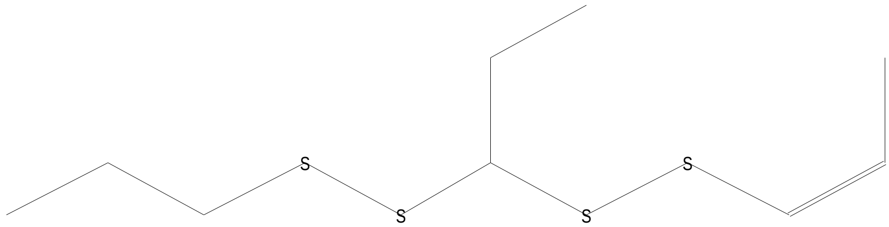

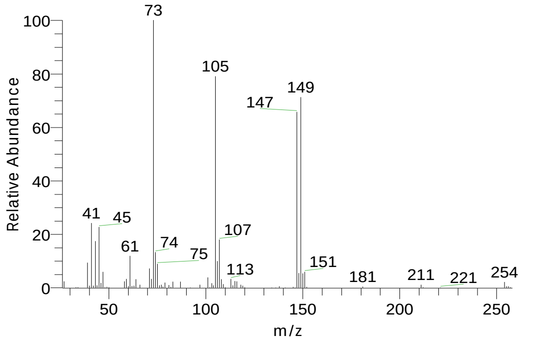

3-OXA-2,8-DISILANONANE, 2,2,8,8-TETRAMETHYL-
Formula C10H26OSi2, MW 218, CAS# 7140-91-2, Entry# 103856
SILANE, TRIMETHYL[4-(TRIMETHYLSILYL)BUTOXY]-

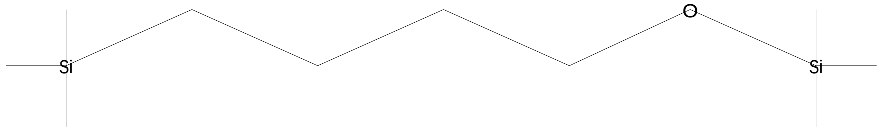

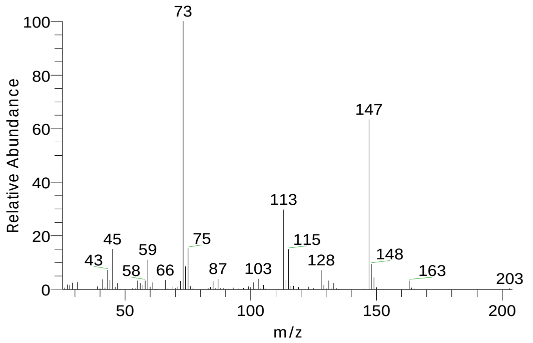

My GC-MS Report

Galrlic_sample #7257 RT: 28.33 AV: 1 NL: 1.21E6
T: + c EI Full ms [50.000-650.000]

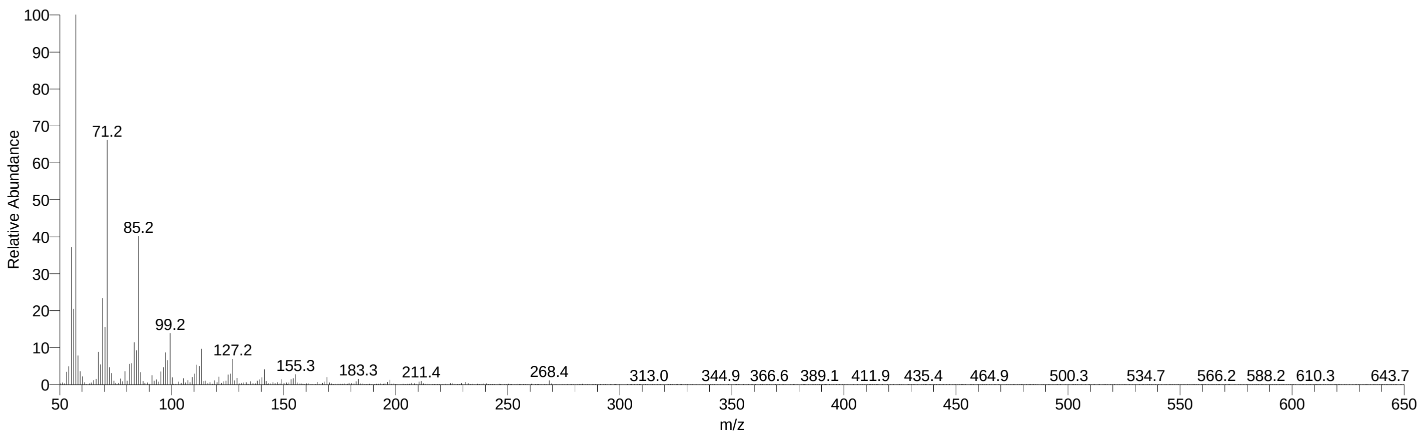

RT	Compound Name	Area %	Molecular Formula	Molecular Weight	Cas #	MF	Library
28.33	Tetradecane, 2,6,10-trimethyl-	0.59	C17H36	240	14905-56-7	844	mainlib
28.33	PENTACOSANE	0.59	C25H52	352	629-99-2	835	WileyRegistry8e
28.33	NONADECANE	0.59	C19H40	268	629-92-5	905	WileyRegistry8e

Compound Structure	Hit Spectrum

Tetradecane, 2,6,10-trimethyl-
Formula C17H36, MW 240, CAS# 14905-56-7, Entry# 24375
2,6,10-Trimethyltetradecane

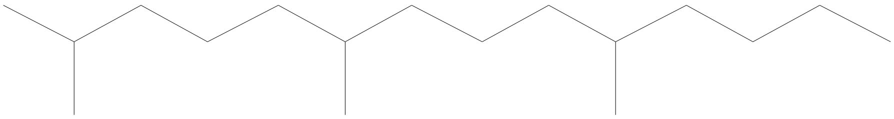

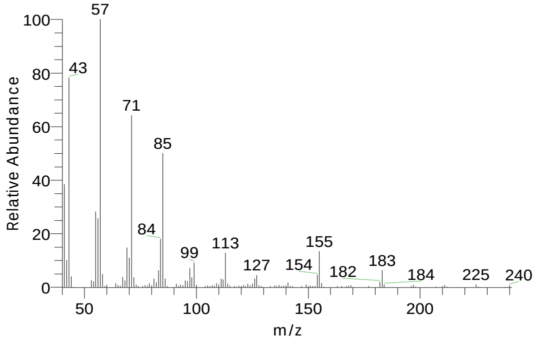

PENTACOSANE
Formula C25H52, MW 352, CAS# 629-99-2, Entry# 230318
AI3-36478

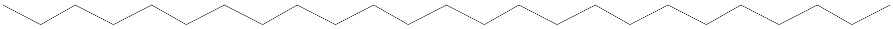

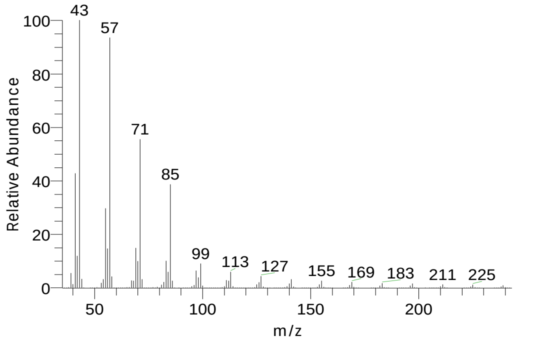

NONADECANE
Formula C19H40, MW 268, CAS# 629-92-5, Entry# 159397
AI3-36122

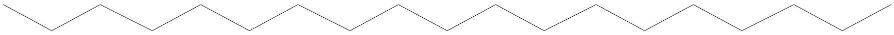

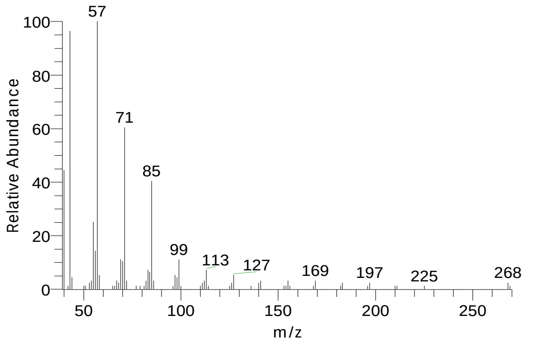

My GC-MS Report

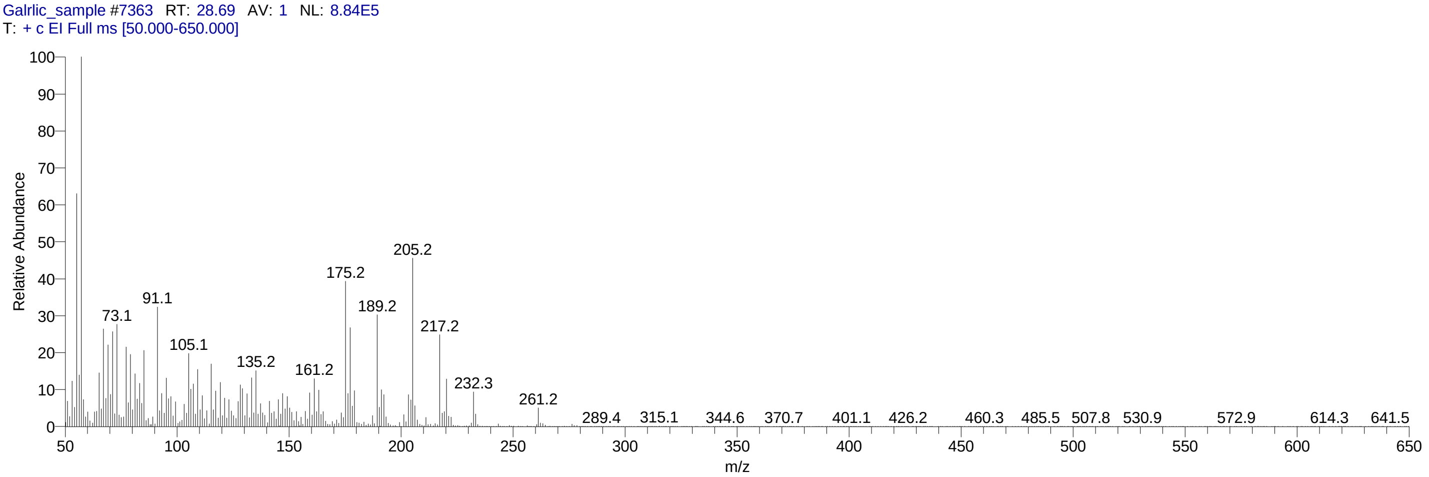

RT	Compound Name	Area %	Molecular Formula	Molecular Weight	Cas #	MF	Library
28.69	7,9-Di-tert-butyl-1-oxaspiro(4,5)deca-6,9-diene -2,8-dione	0.94	C17H24O3	276	82304-6 6-3	890	replib
28.69	7,9-DITERT-BUTYL-1-OXASPIRO[4.5]DEC A-6,9-DIENE-2,8-DIONE	0.94	C17H24O3	276	82304-6 6-3	890	WileyRegi stry8e
28.69	7,9-Di-tert-butyl-1-oxaspiro(4,5)deca-6,9-diene -2,8-dione	0.94	C17H24O3	276	82304-6 6-3	905	mainlib

Compound Structure	Hit Spectrum

7,9-Di-tert-butyl-1-oxaspiro(4,5)deca-6,9-diene-2,8-dione
Formula C17H24O3, MW 276, CAS# 82304-66-3, Entry# 5876
1-Oxa-spiro[4.5]deca-6,9-diene-2,8-dione, 7,9-di-tert-butyl-

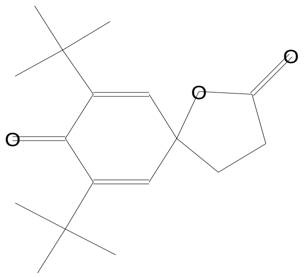

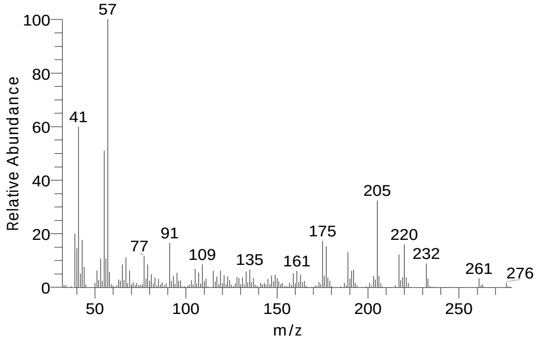

7,9-DITERT-BUTYL-1-OXASPIRO[4.5]DECA-6,9-DIENE-2,8-DIONE
Formula C17H24O3, MW 276, CAS# 82304-66-3, Entry# 311766
1-OXA-SPIRO[4.5]DECA-6,9-DIENE-2,8-DIONE, 7,9-DI-TERT-BUTYL-

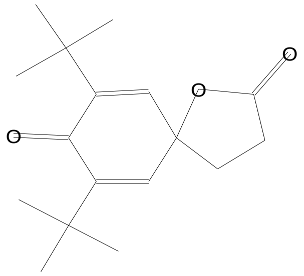

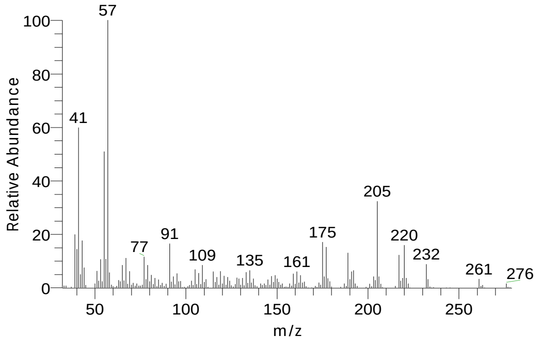

7,9-Di-tert-butyl-1-oxaspiro(4,5)deca-6,9-diene-2,8-dione
Formula C17H24O3, MW 276, CAS# 82304-66-3, Entry# 27271
1-Oxa-spiro[4.5]deca-6,9-diene-2,8-dione, 7,9-di-tert-butyl-

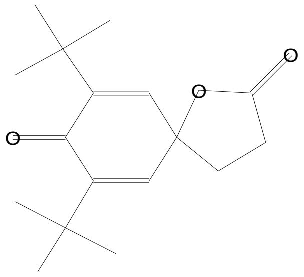

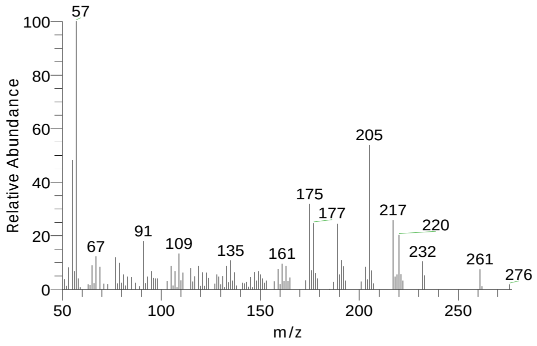

My GC-MS Report

Galrlic_sample #7673 RT: 29.73 AV: 1 NL: 6.76E5
T: + c EI Full ms [50.000-650.000]

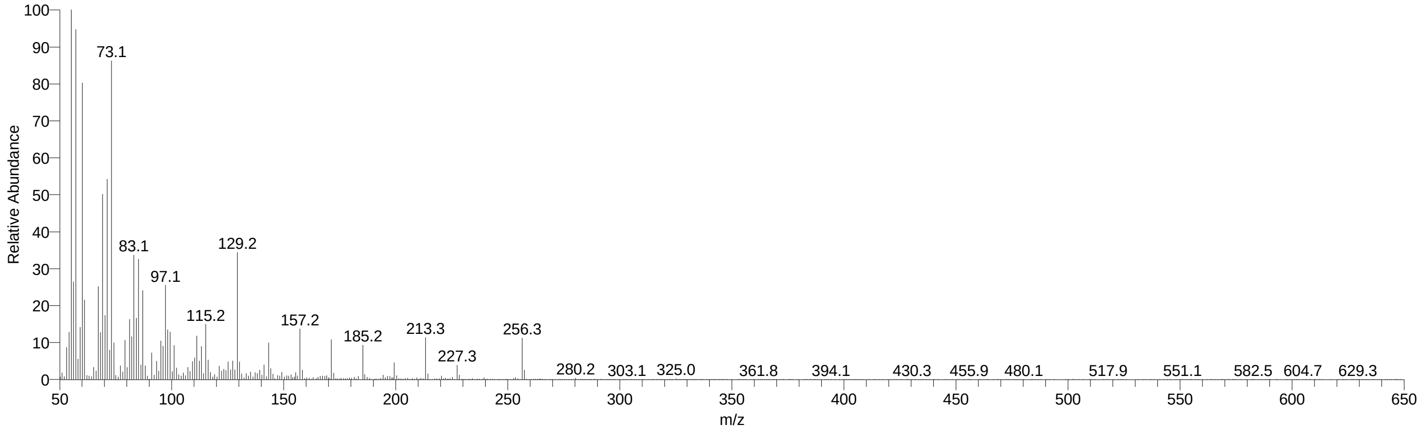

RT	Compound Name	Area %	Molecular Formula	Molecular Weight	Cas #	MF	Library
29.73	HEXADECANOIC ACID	1.20	C16H32O2	256	57-10-3	888	WileyRegistry8e
29.73	n-Hexadecanoic acid	1.20	C16H32O2	256	57-10-3	882	replib
29.73	n-Hexadecanoic acid	1.20	C16H32O2	256	57-10-3	876	mainlib

Compound Structure | Hit Spectrum

HEXADECANOIC ACID
Formula C16H32O2, MW 256, CAS# 57-10-3, Entry# 146746
HEXADECANOATE

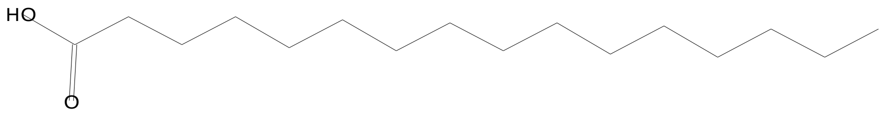

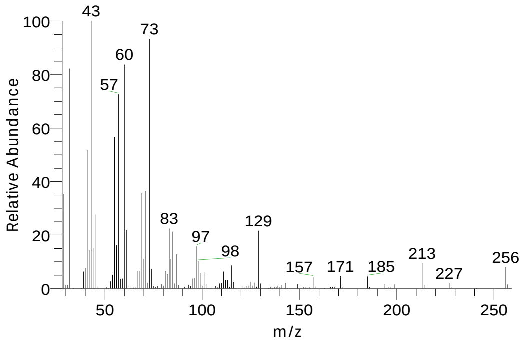

n-Hexadecanoic acid
Formula C16H32O2, MW 256, CAS# 57-10-3, Entry# 7566
Hexadecanoic acid

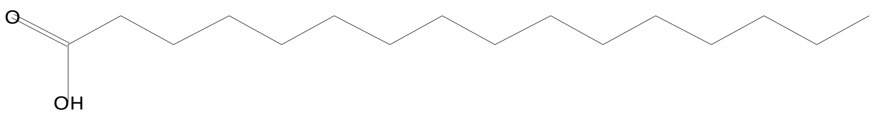

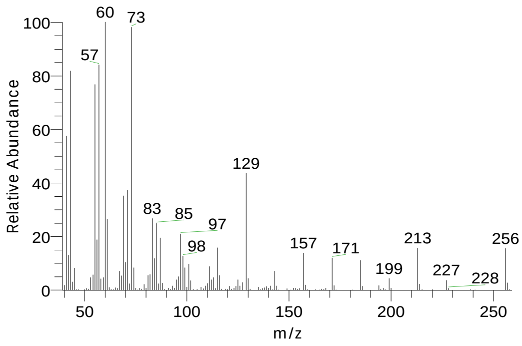

n-Hexadecanoic acid
Formula C16H32O2, MW 256, CAS# 57-10-3, Entry# 9208
Hexadecanoic acid

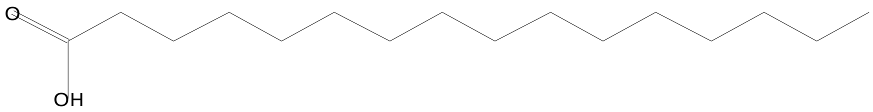

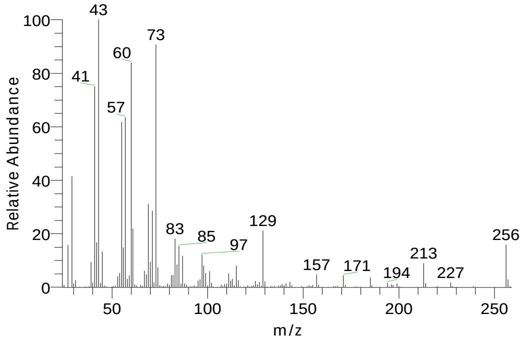

My GC-MS Report

Galrlic_sample #8372 RT: 32.07 AV: 1 NL: 5.56E5
T: + c EI Full ms [50.000-650.000]

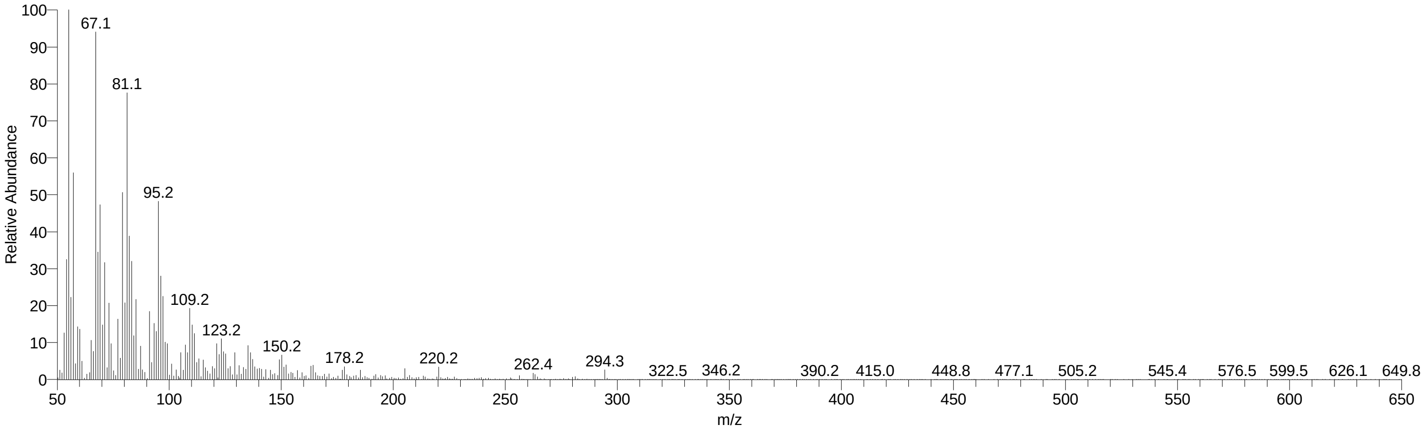

RT	Compound Name	Area %	Molecular Formula	Molecular Weight	Cas #	MF	Library
32.07	ETHYL (9Z,12Z)-9,12-OCTADECADIENOATE #	0.58	C20H36O2	308	544-35-4	861	WileyRegistry8e
32.07	Cyclopropaneoctanoic acid, 2-[[2-[(2-ethylcyclopropyl)methyl]cyclopropyl]methyl]-, methyl ester	0.58	C22H38O2	334	10152-71-3	855	mainlib
32.07	CYCLOPROPANEOCTANOIC ACID, 2-[[2-[(2-ETHYLCYCLOPROPYL)METHYL]CYCLOPROPYL]METHYL]-, METHYL ESTER	0.58	C22H38O2	334	10152-71-3	854	WileyRegistry8e

Compound Structure	Hit Spectrum

ETHYL (9Z,12Z)-9,12-OCTADECADIENOATE #
Formula C20H36O2, MW 308, CAS# 544-35-4, Entry# 196852
9,12-OCTADECADIENOIC ACID (9Z,12Z)-, ETHYL ESTER

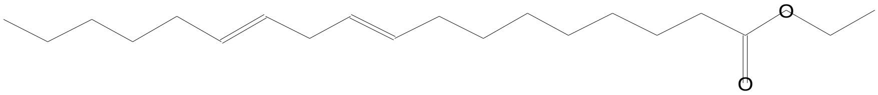

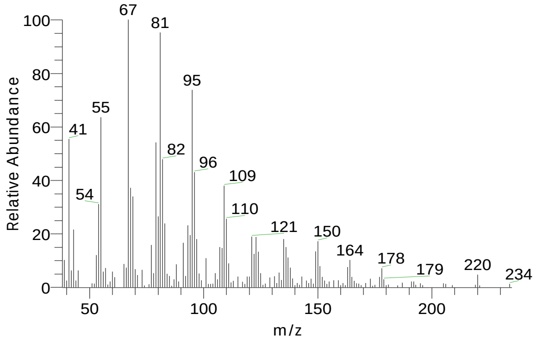

Formula C22H38O2, MW 334, CAS# 10152-71-3, Entry# 2765
Methyl 8-[2-((2-[(2-ethylcyclopropyl)methyl]cyclopropyl)methyl)cyclopropyl]octanoate #

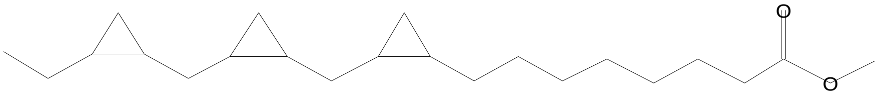

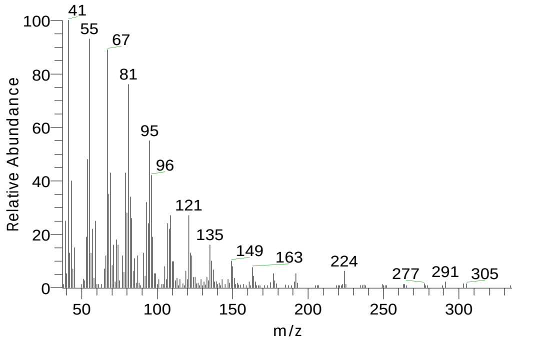

My GC-MS Report

Compound Structure | Hit Spectrum

Formula C22H38O2, MW 334, CAS# 10152-71-3, Entry# 217974

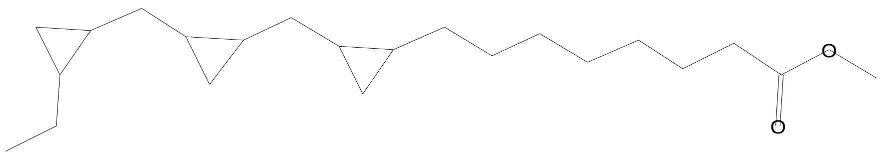

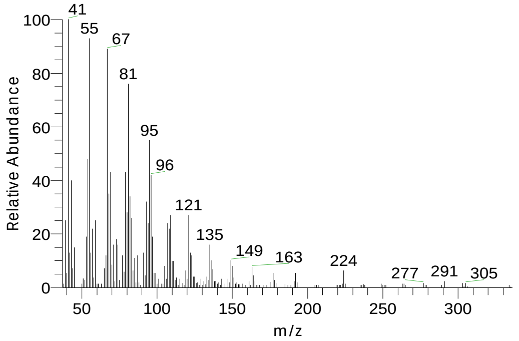

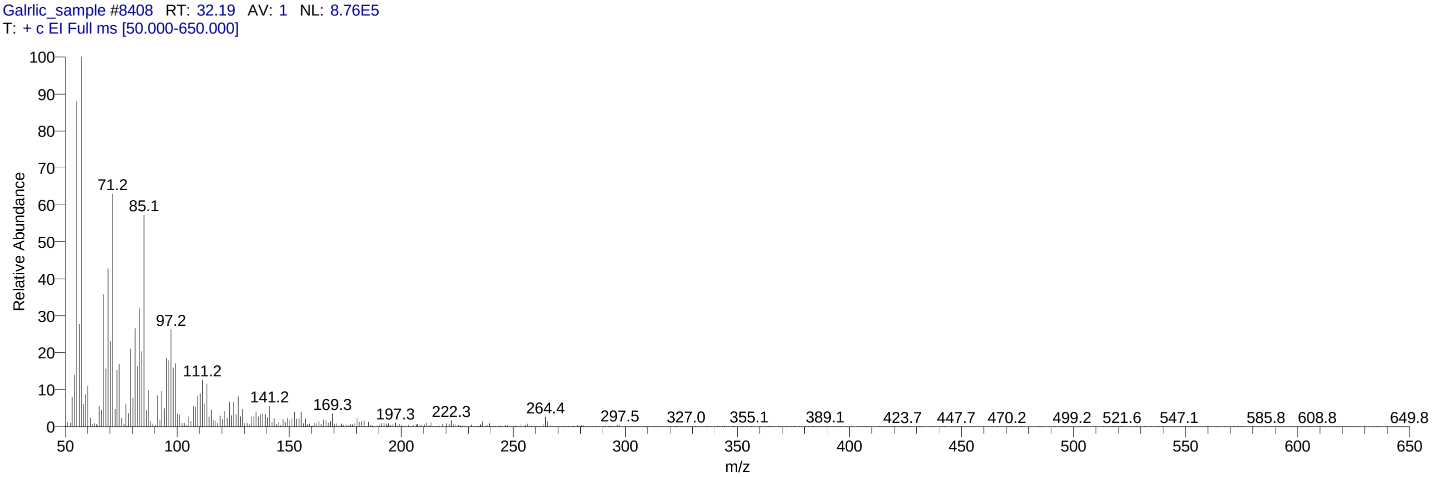

RT	Compound Name	Area %	Molecular Formula	Molecular Weight	Cas #	MF	Library
32.19	9-OCTADECENOIC ACID (Z)-	0.77	C18H34O2	282	112-80-1	827	WileyRegistry8e
32.19	7-Methyl-Z-tetradecen-1-ol acetate	0.77	C17H32O2	268	NA	807	mainlib
32.19	12-Methyl-E,E-2,13-octadecadien-1-ol	0.77	C19H36O	280	NA	815	mainlib

Compound Structure | Hit Spectrum

9-OCTADECENOIC ACID (Z)-
Formula C18H34O2, MW 282, CAS# 112-80-1, Entry# 172910
OCTADEC-9-ENOIC ACID

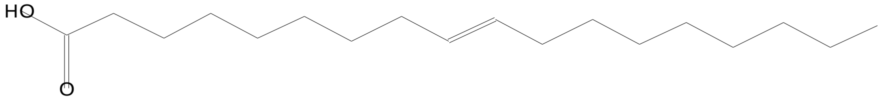

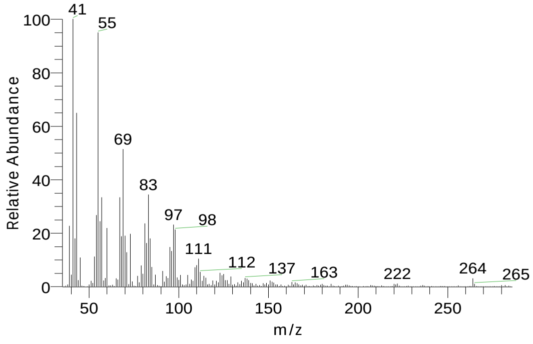

7-Methyl-Z-tetradecen-1-ol acetate
Formula C17H32O2, MW 268, CAS# NA, Entry# 7041
(8Z)-7-Methyl-8-tetradecenyl acetate #

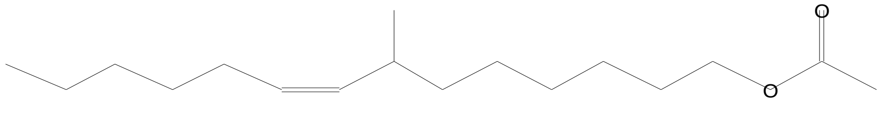

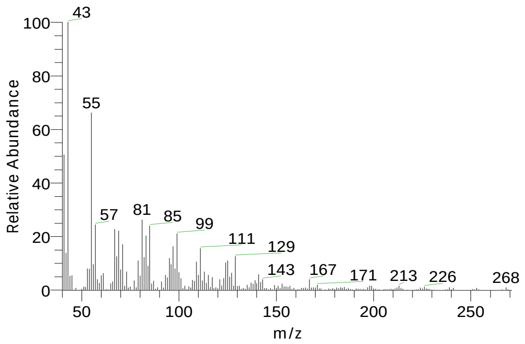

My GC-MS Report

Compound Structure | Hit Spectrum

12-Methyl-E,E-2,13-octadecadien-1-ol
Formula C19H36O, MW 280, CAS# NA, Entry# 19016
(2E,15Z)-14-Methyl-2,15-octadecadien-1-ol #

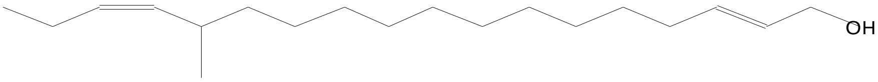

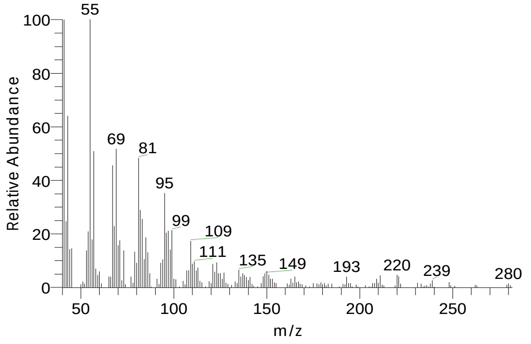

Galrlic_sample #8614 RT: 32.88 AV: 1 NL: 1.10E6
T: + c EI Full ms [50.000-650.000]

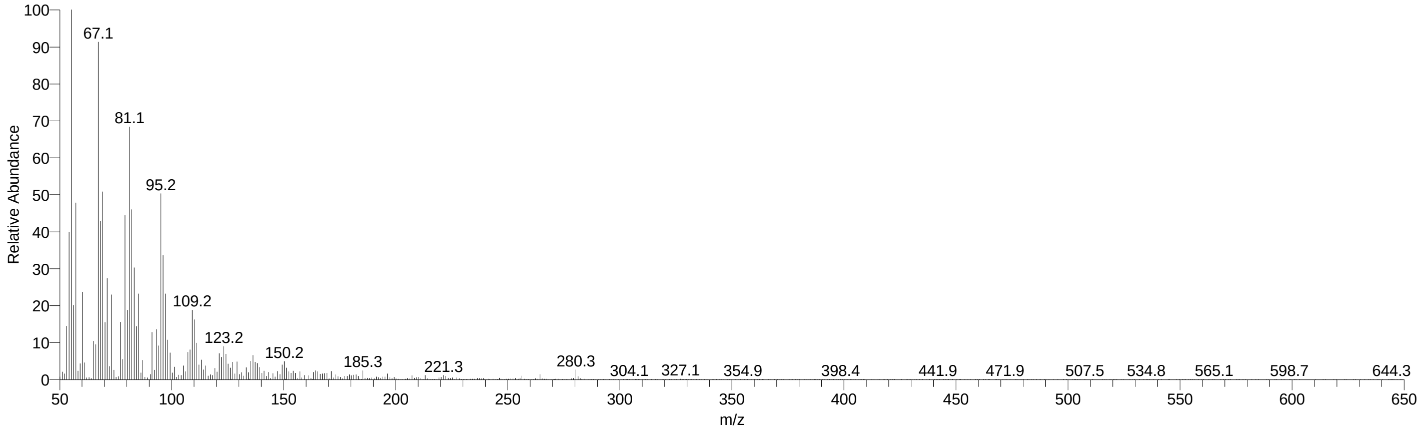

RT	Compound Name	Area %	Molecular Formula	Molecular Weight	Cas #	MF	Library
32.88	9,12-Octadecadienoic acid (Z,Z)-	0.92	C18H32O2	280	60-33-3	872	replib
32.88	9,12-OCTADECADIENOIC ACID (Z,Z)-	0.92	C18H32O2	280	60-33-3	872	WileyRegistry8e
32.88	9,12-OCTADECADIENOIC ACID	0.92	C18H32O2	280	NA	886	WileyRegistry8e

Compound Structure | Hit Spectrum

9,12-Octadecadienoic acid (Z,Z)-
Formula C18H32O2, MW 280, CAS# 60-33-3, Entry# 8057
cis-9,cis-12-Octadecadienoic acid

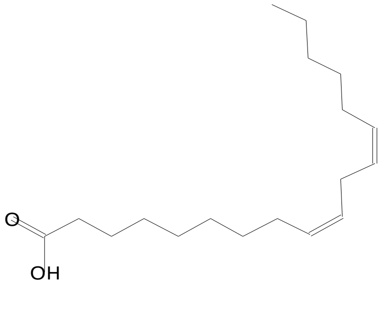

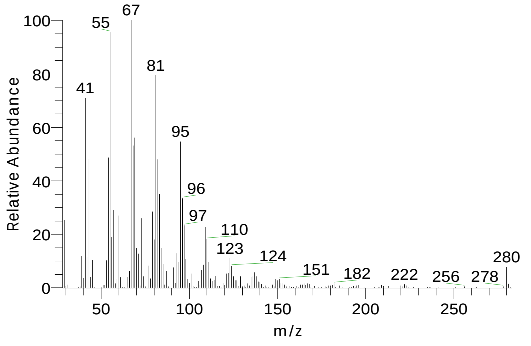

My GC-MS Report

Compound Structure	Hit Spectrum

9,12-OCTADECADIENOIC ACID (Z,Z)-
Formula C18H32O2, MW 280, CAS# 60-33-3, Entry# 170904
(9E,12E)-9,12-OCTADECADIENOIC ACID #

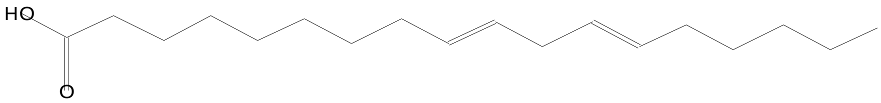

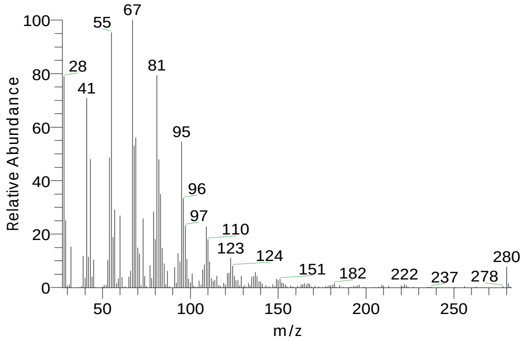

9,12-OCTADECADIENOIC ACID
Formula C18H32O2, MW 280, CAS# NA, Entry# 359462
LINOLSAEURE

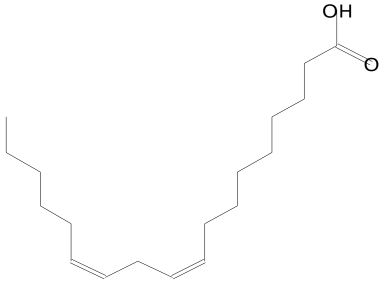

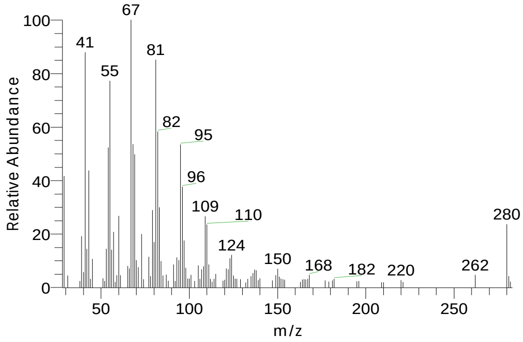

Galrlic_sample #8641 RT: 32.98 AV: 1 NL: 1.82E6
T: + c EI Full ms [50.000-650.000]

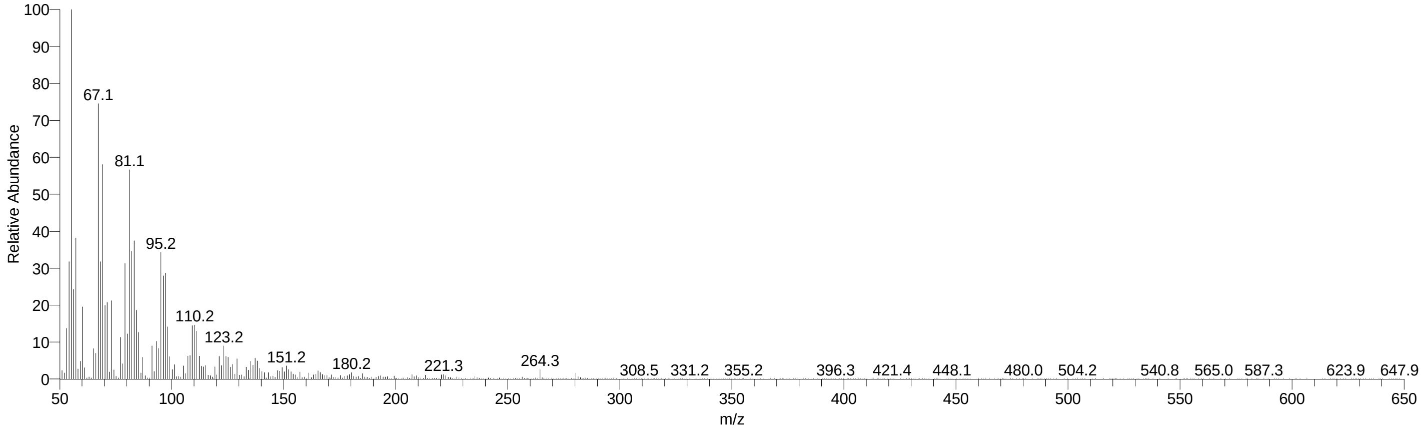

RT	Compound Name	Area %	Molecular Formula	Molecular Weight	Cas #	MF	Library
32.98	9-OCTADECENOIC ACID (Z)-	2.26	C18H34O2	282	112-80-1	884	WileyRegistry8e
32.98	9,12-Octadecadienoic acid (Z,Z)-	2.26	C18H32O2	280	60-33-3	871	replib
32.98	9,12-OCTADECADIENOIC ACID (Z,Z)-	2.26	C18H32O2	280	60-33-3	871	WileyRegistry8e

My GC-MS Report

Compound Structure | Hit Spectrum

9-OCTADECENOIC ACID (Z)-
Formula C18H34O2, MW 282, CAS# 112-80-1, Entry# 172910
OCTADEC-9-ENOIC ACID

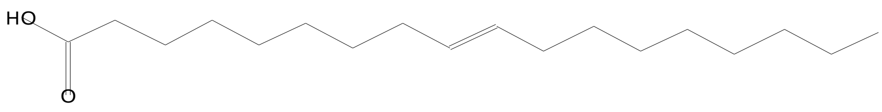

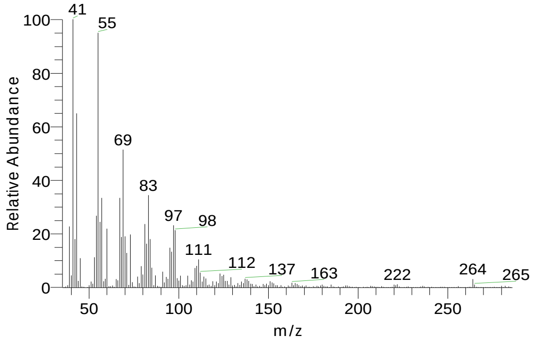

9,12-Octadecadienoic acid (Z,Z)-
Formula C18H32O2, MW 280, CAS# 60-33-3, Entry# 8057
cis-9,cis-12-Octadecadienoic acid

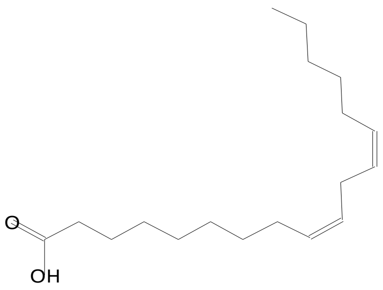

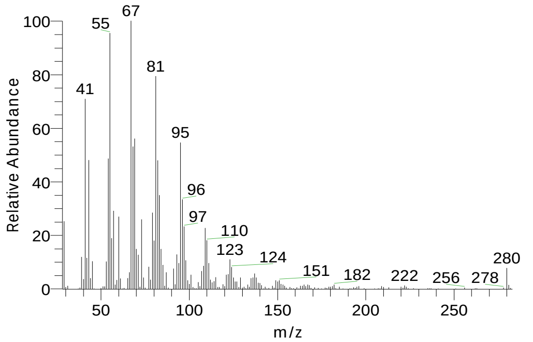

9,12-OCTADECADIENOIC ACID (Z,Z)-
Formula C18H32O2, MW 280, CAS# 60-33-3, Entry# 170904
(9E,12E)-9,12-OCTADECADIENOIC ACID #

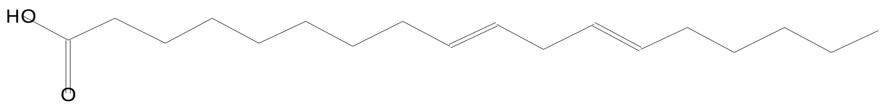

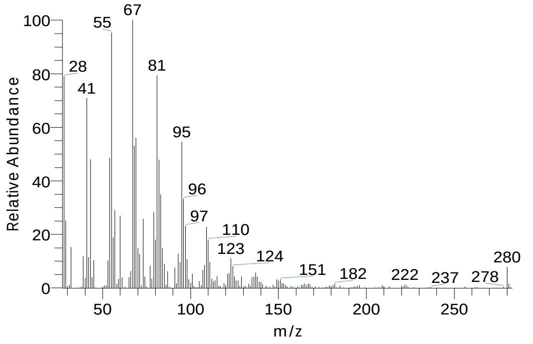

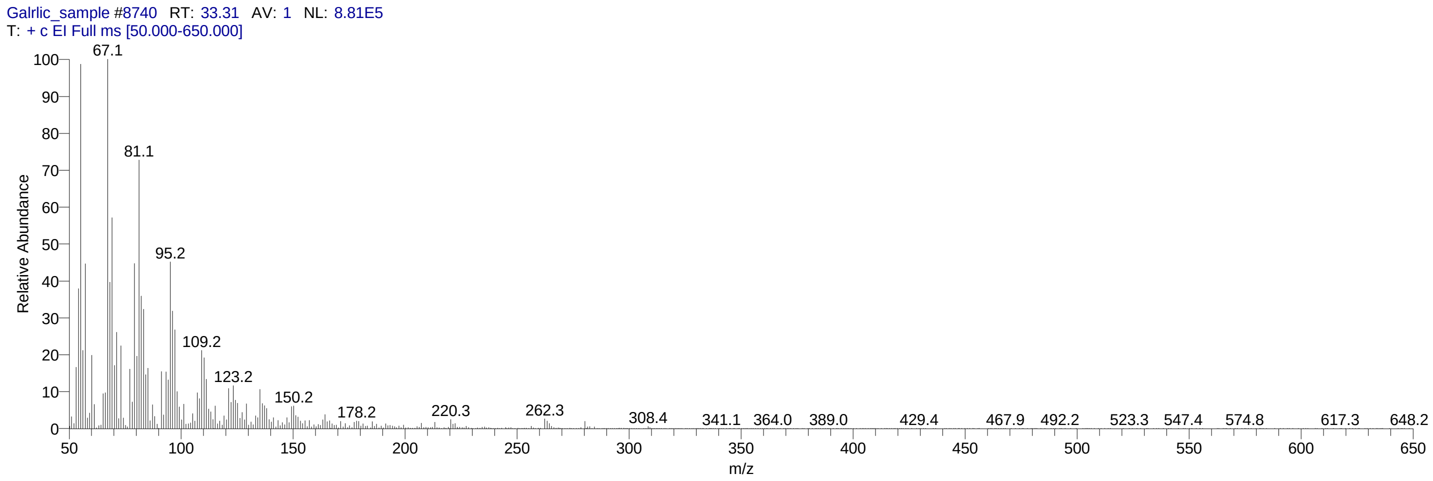

RT	Compound Name	Area %	Molecular Formula	Molecular Weight	Cas #	MF	Library
33.31	9,12-Octadecadienoyl chloride, (Z,Z)-	0.43	C18H31ClO	298	7459-33-8	866	replib
33.31	(9E,12E)-9,12-OCTADECADIENOYL CHLORIDE #	0.43	C18H31ClO	298	7459-33-8	866	WileyRegistry8e

My GC-MS Report

RT	Compound Name	Area %	Molecular Formula	Molecular Weight	Cas #	MF	Library
33.31	ETHYL (9Z,12Z)-9,12-OCTADECADIENOATE #	0.43	C20H36O2	308	544-35-4	877	WileyRegistry8e

Compound Structure | Hit Spectrum

9,12-Octadecadienoyl chloride, (Z,Z)-
Formula C18H31ClO, MW 298, CAS# 7459-33-8, Entry# 4940
Linoleoyl chloride

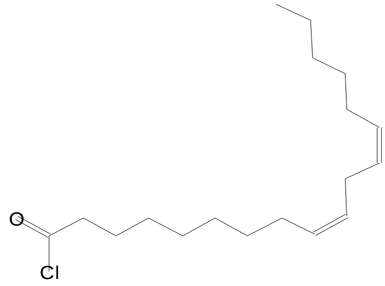

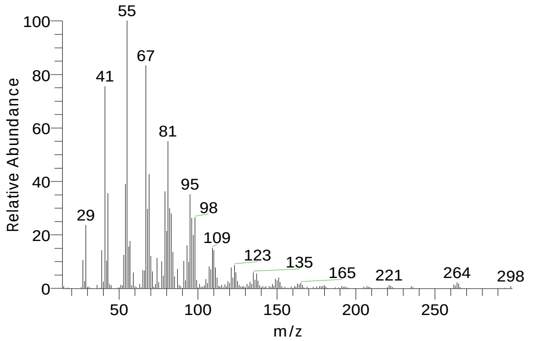

(9E,12E)-9,12-OCTADECADIENOYL CHLORIDE #
Formula C18H31ClO, MW 298, CAS# 7459-33-8, Entry# 187801
(9E,12E)-9,12-OCTADECADIENOYL CHLORIDE

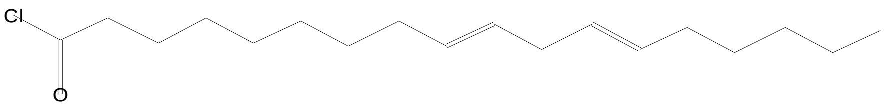

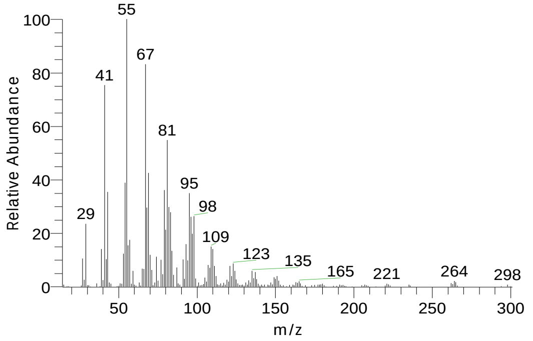

ETHYL (9Z,12Z)-9,12-OCTADECADIENOATE #
Formula C20H36O2, MW 308, CAS# 544-35-4, Entry# 196852
9,12-OCTADECADIENOIC ACID (9Z,12Z)-, ETHYL ESTER

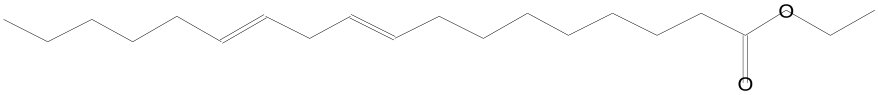

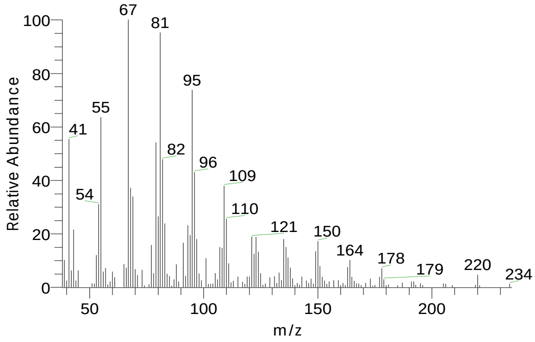

Supplement: Supplementary file 1 [file nutrients-12-01028-s001.pdf]
